# Supplementary material for: Factors related to the development of health-promoting community activities in Spanish primary healthcare: two case–control studies
Source: BMJ Open. 2017 Oct 8;7(10):e015934. doi: 10.1136/bmjopen-2017-015934 (PMC5640008; doi:10.1136/bmjopen-2017-015934)
Supplement: Author's manuscript [file bmjopen-2017-015934.draft_revisions.pdf]

BMJ Open is committed to open peer review. As part of this commitment we make the peer review history of every article we publish publicly available.

When an article is published we post the peer reviewers' comments and the authors' responses online. We also post the versions of the paper that were used during peer review. These are the versions that the peer review comments apply to.

The versions of the paper that follow are the versions that were submitted during the peer review process. They are not the versions of record or the final published versions. They should not be cited or distributed as the published version of this manuscript.

BMJ Open is an open access journal and the full, final, typeset and author-corrected version of record of the manuscript is available on our site with no access controls, subscription charges or pay-per-view fees (http://bmjopen.bmj.com).

If you have any questions on BMJ Open's open peer review process please email editorial.bmjopen@bmj.com

BMJ Open

Factors related to the development of health-promoting community activities in Spanish primary health care: Two case-control studies

Journal:	*BMJ Open*
Manuscript ID	bmjopen-2017-015934
Article Type:	Research
Date Submitted by the Author:	12-Jan-2017
Complete List of Authors:	March, Sebastià; Baleares Health services-IbSalut, Primary Care Research Unit of Mallorca; Instituto de investigación en salud de Palma (IDISPA)<br>Ripoll, Joana; Baleares Health services-IbSalut, Primary Care Research Unit of Mallorca; Baleares Health services-IbSalut, Primary Care Research Unit of Mallorca<br>Jordan Martin, Matilde Jordan Martin; Estrecho de Correa Primary health center, Madrid Health Services<br>Zabaleta del Olmo, Nieves; Fundacio Jordi Gol i Gurina, IDIAP<br>Benedé Azagra, Carmen Belén; Aragon Health Services, Oliver Primary health center<br>Elizalde Soto, Lázaro; Gobierno de Navarra Departamento de Salud, public health institute; Public Health and epidemiology CIBER,<br>Vidal, Mª Clara; Baleares Health services-IbSalut, Primary Care Research Unit of Mallorca; Instituto de investigación en salud de Palma (IDISPA)<br>Bauzà Amengual, M. de Lluc; Balearic Islands University,<br>Planas Juan, Trinidad; Baleares Health services-IbSalut, Son Gotleu Primary health care center<br>Maria Pèrez, Damiana Maria Pèrez; Baleares Health services-IbSalut, Son Gotleu Primary health care center<br>Llull Sarralde, Micaela; Baleares Health services-IbSalut, San Agustí Primary health care center<br>Ruiz-Giménez, Juan Luís; Madrid Health Services, Madrid, Vicente Soldevilla Primary health care center<br>Bajo Viñas, Rosa; Madrid Health care services, Loeches Primary health care center<br>Solano Villarubia, Carmen Solano Villarubia; madrid Health care services, DA Suroeste Health center<br>Rodriguez Bajo, Maria<br>Cordoba Victoria, Manuela Cordoba Victoria; SOMAMFyC<br>Montaner-Gomis, Isabel; Institut Catala De La Salut, El Carmel Health center<br>Badia Capdevila, Marta; Institut Catala De La Salut, El carmel Health center<br>Serrano Ferrandez, Elena; Institut Catala De La Salut, El carmel health center<br>Bosom Diumenjo, Maria; Institut Catala De La Salut, Sant Rafel Primary Health Center<br>Bolibar-Ribas, Buenaventura; Fundacio Jordi Gol i Gurina, IDIAP<br>Antoñanzas Lombarte, Angel; Aragon Health services, Delicias del Sur health center

	Bregel Cotaina, Samantha; Instituto Aragones de Ciencias de la Salud, Primary health care research unit<br>Calvo Tocado, Ana; Zaragoza Public Health provincial subdirection<br>Olivan Blázquez, Barbara; Instituto Aragones de Ciencias de la Salud, primary health care research unit<br>Magallon Botaya, Rosa; Aragon Health services, Arrabal health center<br>Marín Palacios, Pilar Marín Palacios; Gobierno de Navarra Departamento de Salud, Public health institute<br>Echauri Ozcoidi, Margarita; Gobierno de Navarra Departamento de Salud, public health institute<br>Perez-Jarauta, Mª Jose Perez-Jarauta; Gobierno de Navarra Departamento de Salud, public health institute<br>Llobera, Joan; Baleares Health services-IbSalut, 1. Primary Care Research Unit of Mallorca; Instituto de investigación en salud de Palma (IDISPA)<br>Ramos, Maria; Govern de les Illes Balears Conselleria de Salut Familia i Benestar Social, Direcció General de salut Pública i Participació; Instituto de investigación en salud de Palma (IDISPA)
**Primary Subject Heading**:	General practice / Family practice
Secondary Subject Heading:	Health services research, Public health
Keywords:	Health promotion, Primary health care, Health services research, Community health, Health team, Health professionals

SCHOLARONE™ Manuscripts

For peer review only

For peer review only - http://bmjopen.bmj.com/site/about/guidelines.xhtml

Factors related to the development of health-promoting community activities in Spanish primary health care: Two case-control studies

Sebastià March[1], Joana Ripoll[1], Matilde Jordan Martin[2], Edurne Zabaleta del Olmo[3], Carmen Belén Benedé Azagra[4], Lázaro Elizalde Soto[5], Mª Clara Vidal[1], María de Lluc Bauzà Amengual[6], Trinidad Planas Juan[7], Damiana Maria Pérez Mariano[7], Micaela Llull Sarralde[8], Juan Luís Ruiz-Giménez[9], Rosa Bajo Viñas[10] , Carmen Solano Villarubia[11], Maria Rodriguez Bajo[12], Manuela Cordoba Victoria[13], Marta Badia Capdevila[3], Elena Serrano Ferrandez[3], Maria Bosom Diumenjo[14], Isabel Montaner Gomis[15], Bonaventura Bolívar Ribas[3], Angel Antoñanzas Lombarte[16], Samantha Bregel Cotaina[17], Ana Calvo Tocado[18], Barbara Olivan Blázquez[17], Rosa Magallón Botaya[19], Pilar Marín Palacios[5], Margarita Echauri Ozcoidi[5], María Jose Perez-Jarauta[5], Joan Llobera[1], Maria Ramos[20].

1. Primary Care Research Unit of Mallorca, Baleares Health Services-IbSalut, Palma de Mallorca, Spain.
2. Estrecho de Correa Primary Health Center, Madrid Health Services, Madrid, Spain.
3. Primary Care Health Research Institute IDIAP Jordi Gol, Barcelona, Spain.
4. Oliver Primary Health Center, Aragon Health Services, Zaragoza, Spain.
5. Navarra Public Health Institute, and Public Health and Epidemiology CIBER, Pamplona, Spain.
6. Balearic Islands University, Palma de Mallorca, Spain.
7. Son Gotleu Primary Health Center, Baleares Health Services-IbSalut, Palma de Mallorca, Spain.
8. San Agustí Primary Health Center, Baleares Health Services-IbSalut, Palma de Mallorca, Spain.
9. Vicente Soldevilla Primary Health Center, Madrid Health Services, Madrid, Spain.
10. Loeches Primary Health Center, Madrid Health Services, Madrid, Spain.
11. DA Sureste Primary Health Center, Madrid Health Services, Madrid, Spain.
12. Freelance, Madrid, Spain
13. SOMAMFYC, Madrid, Spain
14. Sant Rafel Primary Health Center, Catalonian Health Services Cat-Salut, Barcelona, Spain.
15. El Carmel Primary Health Center, Catalonian Health Services Cat-Salut, Barcelona, Spain.
16. Delicias del Sur Primary Health Center, Aragon Health Services, Zaragoza, Spain.
17. Primary Care Research Unit, Aragon, Spain
18. Zaragoza Public Health department, Zaragoza, Spain.
19. Arrabal Primary Health Center, Aragon Health Services, Zaragoza, Spain.
20. Public Health Department, Balearic Islands Health Department, Spain.

Corresponding author:
Sebastià March
E-mail: smarch@ibsalut.caib.es,

For peer review only - http://bmjopen.bmj.com/site/about/guidelines.xhtml

Abstract

Objective: Spanish primary health care teams have the responsibility of performing health-promoting community activities, although such activities are not widespread. Our aim was to identify the factors related to participation in those activities.

Design: Two case-control studies. **Setting:** Performed in primary care of 5 Spanish regions. **Subjects:** In the first study, cases were teams that performed community activities and controls were those that did not. In the second study (on case teams from the first study), cases were professionals who developed these activities and controls were those who did not. **Main outcome measures:** Team, professional, and community characteristics collected through questionnaires (team managers/professionals) and from secondary sources.

Results: The first study examined 203 teams (103 cases, 100 controls). Adjusted factors associated with performing community activities were percentage of nurses (OR=1.07, 95%CI =1.01-1.14), community socio-economic status (higher *vs.* lower OR =2.16, 95%CI=1.18-3.95), and performing undergraduate training (OR=0.44, 95%CI=0.21-0.93). In the second study, 597 professionals responded (254 cases, 343 controls). Adjusted factors were professional classification (physicians do fewer activities than nurses and social workers do more), training in community activities (OR=1.9, 95%CI =1.2-3.1), team support (OR=2.9, 95%CI =1.5-5.7), seniority (OR=1.06, 95%CI=1.03-1.09), nursing tutor (OR =2.0, 95%CI =1.1-3.5), motivation (OR =3.7, 95%CI=1.8-7.5), collaboration with non-governmental organizations (OR=1.9, 95%CI=1.2-3.1), and participation in neighbourhood activities (OR=3.1, 95%CI=1.9-5.1).

Conclusions: Professional characteristics seem to have a greater influence than team/community factors in performing health-promoting community activities. In contrast to

For peer review only - http://bmjopen.bmj.com/site/about/guidelines.xhtml

the opinion expressed by many professionals, workload is not related to performance of health-promoting community activities.

Keywords: Health promotion, Primary health care, Health services research, Community health, Health team, health professionals

Strengths and limitations of this study

- This study examined a topic relatively unexplored, as community health promotion by primary health care.

- A case-control approach was chosen, where cases were subjects (professionals or teams) who were developing community activities, and controls those who not.

- Design allows to explore many distinct factors simultaneously, from different sources, and in two levels: Health team and professional.

- An operative definition of community activities was used, which recognizes the heterogeneity of health promotion interventions developed with the community in Spanish primary health care.

- Because of its observational design, there are limitations to establishing causal inferences.

For peer review only

Introduction

The goal of the Ottawa Charter for Health Promotion is to enable people to increase control over and improve their health. One way to do this is by community activities such as organizing walking groups in a neighbourhood or holding meetings with local authorities or associations to develop specific health programs. Experts at numerous international conferences have agreed on the importance of health-promoting community activities for improving public health[1-3], and there is some evidence that these activities are effective[4].

Since the implementation of health care reform in Spain during the 1980's[5-6], primary health care teams have had responsibility for carrying out health promotion activities. These teams, located in health care centres throughout the country, are composed of physicians, community nurses, paediatricians, midwives, social workers, and other health care professionals (gynaecologists, psychologists, and psychiatrists) in a system that offers almost-universal and free health care. These conditions are ideal for an interdisciplinary approach to encourage health promotion in the community. However, as in other countries[7], implementation of these interventions differs greatly among the different health care teams[8-10], and relatively few professionals are involved[10-12].

There has been limited study of factors related to the development of health-promoting interventions in the community. These factors may be broadly classified as related to the community, to the team, or to the individual professionals. With regard to community factors, some studies indicated that the rural/urban environment[13], size of the municipality[14], level of social interaction within the community[14-16], and presence of a focal point for participation in community health initiatives[14-15] influence the practice of community activities. The health team factors include job satisfaction[17], management of time and setting within the center[13], composition and organization of the team[14-15], duration of operation[14], intra-team support[16], and work burden[7,18]. Studies of professionals have

For peer review only - http://bmjopen.bmj.com/site/about/guidelines.xhtml

highlighted sex[18-20], age[14,19], professional status[13], specific training[21-24], motivation[16-17], and the model applied (biomedical or psychosocial)[17]. The attitudes and beliefs of the professionals, such as trust in the effectiveness of preventive actions[17,21,24,25], self-reported efficacy in carrying out activities[7,21-22], or to support community participation[14,20] are also important. An additional element is professionals' social sensitivity, as evaluated through their personal political leanings[27]. According to professionals themselves, the most important factor is the lack of time available to assume the care burden[18,21,23-26]. All these features have been observed separately, mostly in descriptive studies.

This study presents some of the results of the Factors Related to Health-Promoting Community Activity Development in Primary Care (frAC) Project, whose complete methodology and aims were described elsewhere[28]. The purpose of this study is to identify team and community factors related to implementation of community activities in primary care and factors that may explain why only some professionals within a team participate in community activities.

Methods

Two case-control studies were performed. In the first, the cases were the teams that performed community activities and the controls were those that did not. The second study examined teams that implemented community activities to adjust for community/team factors; in this second analysis the cases were professionals who developed these activities and the controls were those who did not. The study was set in primary health care settings in five regions of Spain (Balearic Islands, Catalonia, Aragon, Madrid, and Navarra). Data collection was carried out in 2009-2010.

Selection of cases and controls

For peer review only - http://bmjopen.bmj.com/site/about/guidelines.xhtml

We telephoned all the health centres in the research areas asking if they had participated in community activities during the last year. If they did, we contacted a participant of the activity and gave him/her a questionnaire to confirm that the activity met our inclusion criteria. For the first study, health teams that participated in at least one community activity in the last year were selected as cases, and controls were those who had not participated. For the second study, we selected all professionals who participated in the planning of community activity as cases and professionals who did not participate in the planning as controls. In every health team we selected at least one control by every case.

A group of experts reached consensus on a conceptual definition of health promoting community activity[29] which was converted into an algorithm for use in this study[28]. In particular, this activity had to be a non-isolated activity carried out in the previous year, in which the professional participated on behalf of the health centre. Furthermore, it had to have involvement and active participation of the community, or the population had to have the capacity to influence the intervention, or it was a cross-sector activity involving collaboration with entities outside of health care (*e.g.* education, social services).

Variables

Information was gathered through three distinct questionnaires (available on request) that were given to the community, the team, and the professionals[28]. The data collected from the community (collected from secondary sources and from questionnaires to centre managers) were demographic composition; socio-economic level; degree of social interaction; health centre setting; geographical dispersion based on an ordinal variable used by the National Health System for resource allocation (from G1 [less dispersed] to G4 [more dispersed]); and existence of health boards (community participation agencies described in health legislation)[15].

For peer review only - http://bmjopen.bmj.com/site/about/guidelines.xhtml

The data collected from the team (collected from secondary sources and questionnaires to centre managers) were: percentages of different professionals (nurses, physicians, and paediatricians); year of opening; population of service area; professional/population and doctor/nurse ratios; availability of a space in the centre to carry out group activities and presence of a team member responsible for health education; collaboration with the area health board where applicable; training of resident physicians; nurses and nursing or physiotherapy students; evaluation of professional relationships between distinct professional disciplines (based on a Likert-type scale ranging from 1 [poor] to 5 [excellent]).

The data collected from the professionals (from individual self-administrated questionnaires) were: sex; age; profession; working situation; health care burden; size of quota allocated; average number and duration of consultations per day at the centre or at home; tutoring; participation in research or specific training in health promotion in the previous five years; years since graduation; years worked in primary care and as a member of the current health care team; autonomy to organize scheduling; arrangements with colleagues to cover health care tasks while carrying out community activities; support of fellow team members in conducting these activities; working atmosphere; job satisfaction; and self-assessed efficacy in conducting community work (based on a Likert-type scale ranging from 1 [poor] to 5 [excellent]).

Opinions and attitudes were measured by responses to various statements developed by the research team, using a Likert-type scale with four options ranging from “strongly agree” to “strongly disagree”. The statements dealt with trust in the effectiveness of the community activities, motivation to carry out these activities, citizen participation in health care decision-making, need to strengthen agencies involved in citizen participation in health care, responsibility for primary care in the community, definition of professional role, link

For peer review only - http://bmjopen.bmj.com/site/about/guidelines.xhtml

between health education and community participation, and the effectiveness of the boards in driving community activities. A four-item validated scale was used to evaluate each professional's clinical practice style (biomedical-psychosocial)[31-32] by assessing the attitude towards the demands of psychosocial and clinical care at work. Finally, social sensitivity data were collected through proxy variables: participation during the previous twelve months in activities in the neighbourhood where the professional works; involvement in any NGO or civil association; and political tendency (assessed through a visual scale adapted from the Centre for Sociological Research[33] Spanish monthly barometer).

Calculation of sample size

For the first study, we calculated that at least 91 teams would be needed in each group based on an OR of 2.5, an alpha risk of 0.05, a beta of 0.2, and an expected proportion of 0.5 in the controls. For the second study, we calculated that at least 222 professionals would be needed in each group based on an OR of 1.75, an alpha risk of 0.05, a beta of 0.2, and an expected proportion of 0.5 in the controls.

Data analysis and management

A descriptive analysis of all variables was carried out to compare their distributions in each group. Categorical variables are expressed as percentages and continuous variables as means with 95% confidence intervals (95% CIs) or as medians and percentiles depending on the distribution. The relationship with the main variable was assessed using the chi-squared test for categorical variables and Student's *t*-test and the Mann-Whitney U non-parametric test for continuous variables. The strength of an association was expressed through unadjusted OR and its 95% CI.

We also performed a logistic regression analysis to calculate adjusted ORs. A saturated model was constructed using each variable whose *p*-value was less than 0.2 in the bivariate analysis. From this model, we tested and compared variations using the maximum

For peer review only - http://bmjopen.bmj.com/site/about/guidelines.xhtml

likelihood method, eliminating variables that had *p*-values less than 0.05 in the Wald test. The collinearity of the variables was examined and interactions tested. we established different models for the health care teams and for the professionals. All analyses were conducted using SPSS ver. 14 and Epidat ver. 3.1 software.

Ethical aspects

The study was approved by the research commissions in all areas where it was performed and by the Research Ethics Committee in Madrid (Area 1) and the University Research Institute in Primary Care (IDIAP) Jordi Gol in Barcelona. The questionnaire was completed anonymously by the professionals, with the identity of individuals and centres coded to ensure blinding of the researchers.

Results

Factors related to the health care team

We examined 203 health care teams (103 cases [50.7%] and 100 controls) in the first study. Table 1 shows the results of the initial bivariate analysis. The variables significantly related to team involvement in community activities were the socioeconomic level of the community, collaboration on training in physiotherapy or nursing, participation in a health board, percentage of nurses in the team, patient/physician ratio, and patient/nurse ratio.

For peer review only

Table 1. Relationship of community and primary health care team (PHT) characteristics with performance of health-promoting activities in the community.

Variable	PHT cases N (%)	PHT controls N (%)	Unadjusted OR (95% CI)
**Health Care setting**			
Urban	73 (71.6)	68 (68.0)	Ref.
Rural	29 (28.4)	32 (32.0)	0.84 (0.46-1.54)
**Socioeconomic level**			
High and medium-high	52 (51)	65 (65)	1
Medium-low and low	50 (49)	35 (35)	**1.79 (1.01-3.14)**
**Degree of social interaction**			
High	22 (23.9)	23 (26.1)	Ref.
Medium	43 (46.7)	35 (39.8)	1.28 (0.61-2.68)
Low	27 (29.3)	30 (34.1)	0.94 (0.43-2.06)
**Population of municipality**			
>1 million	47 (45.6)	48(48)	Ref.
500,000-1 million	7 (12.6)	7 (7)	1.90 (0.70-5.18)
10,000-500,000	22 (21.4)	33 (33)	0.68 (0.35-1.33)
<10,000	21 (20.4)	12 (12)	1.79 (0.79-4.04)
**Degree of geographical dispersion**			
G1 (less dispersed)	62 (66.7)	60 (63.8)	Ref.
G2	11 (11.8)	17 (18.1)	0.63 (0.27-1.45)
G3	9 (9.7)	11 (11.7)	0.79 (0.31-2.05)
G4 (more dispersed)	11 (11.8)	6(6.4)	1.77 (0.67-5.10)
**Active health board**	67 (66.3)	60 (61.9)	1.21 (0.68-2.17)
**Training centre**			
Resident Doctors	35 (34.7)	34 (34.3)	1.01 (0.56-1.82)
Resident Nurses	23 (23.5)	29 (30.2)	0.71 (0.37-1.34)
Nursing/physiotherapy students	73 (72.3)	84 (85.7)	**0.43 (0.21-0.89)**
**Space for group activities**	83 (81.4)	79 (79)	1.16 (0.58-2.32)
**Health education manager**	45 (44.1)	46 (46)	0.93 (0.53-1.61)
**PHT participation in health board**	56 (83.6)	38 (63.3)	**2.95 (1.28-6.78)**
**Continuous variables**	**Mean (95% CI)**	**p***	
PHT operational years	14.3 (13-15.2)	12.9 (11.7-14.1)	.075
Total No. professionals	36.4 (33.1-39.6)	37.5 (34-41.1)	.486
% nurses	35.4 (34.5-36.4)	33.5 (32.4-34.5)	**.010**
% paediatricians	7.0 (6.3-7.7)	7.4 (6.7-8.1)	.450
Population covered by PHT	20031 (18059-22002)	21770.4 (19530-24010)	.247
	**Median (IQR)**	**p****	
% doctors	33.3 (29.5-38.3)	33.3 (29.9-36.4)	.532
% non-EU immigrants	11.1 (5.8-16.2)	11.1 (8.6-15.7)	.690
% population <15 years	13.0 (11.6-14.7)	12.9 (11.3-14.4)	.352
% population >65 years	17.8 (15.0-21.8)	18.9 (13.8-21.5)	.976
% population women	51 (49.5-53)	51.2 (50.3-53.1)	.403
Population ratio registered/professional	524 (444-658)	578 (482-669)	.122
Population ratio registered/doctor	1659 (1454-1905)	1734 (1533-2040)	**.034**
Population ratio registered/nurse	1508 (1284-1847)	1723 (1434-2057)	**.008**
Doctor/nurse ratio	.94 (.86-1.09)	1 (.89-1.14)	.085
**Professional relationships**			
Doctor::nurse	4 (3-4)	4 (3-4)	.376
Doctor::social worker	4 (3-5)	4 (3-4)	.978
Nurse::social worker	4 (3-5)	4 (3-4)	.845

For dichotomous variables, the reference value is always absence of the variable. Data from Spain 2009.* Student's t-test P. ** Mann-Whitney U test P. Bold-face: $p \leq 0.05$.

For peer review only - http://bmjopen.bmj.com/site/about/guidelines.xhtml

Eleven teams (5.0%) were excluded from the logistic regression analysis due to missing data. The 3 variables remaining in the final model were percentage of nurses in the team (OR = 1.07 for every 1% increase; 95% CI = 1.01-1.14); socio-economic level of the community (high and medium-high *vs.* low and medium-low OR = 2.16; 95% CI = 1.18-3.95); and having undergraduate training at the health care centre (OR = 0.44; 95% CI = 0.21-0.93). Nagelkerke's R squared was 0.11.

Factors related to professionals

A total of 597 professionals responded to the questionnaire, 254 (42.4%) cases and 343 (57.5%) controls. Refused to participate 96 professionals (62 cases and 34 controls). Participants were 298 nurses (52.4%), 191 doctors (33.6%), 58 paediatricians (10.2%), and 22 social workers (3.8%). Tables 2 and 3 show the results of the bivariate descriptive analysis. Table 2 shows that the variables related to involvement in community activities were sex, profession, training, self-confidence, and job satisfaction. Table 3 summarizes the responses of the professionals' opinions and attitudes to health promotion, community participation, and social sensitivity.

For peer review only - http://bmjopen.bmj.com/site/about/guidelines.xhtml

Table 2. Relationship of professional variables with performance of health-promoting community activities (CA).

Variables	Cases N (%)	Controls N (%)	Unadjusted OR (95% CI)*
**Sex**			
Male	41 (16.4)	84 (25.3)	Ref.
Female	209 (83.6)	248 (74.7)	**1.72 (1.13-2.61)**
**Profession**			
Nurse	156 (64.7)	142 (43.3)	Ref.
Doctor	40 (16.6)	151 (46)	**0.24 (0.16-0.36)**
Paediatrician	24 (10)	34 (10.4)	0.64 (0.36-1.3)
Social worker	21 (8.7)	1 (0.3)	**19.11 (2.53-143.9)**
**Specialized General practitioner**	22 (55)	91 (60.3)	0.81 (0.39-1.62)
**Tutor of Resident doctors**	10 (25)	37 (24.5)	1.02 (0.45-2.3)
**Nursing tutors**	93 (59.6)	58 (40.8)	**2.13 (1.34-3.39)**
**Work situation**			
Permanent	204 (80.3)	238 (70)	Ref.
Temporary contracts	40 (15.7)	74 (21.8)	**0.63 (0.41-0.96)**
Other	10 (3.9)	28 (8.2)	**0.41 (0.19-0.87)**
**Participation in research projects in last 5 years**	115 (47.7)	147 (43.9)	1.16 (0.83-1.62)*
**Specific training in CA**	142 (58.2)	104 (31.7)	**2.99 (2.12-4.23)***
**Job satisfaction**			
None/little	18 (7.1)	34 (10)	Ref.
Average	64 (25.3)	129 (38.1)	0.93 (0.49-1.78)
Quite a lot/a lot	171 (67.5)	176 (51.9)	1.83 (0.99-3.37)
**Working environment satisfaction**			
None/little	14 (5.6)	33 (9.8)	Ref.
Average	82 (32.5)	103 (30.5)	1.87 (0.94-3.73)
Quite a lot/a lot	156 (61.9)	202 (59.8)	1.82 (0.94-3.51)
**Self-confidence to carry out CA**			
None/little	31 (12.3)	72 (21.6)	Ref.
Average	81 (32.1)	154 (46.2)	1.22 (0.74-2.01)
Quite a lot/a lot	140 (55.5)	107 (32.1)	**3.03 (1.86-4.96)**
	**Median (p25-p75)**	**p***	
**Age**	49 (42-54)	48 (39-53)	**.048**
**Years qualified**	25 (18-31)	24 (15-29)	**.024**
**Years in primary care**	19 (11-23)	17 (10-23)	.552
**Years in current health centre**	11 (4-18)	7 (2-15)	**.000**
**Size of assigned quota**	1737 (1300-2043)	1600 (1300-1900)	**.026**
**Average daily consultations**	20 (15-30)	30 (20-35)	**.000**
**Average daily home visits**	1 (0.5-3)	1 (1-2)	.932
**Average consultation time**	10 (10-15)	10 (7-12)	**.000**
**Average home-visit time**	25 (20-30)	20 (15-30)	**.026**
**Biomedical practice style (+) Psychosocial (-)**	8 (6-9)	8 (7-9)	**.040**
**Political orientation: Left (-) right (+).**	2 (1-5)	3 (2-5)	**.026**

For dichotomous variables, the reference value is always absence of the variable. Data from Spain 2009.Bold-face: Chi-squared *p* ≤less than 0.05. * Mann-Whitney U test.

For peer review only - http://bmjopen.bmj.com/site/about/guidelines.xhtml

Table 3. Professional attitudes in cases and controls regarding health-promoting community activities (CA).

Variable	Cases N (%)	Controls N (%)	Unadjusted OR (IC 95%)
**Confidence in CA efficacy**			
Disagree/strongly disagree	9 (3.6)	19 (5.7)	Ref.
Agree	110 (43.8)	196 (59.0)	1.18 (0.51-2.71)
Strongly agree	132 (52.6)	117 (35.2)	**2.38 (1.03-5.46)**
**Motivated to carry out CA**			
Disagree- strongly disagree	44 (17.5)	149 (45.4)	Ref.
Agree	113 (45.0)	131 (39.9)	**2.92 (1.91-4.44)**
Strongly agree	94 (37.5)	48 (14.6)	**6.63 (4.08-10.7)**
**Citizen participation in health**			
Disagree/strongly disagree	23 (9.2)	46 (14.0)	Ref.
Agree	122 (49.0)	191 (58.1)	1.27 (0.73-2.21)
Strongly agree	104 (41.8)	92 (28.0)	**2.26 (1.27-4.01)**
**Citizen participation in health services**			
Disagree/strongly disagree	24 (9.6)	48 (14.5)	Ref.
Agree	109 (43.6)	198 (59.8)	1.10 (0.64-1.89)
Strongly agree	117 (46.8)	85 (25.7)	**2.75 (1.56-4.83)**
**Professionals' role in the community is well-defined**			
Disagree/strongly disagree	126 (51)	223 (66.8)	Ref.
Agree	103 (41.7)	97 (29)	**1.87 (1.32-2.67)**
Strongly agree	18 (7.3)	14 (4.2)	**2.27 (1.09-4.73)**
**Health education and community participation are closely linked**			
Disagree/strongly disagree	20 (8.1)	39 (11.7)	Ref.
Agree	128 (51.6)	194 (58.4)	1.28 (0.71-2.31)
Strongly disagree	100 (40.3)	99 (29.8)	**1.97 (1.07-3.61)**
**The health board drives CA**			
Disagree/strongly disagree	80 (33.8)	125 (39.8)	Ref.
Agree	123 (51.9)	163 (51.9)	1.17 (0.81-1.69)
Strongly agree	34 (14.3)	26 (8.3)	**2.04 (1.14-3.65)**
**Participates in activities in the neighbourhood where working**	138 (55.0)	83 (24.6)	**3.75 (2.64-5.33)**
**Collaborates with an NGO or civic entity**	151 (59.9)	133 (39.7)	**2.27 (1.62-3.17)**
**Freedom to organise timetable**	163 (65.7)	157 (47.7)	**2.10 (1.49-2.95)**
**Possibility colleagues will cover while performing CA**	99 (40.6)	79 (23.9)	**2.16 (1.51-3.11)**
**Can depend on colleagues to collaborate on CA**	215 (86.3)	222 (67.7)	**3.02 (1.96-4.63)**

Data from Spain 2009. Bold-face: Chi-squared $p \leq 0.05$

Due to missing data, 100 (16.8%) professionals were excluded from the multivariate analysis, leaving a total of 497. There were no significant differences between the excluded and included individuals. The final multivariate model (Table 4) shows that the following

For peer review only - http://bmjopen.bmj.com/site/about/guidelines.xhtml

factors were significantly associated with health-promoting community activities: profession (social workers do the most, followed by nurses and then physicians); specific training in community activities; motivation; social sensitivity (collaboration with NGOs and participation in neighbourhood activities); perception of support and coverage by colleagues while doing community activities; years working in the health care team; and being a nursing-student tutor.

Table 4. Multivariate model of performing health-promoting community activities (CA)

**Variable**	**General model (n=497) OR (95% CI)**
**Profession**	
Nurse	Ref.
Doctor	0.44 (0.24-0.82)
Paediatrician	1.55 (0.69-3.46)
Social worker	25.26 (2.53-251.9)
**Specific training in CA**	1.91 (1.17-3.11)
**Collaboration with NGO or civic entity**	1.91 (1.17-3.13)
**Possibility of work covered by colleagues during CA**	1.80 (1.08-2.99)
**Perception of support from colleagues in doing CA**	2.92 (1.49-5.73)
**Motivated to perform CA**	
Disagree or strongly disagree	Ref.
Agree	2.29 (1.28-4.08)
Strongly disagree	3.74 (1.85-7.54)
**Participates in activities in the local neighbourhood**	3.06 (1.86-5.05)
**Years at health centre**†	1.06 (1.03-1.09)
**Nursing-student tutor**	1.96 (1.09-3.53)
**Model Validity**	**Value**
**Chi^2**	221.338
**Nagelkerke R squared**	.483

† Continuous variable.

Discussion

Our study of the participation of health care workers in health-promoting activities in the community suggests that factors of the individual health care professional were more important than those of the health care team or the community. In particular, the specific

For peer review only - http://bmjopen.bmj.com/site/about/guidelines.xhtml

For peer review only

profession, attitudes, and opinions of an individual seemed to be crucial. This result confirms the frAC Project[28] hypothesis and those of other authors[20,27]. The final model of community and team variables had poor explanatory power (Nagelkerke R squared = 0.11). In contrast, the model of the characteristics of professionals had a considerably better explanatory power (Nagelkerke R squared = 0.43). Thus, at least for the variables selected in this study, individual characteristics are more important for the development of health-promoting community activities.

Our assessment of variables related with health care team participation in community activities suggests that team characteristics had greater impact than community characteristics. One exception was the socio-economic level of the community. This makes sense in the context of the Spanish health care system, which attempts to offer equitable care to people according to their needs and community activities are important tools to reduce inequalities in health care[34]. It should be noted that we found no relationship between the level of social interaction in the community and the implementation of community activities, in contrast with the findings of other authors[15-16].

Our results showing the important role of nurses in community activities are consistent with those of other authors[12-13,16,35] who highlighted the role of nurses in the development of community activities. This result is expected because nurses usually receive more training in community care. Another crucial group is social workers. Previous research reported the importance of social workers[12], but their role in the community varies in different regions of Spain. Their presence seems fundamental for development of health-promoting community activities in regions where they have significant roles in the community. In Spain, community work is generally not provided as part of primary health care; instead, it is relegated to professional volunteers or specific programs. Thus, our results recognize the importance of work by nurses and social workers and points to the possibility that their roles could be

For peer review only - http://bmjopen.bmj.com/site/about/guidelines.xhtml

modified or clarified. We also found that health centres that train undergraduate nurses have a lower level of community activities but that professional nurse-trainers have a greater involvement in community activities. This apparent contradiction may be resolved by considering two factors. First, the variable at the health centre level included training in various disciplines (from nursing to physiotherapy) without distinction, even though there is clearly difference among these in communitarian tradition. Second, the need for increased training would make it difficult for the health team to engage in community activities although, among the cases trainers carried out more activities than others. Health care workers who worked for more years at a centre were also more likely to perform community activities. This may be because medium- and long-term projects require continuity and stability, and it is more difficult for professionals who continually change centres or have temporary contracts to participate in community activities.

The pressures of working in health care and the heavy work burden were the main arguments offered by professionals when asked about their low participation in health promotion and prevention activities[18,21,23-26]. Our bivariate analysis supported this relationship, although the multivariate analysis did not. This implies that the professionals' perception was incorrect. In fact, the pressure of working in health care and the heavy work burden are not associated with low participation in community activities.

Our results indicated the importance of the opinions and attitudes of professionals when performing community activities, in accordance with other studies[14,20-21]. In fact, all variables regarding the opinions and attitudes of health care workers were significantly different between cases and controls. This suggests that individuals who perform community activities have similar attitudes: they employ a biopsychosocial[11,36] practice model, are more oriented towards health promotion, show recognition of the need for citizen participation in health services, and are more left-leaning in ideology. We also found that social sensitivity

For peer review only - http://bmjopen.bmj.com/site/about/guidelines.xhtml

was an important variable in the model for professionals. This variable measures involvement of the professional with the community beyond consultation work. It is not clear how some practices, responsibility for which is stipulated in primary care legislation and which should be similar for all professionals to assure fairness within the system, can be affected by a professional's personal discourse. Most health care professionals that we interviewed – both cases and controls -- said that their community roles were not well defined. This suggests that greater priority should be given to promote community activities in primary health care.

We found that having support within the health care team is associated with performing community activities. This finding should be highlighted, because all the professionals in this study were selected from the centres that engage in community activities so that, presumably, all cases can count on some support from their centres. It may be that some professionals were unaware of what their colleagues were doing, or that there were barriers to working with particular colleagues. Regardless of the explanation, this result suggests that application of the teamwork model[37], an assumed key feature of primary health care, has certain weaknesses.

Strengths and limitations

This study had an observational design, so we could not definitively establish causal relationships. Similarly, our definition of "community activities" may be a limitation because this concept can be defined or perceived in different ways; we made an attempt to control for this by use of a definition generated by expert consensus, which we then transformed into an algorithm. The questionnaires we used were *ad hoc* and some of the data regarding team variables, such as degree of social interaction in the community and the relationship between professionals, were provided by team managers. This may have led to a bias in information gathering due to a lack of knowledge or other unknown factors. Some variables that we did not examine could have explained participation in community activities, such as support from

For peer review only - http://bmjopen.bmj.com/site/about/guidelines.xhtml

primary care management, the health care administration and the centres' own coordinators or nursing managers. It would be valuable to assess the effect of these variables in future studies of the effect of team activities and priorities.

The main strengths of this study are that it examined a facet of health care that is relatively unexplored; the study subjects were from 5 different regions of Spain; the sample size was large; and it considered many distinct factors simultaneously. Furthermore, our design of the second study, in which cases and controls were matched by teams, adjusted for the effect of team and community variables, so a multilevel analysis was not necessary.

Implications for health care practice

Health care administrators who want to develop health-promotion activities for the community must provide visible and formal support for community initiatives, define better the expected communitarian tasks of each sanitarian professional, and provide job stability for all participating workers. Specific training is also important for the formation of health-promoting community activities. It may seem that attitudes, social sensitivity, or personal opinions are not modifiable; however, in our opinion, it is possible to modify these factors during undergraduate education. So, community perspective and their associated responsibility should be included in undergraduate sanitary formation.

Conclusions

A key responsibility of primary health care is provision of health-promoting community activities. We found that the actual implementation of these activities depends more on the characteristics of individual professionals than on the characteristics and composition of the health care team or the community. There were also significant differences among different types of professionals, and the contributions of nurses and social workers are fundamental. The pressures perceived by members of the health care team were not associated with involvement in community activities.

For peer review only - http://bmjopen.bmj.com/site/about/guidelines.xhtml

Acknowledgements

We want to express thanks to Magdalena Esteva for her patient reading of the manuscript.

Competing interests

All authors have completed the ICMJE uniform disclosure form at www.icmje.org/coi_disclosure.pdf and declare: no financial relationships with any organisations that might have an interest in the submitted work in the previous three years; no other relationships or activities that could appear to have influenced the submitted work

Funding

This study was supported by a grant from the Carlos III Health Institute (PI07/90383; PI07/90925; PI07/90636). It also received support from the Health Promotion and Preventive Activities in Primary Health Care Research Network (IAPP network RD 06/0018/), the ERDF funds, and the Institut de Salut de Palma (IDISPA). SM was contracted with the aid of a grant to stabilise employment in health research from the Carlos III Health Institute and benefited from a grant for further studies from the same institution. All institutions are public and did not have any role in study design, analysis, or publication.

Contributors

SM was the lead investigator, and was responsible for conception and design of the study, analysing data and writing the manuscript. **MR** has the original idea of the study. **SM, JR, JLRG, IMG, CBBA,** and **LES**, coordinated the field work in everyone of the regions involved. SM, JR, MJM, EZO, CBBA, LES, CV, MLLBA, TPJ, DMPM, MLLS, JLRG, RBV, CSV, MRB, MCV, MBC, ESF, MBD, IMG, BBR, AAL, SBC, ACT, BOB, RMB, PMP, MEO, MJPJ, MR contributed to the design and development of the study, have read the manuscript critically, made contributions and approved the final version.

Data Sharing Statement: Questionnaires and anonymised data are available on demand.

For peer review only - http://bmjopen.bmj.com/site/about/guidelines.xhtml

References

1. World Health Organisation: **Declaration of Alma Ata.** *WHO* 1978. [http://www.who.int/hpr/NPH/docs/declaration_almaata.pdf]

2. World Health Organisation: **The Ottawa Charter for Health Promotion.** *Health Promotion* 1986, **1**:i-v.

3. World Health Organisation: **Declarations of Jakarta** WHO 1997. [http://www.who.int/hpr/NPH/docs/jakarta_declaration_sp.pdf]

4. March S, Torres E, Ramos M. **Adult community health-promoting interventions in primary health care: A systematic review.** *Prev Med* 2015; 76 (2015) S94–S104

5. Ley General de Sanidad 14/1986, de 25 de abril. Boletín Oficial del Estado,nº102,(29/4/1986).

6. Real Decreto 1030/2006 del 15 de septiembre por el que se establece la cartera de servicios comunes del Sistema Nacional de Salud y el procedimiento para su actualización. *Boletín Oficial del Estado*,nº222,(16-9-2006).

7. Guldan GS: **Obstacles to community Health promotion**. *Soc sci med* 1996, **5**:689-695.

8. Irigoyen J: **La participación comunitaria en salud: Una reformulación necesaria.** In: *Nuevas perspectivas en Atención Primaria de Salud.* Edited by Aranda. Madrid: Díaz de Santos; 1994:183-221.

9. Gérvas J, Pérez-Fernández M, Palomo-Cobos L, Pastor-Sánchez R: **Veinte años de reforma de la Atención Primaria en España. Valoración para un aprendizaje por acierto/error.** Madrid: Ministerio Sanidad y Consumo 2005. [http://equipocesca.org/new/wp-content/uploads/2010/08/20-anos-de-reforma-de-la-ap-en-espana.pdf]

For peer review only - http://bmjopen.bmj.com/site/about/guidelines.xhtml

10. Regato P, Domínguez J, Sancho M: **Coordinación sanitaria y abordaje comunitario en atención primaria**. In: *La Atención Primaria de Salud en España y en sus comunidades autónomas.* Coordinated by Navarro V, Martín-Zurro A. Barcelona: Semfyc; 2009:149-168.

11. Turabián JL, Pérez B: **Grandes misterios. ¿Puedes ver al monstruo del lago Ness?: El modelo biopsicosocial y las actividades comunitarias.** *Aten Primaria* 2007, **39**:261-4.

12. March S, Soler M, Miller F, et al: **Variabilidad en la implantación de las actividades comunitarias de promoción de la salud en España.** *An Sist Sanit Navar.* In press.

13. Sáez S, Marqués F, Pallarés J, Serrano J, Orrio C, Martínez V: **Perfil de los profesionales de la salud de Lleida respecto a la educación para la salud (EpS).** *Aten Primaria* 1999, **24**:157-161.

14. Pérez-Franco B, Turabián JL, Magaña E, et al: **Factores asociados a la participación comunitaria en la atención primaria de la provincia de Toledo.** *Aten primaria* 1993, **12**: 339-344.

15. MJ Aguilar Idáñez: **La participación comunitaria en salud: ¿Mito o realidad?** Madrid: Díaz de Santos; 2001.

16. López E, Forcada C, Miller Frederick: **Factores que facilitan y dificultan el desarrollo de los proyectos comunitarios. Estudio observacional de la red AUPA de centros de atención primaria de Cataluña.** *Aten primaria* 2010, **42**:218-25.

17. Duro JC: **Actitudes de los profesionales de atención primaria de Madrid sobre los grupos.** *Aten Primaria* 2005, **36**:71-7.

18. Díaz G: **Factores relacionados con la práctica de educación sanitaria por los médicos de Atención primaria de Galicia.** *Rev Esp Salud Pública* 2001, **75**:529-540.

For peer review only - http://bmjopen.bmj.com/site/about/guidelines.xhtml

19. Maheux B Dufort, F Lambert J et al: **The professional attitudes and clinical practices of men and women generalists.** *Can Fam Physician* 1989, **35**:59-63.

20. Maheux B, Pineault R, Lambert J et al: **Factors influencing physicians' preventive practices.** *Am J Prev Med* 1989, **5**:201-206.

21. Myrand A, Beehler G, Kuo C, Mahoney M: **Explaining the de-prioritization of primary prevention: Physicians' perceptions of their role in the delivery of primary care.** *BMC Public Health* 2003, **3**:15. doi:10.1186/1471-2458-3-15.

22. Walter U, Flick U, Neuber A, et al: **Putting prevention into practice: qualitative study of factors that inhibit and promote preventive care by general practitioners, with a focus on elderly patients.** *BMC Family practice* 2010, **11**:68. doi:10.1186/1471-2296-11-68

23. Puig ribera A, McKenna J, Riddoch C: **Attitudes and practices of physicians and nurses regarding physical activity promotion in the Catalan primary health-care system.** *Europ J Public Health* 2005, **15**:569–75.

24. Wilhemsson S, Lindberg L: **Health promotion: Facilitators and barriers perceived by district nurses.** *Int J Nurs Practice* 2009, **15**:156-163.

25. Rubio-Valera M, Pons-Vigues M, Martinez-Andres M et al. **Barriers and Facilitators for the implementation of primary prevention and health promotion activities in primary care: A Syntethesis through meta-etnography.** Plos one 2014;9:e89554. Doi: 10.1371/journal.pone.0089554.

26. Badertscher N, Rossi PO, Rieder A et al: **Attitudes, barriers and facilitators for health promotion in the elderly in primary care.** *Swiss Med Wkly* 2012;**142**:w13606.

27. Maheux B, Pineault L, Béland F et al: **Factors influencing physicians' orientation toward prevention.** *Am J Prev Med.* 1987, **3**:12-8.

For peer review only - http://bmjopen.bmj.com/site/about/guidelines.xhtml

28. March S, Ripoll J,Ruiz-Giménez JL, et al: **Observational study on factors related to health-promoting community activity development in primary care (frAC Project): a study protocol.** *BMJ Open* 2012, **2**. e001287 doi:10.1136/bmjopen-2012-001287.

29. March S, Jordán M, Montaner I et al. **¿Qué hacemos en el barrio? Descripción de las actividades comunitarias de promoción de la salud en atención primaria: Proyecto frAC.** Gac Sanit 2014; **28**:267-73.

30. March S, Ramos M, Soler M, Ruiz-JiménezJL, Miller F, Domínguez J, et al: **Revisión documental de experiencias de actividad comunitaria en atención primaria de salud.** *Aten Primaria* 2011, **43**:289-296.

31. T Martínez-cañavate: **Modelo multinivel explicativo de la utilización de las consultas de atención primaria en Andalucía**. *Phd tesis.* Granada university, faculty of medicine; 2007. [http://hera.ugr.es/tesisugr/16905234.pdf]

32. Mira JJ, Llinás G, Gil V, Orozco D, Palazón I, Vitaller J: **Validación de un instrumento para identificar estilos de práctica profesional del médico de atención primaria.** *Aten Primaria* 1998, **21**:14-22.

33. Rey del Castillo P: **Nota metodológica sobre los indicadores del barómetro.** *Rev Española de Investigaciones Sociológicas* 2004, **108**:151-78.

34. Comisión para reducir las desigualdades en salud en España: **Propuesta de políticas e intervenciones para reducir las desigualdades en salud en Espanya**. *Gac Sanit* 2012. doi:10.1016/j.gaceta.2011.07.024.

35. Hogg W, Dahrouge S. Russell G et al: **Health promotion activity in primary care: performance of models and associated factors.** *Open medicine* 2008, **3**:165-73.

36. Engel GL: **The need for a new medical model: a challenge to biomedicine.** Science 1977, **196**:129–36.

For peer review only - http://bmjopen.bmj.com/site/about/guidelines.xhtml

37. Grumbach K, Bodenheimer T: **Can Health Care teams improve Primary Care practice?** JAMA 2004, **291**:1246-51.

For peer review only

For peer review only - http://bmjopen.bmj.com/site/about/guidelines.xhtml

BMJ Open

Factors related to the development of health-promoting community activities in Spanish primary health care: Two case-control studies

Journal:	*BMJ Open*
Manuscript ID	bmjopen-2017-015934.R1
Article Type:	Research
Date Submitted by the Author:	24-Apr-2017
Complete List of Authors:	March, Sebastià; Baleares Health services-IbSalut, Primary Care Research Unit of Mallorca; Instituto de investigación en salud de Palma (IDISPA)<br>Ripoll, Joana; Baleares Health services-IbSalut, Primary Care Research Unit of Mallorca; Baleares Health services-IbSalut, Primary Care Research Unit of Mallorca<br>Jordan Martin, Matilde Jordan Martin; Estrecho de Correa Primary health center, Madrid Health Services<br>Zabaleta del Olmo, Nieves; Fundacio Jordi Gol i Gurina, IDIAP<br>Benedé Azagra, Carmen Belén; Aragon Health Services, Oliver Primary health center<br>Elizalde Soto, Lázaro; Gobierno de Navarra Departamento de Salud, public health institute; Public Health and epidemiology CIBER,<br>Vidal, Mª Clara; Baleares Health services-IbSalut, Primary Care Research Unit of Mallorca; Instituto de investigación en salud de Palma (IDISPA)<br>Bauzà Amengual, M. de Lluc; Balearic Islands University,<br>Planas Juan, Trinidad; Baleares Health services-IbSalut, Son Gotleu Primary health care center<br>Maria Pèrez, Damiana Maria Pèrez; Baleares Health services-IbSalut, Son Gotleu Primary health care center<br>Llull Sarralde, Micaela; Baleares Health services-IbSalut, San Agustí Primary health care center<br>Ruiz-Giménez, Juan Luís; Madrid Health Services, Madrid, Vicente Soldevilla Primary health care center<br>Bajo Viñas, Rosa; Madrid Health care services, Loeches Primary health care center<br>Solano Villarubia, Carmen Solano Villarubia; madrid Health care services, DA Suroeste Health center<br>Rodriguez Bajo, Maria<br>Cordoba Victoria, Manuela Cordoba Victoria; SOMAMFyC<br>Montaner-Gomis, Isabel; Institut Catala De La Salut, El Carmel Health center<br>Badia Capdevila, Marta; Institut Catala De La Salut, El carmel Health center<br>Serrano Ferrandez, Elena; Institut Catala De La Salut, El carmel health center<br>Bosom Diumenjo, Maria; Institut Catala De La Salut, Sant Rafel Primary Health Center<br>Bolibar-Ribas, Buenaventura; Fundacio Jordi Gol i Gurina, IDIAP<br>Antoñanzas Lombarte, Angel; Aragon Health services, Delicias del Sur health center

Bregel Cotaina, Samantha; Instituto Aragones de Ciencias de la Salud, Primary health care research unit
Calvo Tocado, Ana; Zaragoza Public Health provincial subdirection
Olivan Blázquez, Barbara; Instituto Aragones de Ciencias de la Salud, primary health care research unit
Magallon Botaya, Rosa; Aragon Health services, Arrabal health center
Marín Palacios, Pilar Marín Palacios; Gobierno de Navarra Departamento de Salud, Public health institute
Echauri Ozcoidi, Margarita; Gobierno de Navarra Departamento de Salud, public health institute
Perez-Jarauta, Mª Jose Perez-Jarauta; Gobierno de Navarra Departamento de Salud, public health institute
Llobera, Joan; Baleares Health services-IbSalut, 1. Primary Care Research Unit of Mallorca; Instituto de investigación en salud de Palma (IDISPA)
Ramos, Maria; Govern de les Illes Balears Conselleria de Salut Familia i Benestar Social, Direcció General de salut Pública i Participació; Instituto de investigación en salud de Palma (IDISPA)

**Primary Subject Heading**:	General practice / Family practice
Secondary Subject Heading:	Health services research, Public health
Keywords:	Health promotion, Primary health care, Health services research, Community health, Health team, Health professionals

SCHOLARONE™ Manuscripts

For peer review only

Factors related to the development of health-promoting community activities in Spanish primary health care: Two case-control studies

Sebastià March[1], Joana Ripoll[1], Matilde Jordan Martin[2], Edurne Zabaleta del Olmo[3], Carmen Belén Benedé Azagra[4], Lázaro Elizalde Soto[5], Mª Clara Vidal[1], María de Lluc Bauzà Amengual[6], Trinidad Planas Juan[7], Damiana Maria Pérez Mariano[7], Micaela Llull Sarralde[8], Juan Luís Ruiz-Giménez[9], Rosa Bajo Viñas[10] , Carmen Solano Villarubia[11], Maria Rodriguez Bajo[12], Manuela Cordoba Victoria[13], Marta Badia Capdevila[3], Elena Serrano Ferrandez[3], Maria Bosom Diumenjo[14], Isabel Montaner Gomis[15], Bonaventura Bolívar Ribas[3], Angel Antoñanzas Lombarte[16], Samantha Bregel Cotaina[17], Ana Calvo Tocado[18], Barbara Olivan Blázquez[17], Rosa Magallón Botaya[19], Pilar Marín Palacios[5], Margarita Echauri Ozcoidi[5], María Jose Perez-Jarauta[5], Joan Llobera[1], Maria Ramos[20].

1. Primary Care Research Unit of Mallorca, Baleares Health Services-IbSalut, Palma de Mallorca, Spain.
2. Estrecho de Correa Primary Health Center, Madrid Health Services, Madrid, Spain.
3. Primary Care Health Research Institute IDIAP Jordi Gol, Barcelona, Spain.
4. Oliver Primary Health Center, Aragon Health Services, Zaragoza, Spain.
5. Navarra Public Health Institute, and Public Health and Epidemiology CIBER, Pamplona, Spain.
6. Balearic Islands University, Palma de Mallorca, Spain.
7. Son Gotleu Primary Health Center, Baleares Health Services-IbSalut, Palma de Mallorca, Spain.
8. San Agustí Primary Health Center, Baleares Health Services-IbSalut, Palma de Mallorca, Spain.
9. Vicente Soldevilla Primary Health Center, Madrid Health Services, Madrid, Spain.
10. Loeches Primary Health Center, Madrid Health Services, Madrid, Spain.
11. DA Sureste Primary Health Center, Madrid Health Services, Madrid, Spain.
12. Freelance, Madrid, Spain
13. SOMAMFYC, Madrid, Spain
14. Sant Rafel Primary Health Center, Catalonian Health Services Cat-Salut, Barcelona, Spain.
15. El Carmel Primary Health Center, Catalonian Health Services Cat-Salut, Barcelona, Spain.
16. Delicias del Sur Primary Health Center, Aragon Health Services, Zaragoza, Spain.
17. Primary Care Research Unit, Aragon, Spain
18. Zaragoza Public Health department, Zaragoza, Spain.
19. Arrabal Primary Health Center, Aragon Health Services, Zaragoza, Spain.
20. Public Health Department, Balearic Islands Health Department, Spain.

Corresponding author:
Sebastià March
E-mail: smarch@ibsalut.caib.es,

For peer review only - http://bmjopen.bmj.com/site/about/guidelines.xhtml

Abstract

Objective: Spanish primary health care teams have the responsibility of performing health-promoting community activities, although such activities are not widespread. Our aim was to identify the factors related to participation in those activities.

Design: Two case-control studies. **Setting:** Performed in primary care of 5 Spanish regions. **Subjects:** In the first study, cases were teams that performed health promoting community activities and controls were those that did not. In the second study (on case teams from the first study), cases were professionals who developed these activities and controls were those who did not. **Main outcome measures:** Team, professional, and community characteristics collected through questionnaires (team managers/professionals) and from secondary sources.

Results: The first study examined 203 teams (103 cases, 100 controls). Adjusted factors associated with performing community activities were percentage of nurses (OR=1.07, 95%CI =1.01-1.14), community socio-economic status (higher *vs.* lower OR =2.16, 95%CI=1.18-3.95), and performing undergraduate training (OR=0.44, 95%CI=0.21-0.93). In the second study, 597 professionals responded (254 cases, 343 controls). Adjusted factors were professional classification (physicians do fewer activities than nurses and social workers do more), training in community activities (OR=1.9, 95%CI =1.2-3.1), team support (OR=2.9, 95%CI =1.5-5.7), seniority (OR=1.06, 95%CI=1.03-1.09), nursing tutor (OR =2.0, 95%CI =1.1-3.5), motivation (OR =3.7, 95%CI=1.8-7.5), collaboration with non-governmental organizations (OR=1.9, 95%CI=1.2-3.1), and participation in neighbourhood activities (OR=3.1, 95%CI=1.9-5.1).

Conclusions: Professional characteristics seem to have a greater influence than team/community factors in performing health-promoting community activities. In contrast to

For peer review only - http://bmjopen.bmj.com/site/about/guidelines.xhtml

the opinion expressed by many professionals, workload is not related to performance of health-promoting community activities.

Keywords: Health promotion, Primary health care, Health services research, Community health, Health team, health professionals

Strengths and limitations of this study

- This study examined a topic relatively unexplored, as community health promotion by primary health care.

- A case-control approach was chosen, where cases were subjects (professionals or teams) who were developing community activities, and controls those who not.

- Design allows to explore many distinct factors simultaneously, from different sources, and in two levels: Health team and professional.

- An operative definition of community activities was used, which recognizes the heterogeneity of health promotion interventions developed with the community in Spanish primary health care.

- Because of its observational design, there are limitations to establishing causal inferences.

For peer review only

Introduction

The Ottawa Charter for Health Promotion[1] defined Health promotion as the process of enabling people to increase control over, and to improve, their health. This definition implies the importance of empowering people[2] to take control over what determines their health. One tool to work in this direction are health promoting community activities. These can come in a variety of forms, from walking groups in a neighbourhood to holding meetings with local authorities, schools or civil associations to develop specific health programs. Experts at numerous international conferences have agreed on the importance of health-promoting community activities for improving public health[1,3-4], and there is some evidence that these activities are effective[5].

Since the implementation of health care reform in Spain during the 1980's[6-7], primary health care teams have had responsibility for carrying out health promotion activities. These teams, located in health care centres throughout the country, are composed of physicians, community nurses, paediatricians, midwives, social workers, and other health care professionals (gynaecologists, psychologists, and psychiatrists) in a system that offers almost-universal and free health care. These conditions are ideal for an interdisciplinary approach to encourage health promoting activities. However, as in other countries[8], implementation of these interventions differs greatly among the different health care teams[9-11], and relatively few professionals are involved[11-13].

There has been limited studies of the factors related to the development of health-promoting interventions in the community. These factors may be broadly classified as related to the community, to the team, or to the individual professionals. With regard to community factors, some studies indicated that the rural/urban environment[14], size of the municipality[15], level of social interaction within the community[15-17], and presence of a focal point for participation in community health initiatives[15-16] influence the practice of health

For peer review only - http://bmjopen.bmj.com/site/about/guidelines.xhtml

promoting community activities. The primary health care team factors include job satisfaction[18], management of time and setting within the center[14], composition and organization of the team[15-16], years of team operation[15], intra-team support[17], and work burden[8,19]. Studies of professionals have highlighted sex[19-21], age[15,20], professional status[14], specific training[22-25], motivation[17-18], and the model applied (biomedical or psychosocial)[18]. The attitudes and beliefs of the professionals, such as trust in the effectiveness of preventive actions[18,22,25-27], self-reported efficacy in carrying out activities[8,22-23], or to support community participation[15,21] are also important. An additional element is professionals' social sensitivity, as evaluated through their personal political leanings[28]. According to professionals themselves, the most important factor is the lack of time available to assume the care burden[19,22,24-27]. All these features have been observed separately, mostly in descriptive studies.

This study presents some of the results of the Factors Related to Health-Promoting Community Activity Development in Primary Care (frAC) Project, whose complete methodology and aims were described elsewhere[29]. The purpose of this study is to identify primary health care team and community factors related to implementation of health promoting community activities in primary care and factors that may explain why only some professionals within a team participate in those community activities.

Methods

Two case-control studies were performed. In the first, the cases were the teams that performed health promoting community activities and the controls were those that did not. The second study examined only teams that implemented these community activities to adjust for community/team factors; in this second analysis the cases were professionals who developed these activities and the controls were those who did not. The study was set in

For peer review only - http://bmjopen.bmj.com/site/about/guidelines.xhtml

primary health care centers in five regions of Spain (Balearic Islands, Catalonia, Aragon, Madrid, and Navarra). Data collection was carried out between 2009-2010.

Selection of cases and controls

We used a conceptual definition of health promoting community activity reached in a previous study [30-31]. This was converted into an algorithm for use in this study[29]: this activity had to be a non-isolated activity carried out in the previous year, in which the professional participated on behalf of the health centre. Furthermore, it had to have involvement and active participation of the community, or the population had to have the capacity to influence the intervention, or it was a cross-sector activity involving collaboration with entities outside of health care (*e.g.* education, social services). Health preventive interventions which does not have a health promotion approach were excluded.

We telephoned all the health centres in the research areas asking if they had participated in health promoting community activities during the last year. If they did, we contacted a participant of the activity and gave him/her a questionnaire to confirm that the activity met our inclusion criteria. For the first study, health teams that participated in at least one community activity in the last year were selected as cases, and controls were those who had not participated. For the second study, we selected all professionals who participated in the implementation of those community activities as cases and professionals who did not participate as controls. In every health team we selected at least one control for every case.

Variables

Information was gathered through three distinct questionnaires (available on request) that were given to the community, the team, and the professionals[29]. Description of health promoting community activities are described elsewhere[30].

The data collected from the community (from secondary sources and from questionnaires to centre managers) were: demographic composition; socio-economic level; degree of social

For peer review only

interaction; health centre setting; geographical dispersion based on an ordinal variable used by the National Health System for resource allocation (from G1 [less dispersed] to G4 [more dispersed]); and existence of health boards (community participation agencies described in health legislation)[16].

The data collected from the team (collected from secondary sources and questionnaires to centre managers) were: percentages of different professionals (nurses, physicians, and paediatricians); year of opening; population of service area; professional/population and doctor/nurse ratios; availability of a space in the centre to carry out group activities and presence of a team member responsible for health education; collaboration with the area health board where applicable; training of resident physicians; nurses and nursing or physiotherapy students; evaluation of professional relationships between distinct professional disciplines (based on a Likert-type scale ranging from 1 [poor] to 5 [excellent]).

The data collected from the professionals (from individual self-administrated questionnaires) were: sex; age; profession; working situation; health care burden; size of quota allocated; average number and duration of consultations per day at the centre or at home; tutoring; participation in research or specific training in health promotion in the previous five years; years since graduation; years worked in primary care and as a member of the current health care team; autonomy to organize scheduling; arrangements with colleagues to cover health care tasks while carrying out community activities; support of fellow team members in conducting these activities; working atmosphere; job satisfaction; and self-assessed efficacy in conducting community work (based on a Likert-type scale ranging from 1 [poor] to 5 [excellent]).

Opinions and attitudes were measured by responses to various statements developed by the research team, using a Likert-type scale with four options ranging from "strongly

For peer review only - http://bmjopen.bmj.com/site/about/guidelines.xhtml

agree" to "strongly disagree". The statements dealt with trust in the effectiveness of the health promotion community activities, motivation to carry out these activities, citizen participation in health care decision-making, need to strengthen agencies involved in citizen participation in health care, responsibility for primary care in the community, definition of professional role, link between health education and community participation, and the effectiveness of the boards in driving community activities. A four-item validated scale was used to evaluate each professional's clinical practice style (biomedical-psychosocial)[32-33] by assessing the attitude towards the demands of psychosocial and clinical care at work. Finally, social sensitivity data were collected through proxy variables: participation during the previous twelve months in activities in the neighbourhood where the professional works; involvement in any NGO or civil association; and political tendency (assessed through a visual scale adapted from the Centre for Sociological Research[34] Spanish monthly barometer).

Calculation of sample size

For the first study, we calculated that at least 91 teams would be needed in each group based on an OR of 2.5, an alpha risk of 0.05, a beta of 0.2, and an expected proportion of 0.5 in the controls. For the second study, we calculated that at least 222 professionals would be needed in each group based on an OR of 1.75, an alpha risk of 0.05, a beta of 0.2, and an expected proportion of 0.5 in the controls.

Data analysis and management

A descriptive analysis of all variables was carried out to compare their distributions in each group. Categorical variables are expressed as percentages and continuous variables as means with 95% confidence intervals (95% CIs) or as medians and percentiles depending on the distribution. Main variable was, for both studies, to be case or control. The relationship with the main variable was assessed using the chi-squared test for categorical variables and Student's *t*-test and the Mann-Whitney U non-parametric test for continuous

For peer review only - http://bmjopen.bmj.com/site/about/guidelines.xhtml

variables. The strength of an association was expressed through unadjusted OR and its 95% CI.

We also performed a logistic regression analysis to calculate adjusted ORs. We constructed a first model using each variable whose *p*-value was less than 0.2 in the bivariate analysis. From this model, we tested and compared variations using the maximum likelihood method, eliminating variables that had *p*-values less than 0.05 in the Wald test. The collinearity of the variables was examined and interactions tested. We established a different model for the health care teams and for the professionals. All analyses were conducted using SPSS ver. 14 and Epidat ver. 3.1 software.

Ethical aspects

The study was approved by the Research Commissions in all the participant regions and by the Research Ethics Committee in Madrid (Area 1) and the University Research Institute in Primary Care (IDIAP) Jordi Gol in Barcelona. The questionnaire was completed anonymously by the professionals, with the identity of individuals and centres coded to ensure blinding of the researchers.

Results

Factors related to the health care team

We examined 203 health care teams (103 cases [50.7%] and 100 controls) in the first study. Table 1 shows the results of the initial bivariate analysis. The variables significantly related to team involvement in community activities were the socioeconomic level of the community, collaboration on training in physiotherapy or nursing, participation in a health board, percentage of nurses in the team, patient/physician ratio, and patient/nurse ratio.

For peer review only - http://bmjopen.bmj.com/site/about/guidelines.xhtml

Table 1. Relationship of community and primary health care team (PHT) characteristics with performance of health-promoting activities in the community.

Variable	PHT cases N (%)	PHT controls N (%)	Unadjusted OR (95% CI)
**Health Care setting**			
Urban	73 (71.6)	68 (68.0)	Ref.
Rural	29 (28.4)	32 (32.0)	0.84 (0.46-1.54)
**Socioeconomic level**			
High and medium-high	52 (51)	65 (65)	1
Medium-low and low	50 (49)	35 (35)	**1.79 (1.01-3.14)**
**Degree of social interaction**			
High	22 (23.9)	23 (26.1)	Ref.
Medium	43 (46.7)	35 (39.8)	1.28 (0.61-2.68)
Low	27 (29.3)	30 (34.1)	0.94 (0.43-2.06)
**Population of municipality**			
>1 million	47 (45.6)	48(48)	Ref.
500,000-1 million	7 (12.6)	7 (7)	1.90 (0.70-5.18)
10,000-500,000	22 (21.4)	33 (33)	0.68 (0.35-1.33)
<10,000	21 (20.4)	12 (12)	1.79 (0.79-4.04)
**Degree of geographical dispersion**			
G1 (less dispersed)	62 (66.7)	60 (63.8)	Ref.
G2	11 (11.8)	17 (18.1)	0.63 (0.27-1.45)
G3	9 (9.7)	11 (11.7)	0.79 (0.31-2.05)
G4 (more dispersed)	11 (11.8)	6(6.4)	1.77 (0.67-5.10)
**Active health board**	67 (66.3)	60 (61.9)	1.21 (0.68-2.17)
**Training centre**			
Resident Doctors	35 (34.7)	34 (34.3)	1.01 (0.56-1.82)
Resident Nurses	23 (23.5)	29 (30.2)	0.71 (0.37-1.34)
Nursing/physiotherapy students	73 (72.3)	84 (85.7)	**0.43 (0.21-0.89)**
**Space for group activities**	83 (81.4)	79 (79)	1.16 (0.58-2.32)
**Health education manager**	45 (44.1)	46 (46)	0.93 (0.53-1.61)
**Participation in health board**	56 (83.6)	38 (63.3)	**2.95 (1.28-6.78)**
**Continuous variables**	**Mean (95% CI)**		**p***
PHT operational years	14.3 (13-15.2)	12.9 (11.7-14.1)	.075
Total No. professionals	36.4 (33.1-39.6)	37.5 (34-41.1)	.486
% nurses	35.4 (34.5-36.4)	33.5 (32.4-34.5)	**.010**
% paediatricians	7.0 (6.3-7.7)	7.4 (6.7-8.1)	.450
Population covered by PHT	20031 (18059-22002)	21770.4 (19530-24010)	.247
	**Median (IQR)**		**p****
% doctors	33.3 (29.5-38.3)	33.3 (29.9-36.4)	.532
% non-EU immigrants	11.1 (5.8-16.2)	11.1 (8.6-15.7)	.690
% population <15 years	13.0 (11.6-14.7)	12.9 (11.3-14.4)	.352
% population >65 years	17.8 (15.0-21.8)	18.9 (13.8-21.5)	.976
% population women	51 (49.5-53)	51.2 (50.3-53.1)	.403
Population ratio registered/professional	524 (444-658)	578 (482-669)	.122
Population ratio registered/doctor	1659 (1454-1905)	1734 (1533-2040)	**.034**
Population ratio registered/nurse	1508 (1284-1847)	1723 (1434-2057)	**.008**
Doctor/nurse ratio	.94 (.86-1.09)	1 (.89-1.14)	.085
**Professional relationships**			
Doctor::nurse	4 (3-4)	4 (3-4)	.376
Doctor::social worker	4 (3-5)	4 (3-4)	.978
Nurse::social worker	4 (3-5)	4 (3-4)	.845

Data from Spain 2009. For dichotomous variables, the reference value is always absence of the variable.* Student's t-test P. ** Mann-Whitney U test P. Bold-face: $p \leq 0.05$.

For peer review only

Eleven teams (5.0%) were excluded from the logistic regression analysis due to missing data. The 3 variables remaining in the final model were percentage of nurses in the team (OR = 1.07 for every 1% increase; 95% CI = 1.01-1.14); socio-economic level of the community (high and medium-high *vs.* low and medium-low OR = 2.16; 95% CI = 1.18-3.95); and having undergraduate training at the health care centre (OR = 0.44; 95% CI = 0.21-0.93). Nagelkerke's R squared was 0.11.

Factors related to professionals

A total of 597 professionals responded to the questionnaire, 254 (42.4%) cases and 343 (57.5%) controls. Refused to participate 96 professionals (62 cases and 34 controls). Participants were 298 nurses (52.4%), 191 doctors (33.6%), 58 paediatricians (10.2%), and 22 social workers (3.8%). Tables 2 and 3 show the results of the bivariate descriptive analysis. Table 2 shows that the variables related to involvement in health promoting community activities were sex, profession, training, self-confidence, and work situation. Also years of experience, age, workload, practice style and political orientation seems to be related. Table 3 summarizes the responses of the professionals' opinions and attitudes to health promotion, community participation, and social sensitivity.

For peer review only

For peer review only - http://bmjopen.bmj.com/site/about/guidelines.xhtml

Table 2. Relationship of professional variables with performance of health-promoting community activities (CA).

Variables	Cases N (%)	Controls N (%)	Unadjusted OR (95% CI)*
**Sex**			
Male	41 (16.4)	84 (25.3)	Ref.
Female	209 (83.6)	248 (74.7)	**1.72 (1.13-2.61)**
**Profession**			
Nurse	156 (64.7)	142 (43.3)	Ref.
Doctor	40 (16.6)	151 (46)	**0.24 (0.16-0.36)**
Paediatrician	24 (10)	34 (10.4)	0.64 (0.36-1.3)
Social worker	21 (8.7)	1 (0.3)	**19.11 (2.53-143.9)**
**Specialized General practitioner**	22 (55)	91 (60.3)	0.81 (0.39-1.62)
**Tutor of Resident doctors**	10 (25)	37 (24.5)	1.02 (0.45-2.3)
**Nursing tutors**	93 (59.6)	58 (40.8)	**2.13 (1.34-3.39)**
**Work situation**			
Permanent	204 (80.3)	238 (70)	Ref.
Temporary contracts	40 (15.7)	74 (21.8)	**0.63 (0.41-0.96)**
Other	10 (3.9)	28 (8.2)	**0.41 (0.19-0.87)**
**Participation in research projects in last 5 years**	115 (47.7)	147 (43.9)	1.16 (0.83-1.62)
**Specific training in CA**	142 (58.2)	104 (31.7)	**2.99 (2.12-4.23)**
**Job satisfaction**			
None/little	18 (7.1)	34 (10)	Ref.
Average	64 (25.3)	129 (38.1)	0.93 (0.49-1.78)
Quite a lot/a lot	171 (67.5)	176 (51.9)	1.83 (0.99-3.37)
**Working environment satisfaction**			
None/little	14 (5.6)	33 (9.8)	Ref.
Average	82 (32.5)	103 (30.5)	1.87 (0.94-3.73)
Quite a lot/a lot	156 (61.9)	202 (59.8)	1.82 (0.94-3.51)
**Self-confidence to carry out CA**			
None/little	31 (12.3)	72 (21.6)	Ref.
Average	81 (32.1)	154 (46.2)	1.22 (0.74-2.01)
Quite a lot/a lot	140 (55.5)	107 (32.1)	**3.03 (1.86-4.96)**
	**Median (p25-p75)**	**p***	
**Age**	49 (42-54)	48 (39-53)	**.048**
**Years qualified**	25 (18-31)	24 (15-29)	**.024**
**Years in primary care**	19 (11-23)	17 (10-23)	.552
**Years in current health centre**	11 (4-18)	7 (2-15)	**.000**
**Size of assigned quota**	1737 (1300-2043)	1600 (1300-1900)	**.026**
**Average daily consultations**	20 (15-30)	30 (20-35)	**.000**
**Average daily home visits**	1 (0.5-3)	1 (1-2)	.932
**Average consultation time**	10 (10-15)	10 (7-12)	**.000**
**Average home-visit time**	25 (20-30)	20 (15-30)	**.026**
**Biomedical practice style (+) Psychosocial (-)**	8 (6-9)	8 (7-9)	**.040**
**Political orientation: Left (-) right (+).**	2 (1-5)	3 (2-5)	**.026**

Data from Spain 2009. For dichotomous variables, the reference value is always absence of the variable. Bold-face: Chi-squared *p* ≤less than 0.05. * Mann-Whitney U test.

For peer review only

For peer review only - http://bmjopen.bmj.com/site/about/guidelines.xhtml

Table 3. Professional attitudes in cases and controls regarding health-promoting community activities (CA).

Variable	Cases N (%)	Controls N (%)	Unadjusted OR (IC 95%)
**Confidence in CA efficacy**			
Disagree/strongly disagree	9 (3.6)	19 (5.7)	Ref.
Agree	110 (43.8)	196 (59.0)	1.18 (0.51-2.71)
Strongly agree	132 (52.6)	117 (35.2)	**2.38 (1.03-5.46)**
**Motivated to carry out CA**			
Disagree- strongly disagree	44 (17.5)	149 (45.4)	Ref.
Agree	113 (45.0)	131 (39.9)	**2.92 (1.91-4.44)**
Strongly agree	94 (37.5)	48 (14.6)	**6.63 (4.08-10.7)**
**Citizen participation in health**			
Disagree/strongly disagree	23 (9.2)	46 (14.0)	Ref.
Agree	122 (49.0)	191 (58.1)	1.27 (0.73-2.21)
Strongly agree	104 (41.8)	92 (28.0)	**2.26 (1.27-4.01)**
**Citizen participation in health services**			
Disagree/strongly disagree	24 (9.6)	48 (14.5)	Ref.
Agree	109 (43.6)	198 (59.8)	1.10 (0.64-1.89)
Strongly agree	117 (46.8)	85 (25.7)	**2.75 (1.56-4.83)**
**Professionals' role in the community is well-defined**			
Disagree/strongly disagree	126 (51)	223 (66.8)	Ref.
Agree	103 (41.7)	97 (29)	**1.87 (1.32-2.67)**
Strongly agree	18 (7.3)	14 (4.2)	**2.27 (1.09-4.73)**
**Health education and community participation are closely linked**			
Disagree/strongly disagree	20 (8.1)	39 (11.7)	Ref.
Agree	128 (51.6)	194 (58.4)	1.28 (0.71-2.31)
Strongly disagree	100 (40.3)	99 (29.8)	**1.97 (1.07-3.61)**
**The health board drives CA**			
Disagree/strongly disagree	80 (33.8)	125 (39.8)	Ref.
Agree	123 (51.9)	163 (51.9)	1.17 (0.81-1.69)
Strongly agree	34 (14.3)	26 (8.3)	**2.04 (1.14-3.65)**
**Participates in activities in the neighbourhood where working**	138 (55.0)	83 (24.6)	**3.75 (2.64-5.33)**
**Collaborates with an NGO or civic entity**	151 (59.9)	133 (39.7)	**2.27 (1.62-3.17)**
**Freedom to organise timetable**	163 (65.7)	157 (47.7)	**2.10 (1.49-2.95)**
**Possibility colleagues will cover while performing CA**	99 (40.6)	79 (23.9)	**2.16 (1.51-3.11)**
**Can depend on colleagues to collaborate on CA**	215 (86.3)	222 (67.7)	**3.02 (1.96-4.63)**

Data from Spain 2009. Bold-face: Chi-squared $p \leq 0.05$

Due to missing data, 100 (16.8%) professionals were excluded from the multivariate analysis, leaving a total of 497. There were no significant differences between the excluded and included individuals. The final multivariate model (Table 4) shows that the following

For peer review only - http://bmjopen.bmj.com/site/about/guidelines.xhtml

factors were significantly associated with health-promoting community activities: profession (social workers do the most, followed by nurses and then physicians); specific training in community activities; motivation; social sensitivity (collaboration with NGOs and participation in neighbourhood activities); perception of support and coverage by colleagues while doing community activities; years working in the health care team; and being a nursing-student tutor.

For peer review only

For peer review only - http://bmjopen.bmj.com/site/about/guidelines.xhtml

Table 4. Multivariate model of performing health-promoting community activities (CA)

Variable	General model (n=497) OR (95% CI)
**Profession**	
Nurse	Ref.
Doctor	0.44 (0.24-0.82)
Paediatrician	1.55 (0.69-3.46)
Social worker	25.26 (2.53-251.9)
**Specific training in CA**	1.91 (1.17-3.11)
**Collaboration with NGO or civic entity**	1.91 (1.17-3.13)
**Possibility of work covered by colleagues**	1.80 (1.08-2.99)
**Support from colleagues in doing CA**	2.92 (1.49-5.73)
**Motivated to perform CA**	
Disagree or strongly disagree	Ref.
Agree	2.29 (1.28-4.08)
Strongly disagree	3.74 (1.85-7.54)
**Participates in activities in the neighbourhood**	3.06 (1.86-5.05)
**Years at health centre**†	1.06 (1.03-1.09)
**Nursing-student tutor**	1.96 (1.09-3.53)
**Model Validity**	**Value**
**Chi^2**	221.338
**Nagelkerke R squared**	.483

Discussion

Our study of the participation of health care workers in health-promoting activities in the community suggests that factors of the individual health care professional were more

For peer review only - http://bmjopen.bmj.com/site/about/guidelines.xhtml

important than those of the health care team or the community. In particular, the specific profession, attitudes, and opinions of an individual seemed to be crucial. Professional workload, which was one of the more mentioned factors by the professionals in others studies, does not have any impact when adjusted by other individual factors. This results confirms the frAC Project[29] hypothesis and those of other authors[21,28]

Our assessment of variables related with health care team participation in health promoting community activities suggests that team characteristics had greater impact than community characteristics. One exception was the socio-economic level of the community. This makes sense in the context of the Spanish health care system, which attempts to offer equitable care to people according to their needs and community interventions are important tools to reduce inequalities in health care[35]. It should be noted that we found no relationship between the level of social interaction in the community and the implementation of community activities, in contrast with the findings of other authors[16-17].

Our results showing the important role of nurses in health promoting community activities are consistent with those of other authors[13-14,17,36] who highlighted the role of nurses in the development of community activities. This result is expected because nurses usually receive more training in community care. Another crucial group is social workers. Previous research reported the importance of social workers[13], but their role in the community varies in different regions of Spain. Their presence seems fundamental for development of health-promoting community activities in regions where they have significant roles in the community. In Spain, community work is generally not provided as part of primary health care; instead, it is relegated to professional volunteers or specific programs. Thus, our results recognize the importance of work by nurses and social workers and points to the possibility that their roles could be modified or clarified. We also found that health centres that train undergraduate nurses have a lower level of community activities but that professional nurse-

For peer review only - http://bmjopen.bmj.com/site/about/guidelines.xhtml

trainers have a greater involvement in health promoting community activities. This apparent contradiction may be resolved by considering two factors. First, the variable at the health centre level included training in various disciplines (from nursing to physiotherapy) without distinction, even though there is clearly difference among these in communitarian tradition. Second, the need for increased training would make it difficult for the health team to engage in community activities although, among the cases trainers carried out more activities than others. Health care workers who worked for more years at a centre were also more likely to perform community activities. This may be because medium- and long-term projects require continuity and stability, and it is more difficult for professionals who continually change centres or have temporary contracts to participate in health promoting community activities.

The pressures of working in health care and the heavy work burden were the main arguments offered by professionals when asked about their low participation in health promotion and prevention activities[19,22,24-27]. Our bivariate analysis supported this relationship, although the multivariate analysis did not. This implies that the professionals' perception was incorrect. In fact, the pressure of working in health care and the heavy work burden are not associated with low participation in health promoting community activities.

Our results indicated the importance of the opinions and attitudes of professionals when performing health promoting community activities, in accordance with other studies[15,21-22]. In fact, all variables regarding the opinions and attitudes of health care workers were significantly different between cases and controls. This suggests that individuals who perform community activities have similar attitudes: they employ a biopsychosocial[12,37] practice model, are more oriented towards health promotion, show recognition of the need for citizen participation in health services, and are more left-leaning in ideology. We also found that social sensitivity was an important variable in the model for professionals. This variable measures involvement of the professional with the community beyond consultation work. It is

For peer review only - http://bmjopen.bmj.com/site/about/guidelines.xhtml

not clear how some practices, responsibility for which is stipulated in primary care legislation and which should be similar for all professionals to assure fairness within the system, can be affected by a professional's personal discourse. Most health care professionals that we interviewed – both cases and controls -- said that their community roles were not well defined. This suggests that greater priority should be given to develop health promoting community activities in primary health care, and to clarify the role and responsibility of every professional about this question.

We found that having support within the health care team is associated with performing health promoting community activities. This finding should be highlighted, because all the professionals in this study were selected from the centres that engage in community activities so that, presumably, all cases can count on some support from their centres. It may be that some professionals were unaware of what their colleagues were doing, or that there were barriers to working with particular colleagues. Regardless of the explanation, this result suggests that application of the teamwork model[38], an assumed key feature of primary health care, has certain weaknesses.

Strengths and limitations

This study had an observational design, so we could not definitively establish causal relationships. Similarly, our definition of "health promoting community activities" may be a limitation because this concept can be defined or perceived in different ways; we made an attempt to control for this by use of a definition generated by expert consensus, which we then transformed into an algorithm. The questionnaires we used were *ad hoc* and some of the data regarding team variables, such as degree of social interaction in the community and the relationship between professionals, were provided by team managers. This may have led to a bias in information gathering due to a lack of knowledge or other unknown factors. Some variables that we did not examine could have explained participation in community activities,

For peer review only - http://bmjopen.bmj.com/site/about/guidelines.xhtml

such as support from primary care management, the health care administration and the centres' own coordinators or nursing managers. It would be valuable to assess the effect of these variables in future studies of the effect of team activities and priorities.

The main strengths of this study are that it examined a facet of health care that is relatively unexplored; the study subjects were from 5 different regions of Spain; the sample size was large; and it considered many distinct factors simultaneously. Furthermore, our design of the second study, in which cases and controls were matched by teams, adjusted for the effect of team and community variables, so a multilevel analysis was not necessary.

Implications for health care practice

Health care administrators who want to develop health-promotion activities for the community must provide visible and formal support for community initiatives, define better the expected communitarian tasks of each sanitarian professional, and provide job stability for all participating workers. Specific training is also important for the formation of health-promoting community activities. It may seem that attitudes, social sensitivity, or personal opinions are not modifiable; however, in our opinion, it is possible to modify these factors during undergraduate education. So, community perspective and their associated responsibility should be included in undergraduate sanitary formation.

Conclusions

A key responsibility of primary health care is provision of health-promoting community activities. We found that the actual implementation of these activities depends more on the characteristics of individual professionals than on the characteristics and composition of the health care team or the community. There were also significant differences among different types of professionals, and the contributions of nurses and social workers are fundamental. The pressures perceived by members of the health care team were not associated with involvement in community activities.

For peer review only - http://bmjopen.bmj.com/site/about/guidelines.xhtml

Acknowledgements

We want to express thanks to Magdalena Esteva for her patient reading of the manuscript.

Competing interests

All authors have completed the ICMJE uniform disclosure form at www.icmje.org/coi_disclosure.pdf and declare: no financial relationships with any organisations that might have an interest in the submitted work in the previous three years; no other relationships or activities that could appear to have influenced the submitted work

Funding

This study was supported by a grant from the Carlos III Health Institute (PI07/90383; PI07/90925; PI07/90636). It also received support from the Health Promotion and Preventive Activities in Primary Health Care Research Network (IAPP network RD 06/0018/), the ERDF funds, and the Institut de Salut de Palma (IDISPA). SM was contracted with the aid of a grant to stabilise employment in health research from the Carlos III Health Institute and benefited from a grant for further studies from the same institution. All institutions are public and did not have any role in study design, analysis, or publication.

Contributors

SM was the lead investigator, and was responsible for conception and design of the study, analysing data and writing the manuscript. **MR** has the original idea of the study. **SM, JR, JLRG, IMG, CBBA,** and **LES**, coordinated the field work in everyone of the regions involved. SM, JR, MJM, EZO, CBBA, LES, CV, MLLBA, TPJ, DMPM, MLLS, JLRG, RBV, CSV, MRB, MCV, MBC, ESF, MBD, IMG, BBR, AAL, SBC, ACT, BOB, RMB, PMP, MEO, MJPJ, MR contributed to the design and development of the study, have read the manuscript critically, made contributions and approved the final version.

Data Sharing Statement: Questionnaires and anonymised data are available on demand.

For peer review only - http://bmjopen.bmj.com/site/about/guidelines.xhtml

References

1. World Health Organisation. **The Ottawa Charter for Health Promotion**, Health Promotion. 1986 1 (4): i-v.

2. World Health Organization. **Health Promotion Glossary.** WHO 1998. [http://apps.who.int/iris/bitstream/10665/64546/1/WHO_HPR_HEP_98.1.pdf]

3. World Health Organisation: **Declaration of Alma Ata.** *WHO* 1978. [http://www.who.int/hpr/NPH/docs/declaration_almaata.pdf]

4. World Health Organisation: **Declarations of Jakarta** WHO 1997. [http://www.who.int/hpr/NPH/docs/jakarta_declaration_sp.pdf]

5. March S, Torres E, Ramos M. **Adult community health-promoting interventions in primary health care: A systematic review.** *Prev Med* 2015; 76 (2015) S94–S104

6. Ley General de Sanidad 14/1986, de 25 de abril. Boletín Oficial del Estado,nº102,(29/4/1986).

7. Real Decreto 1030/2006 del 15 de septiembre por el que se establece la cartera de servicios comunes del Sistema Nacional de Salud y el procedimiento para su actualización. *Boletín Oficial del Estado*,nº222,(16-9-2006).

8. Guldan GS: **Obstacles to community Health promotion**. *Soc sci med* 1996, **5**:689-695.

9. Irigoyen J: **La participación comunitaria en salud: Una reformulación necesaria.** In: *Nuevas perspectivas en Atención Primaria de Salud.* Edited by Aranda. Madrid: Díaz de Santos; 1994:183-221.

10. Gérvas J, Pérez-Fernández M, Palomo-Cobos L, Pastor-Sánchez R: **Veinte años de reforma de la Atención Primaria en España. Valoración para un aprendizaje por acierto/error.** Madrid: Ministerio Sanidad y Consumo 2005. [http://equipocesca.org/new/wp-content/uploads/2010/08/20-anos-de-reforma-de-la-ap-en-espana.pdf]

For peer review only - http://bmjopen.bmj.com/site/about/guidelines.xhtml

11. Regato P, Domínguez J, Sancho M: **Coordinación sanitaria y abordaje comunitario en atención primaria**. In: *La Atención Primaria de Salud en España y en sus comunidades autónomas.* Coordinated by Navarro V, Martín-Zurro A. Barcelona: Semfyc; 2009:149-168.

12. Turabián JL, Pérez B: **Grandes misterios. ¿Puedes ver al monstruo del lago Ness?: El modelo biopsicosocial y las actividades comunitarias.** *Aten Primaria* 2007, **39**:261-4.

13. March S, Soler M, Miller F, et al: **Variabilidad en la implantación de las actividades comunitarias de promoción de la salud en España.** *An Sist Sanit Navar.* In press.

14. Sáez S, Marqués F, Pallarés J, Serrano J, Orrio C, Martínez V: **Perfil de los profesionales de la salud de Lleida respecto a la educación para la salud (EpS).** *Aten Primaria* 1999, **24**:157-161.

15. Pérez-Franco B, Turabián JL, Magaña E, et al: **Factores asociados a la participación comunitaria en la atención primaria de la provincia de Toledo.** *Aten primaria* 1993, **12**: 339-344.

16. MJ Aguilar Idáñez: **La participación comunitaria en salud: ¿Mito o realidad?** Madrid: Díaz de Santos; 2001.

17. López E, Forcada C, Miller Frederick: **Factores que facilitan y dificultan el desarrollo de los proyectos comunitarios. Estudio observacional de la red AUPA de centros de atención primaria de Cataluña.** *Aten primaria* 2010, **42**:218-25.

18. Duro JC: **Actitudes de los profesionales de atención primaria de Madrid sobre los grupos.** *Aten Primaria* 2005, **36**:71-7.

19. Díaz G: **Factores relacionados con la práctica de educación sanitaria por los médicos de Atención primaria de Galicia.** *Rev Esp Salud Pública* 2001, **75**:529-540.

For peer review only

For peer review only - http://bmjopen.bmj.com/site/about/guidelines.xhtml

20. Maheux B Dufort, F Lambert J et al: **The professional attitudes and clinical practices of men and women generalists.** *Can Fam Physician* 1989, **35**:59-63.

21. Maheux B, Pineault R, Lambert J et al: **Factors influencing physicians' preventive practices.** *Am J Prev Med* 1989, **5**:201-206.

22. Myrand A, Beehler G, Kuo C, Mahoney M: **Explaining the de-prioritization of primary prevention: Physicians' perceptions of their role in the delivery of primary care.** *BMC Public Health* 2003, **3**:15. doi:10.1186/1471-2458-3-15.

23. Walter U, Flick U, Neuber A, et al: **Putting prevention into practice: qualitative study of factors that inhibit and promote preventive care by general practitioners, with a focus on elderly patients.** *BMC Family practice* 2010, **11**:68. doi:10.1186/1471-2296-11-68

24. Puig ribera A, McKenna J, Riddoch C: **Attitudes and practices of physicians and nurses regarding physical activity promotion in the Catalan primary health-care system.** *Europ J Public Health* 2005, **15**:569–75.

25. Wilhemsson S, Lindberg L: **Health promotion: Facilitators and barriers perceived by district nurses.** *Int J Nurs Practice* 2009, **15**:156-163.

26. Rubio-Valera M, Pons-Vigues M, Martinez-Andres M et al. **Barriers and Facilitators for the implementation of primary prevention and health promotion activities in primary care: A Syntethesis through meta-etnography.** Plos one 2014;9:e89554. Doi: 10.1371/journal.pone.0089554.

27. Badertscher N, Rossi PO, Rieder A et al: **Attitudes, barriers and facilitators for health promotion in the elderly in primary care.** *Swiss Med Wkly* 2012;**142**:w13606.

28. Maheux B, Pineault L, Béland F et al: **Factors influencing physicians' orientation toward prevention.** *Am J Prev Med.* 1987, **3**:12-8.

For peer review only

For peer review only - http://bmjopen.bmj.com/site/about/guidelines.xhtml

29. March S, Ripoll J,Ruiz-Giménez JL, et al: **Observational study on factors related to health-promoting community activity development in primary care (frAC Project): a study protocol.** *BMJ Open* 2012, **2**. e001287 doi:10.1136/bmjopen-2012-001287.

30. March S, Jordán M, Montaner I et al. **¿Qué hacemos en el barrio? Descripción de las actividades comunitarias de promoción de la salud en atención primaria: Proyecto frAC.** Gac Sanit 2014; **28**:267-73.

31. March S, Ramos M, Soler M, Ruiz-JiménezJL, Miller F, Domínguez J, et al: **Revisión documental de experiencias de actividad comunitaria en atención primaria de salud.** *Aten Primaria* 2011, **43**:289-296.

32. T Martínez-cañavate: **Modelo multinivel explicativo de la utilización de las consultas de atención primaria en Andalucía**. *Phd tesis.* Granada university, faculty of medicine; 2007. [http://hera.ugr.es/tesisugr/16905234.pdf]

33. Mira JJ, Llinás G, Gil V, Orozco D, Palazón I, Vitaller J: **Validación de un instrumento para identificar estilos de práctica profesional del médico de atención primaria.** *Aten Primaria* 1998, **21**:14-22.

34. Rey del Castillo P: **Nota metodológica sobre los indicadores del barómetro.** *Rev Española de Investigaciones Sociológicas* 2004, **108**:151-78.

35. Comisión para reducir las desigualdades en salud en España: **Propuesta de políticas e intervenciones para reducir las desigualdades en salud en Espanya**. *Gac Sanit* 2012. doi:10.1016/j.gaceta.2011.07.024.

36. Hogg W, Dahrouge S. Russell G et al: **Health promotion activity in primary care: performance of models and associated factors.** *Open medicine* 2008, **3**:165-73.

37. Engel GL: **The need for a new medical model: a challenge to biomedicine.** Science 1977, **196**:129–36.

For peer review only - http://bmjopen.bmj.com/site/about/guidelines.xhtml

38. Grumbach K, Bodenheimer T: **Can Health Care teams improve Primary Care practice?** JAMA 2004, **291**:1246-51.

For peer review only

For peer review only - http://bmjopen.bmj.com/site/about/guidelines.xhtml

STROBE Statement—Checklist of items that should be included in reports of ***case-control studies***

	Item No	Recommendation
**Title and abstract**	1	(*a*) Indicate the study's design with a commonly used term in the title or the abstract<br>OK. p1.
		(*b*) Provide in the abstract an informative and balanced summary of what was done and what was found<br>OK. p2
**Introduction**		
Background/rationale	2	Explain the scientific background and rationale for the investigation being reported<br>OK. p4-5
Objectives	3	State specific objectives, including any prespecified hypotheses<br>OK. p5
**Methods**		
Study design	4	Present key elements of study design early in the paper<br>OK. p5
Setting	5	Describe the setting, locations, and relevant dates, including periods of recruitment, exposure, follow-up, and data collection<br>OK. p6
Participants	6	(*a*) Give the eligibility criteria, and the sources and methods of case ascertainment and control selection. Give the rationale for the choice of cases and controls<br>OK. p6
		(*b*) For matched studies, give matching criteria and the number of controls per case<br>OK. p6.
Variables	7	Clearly define all outcomes, exposures, predictors, potential confounders, and effect modifiers. Give diagnostic criteria, if applicable<br>OK. p6-8.
Data sources/ measurement	8*	For each variable of interest, give sources of data and details of methods of assessment (measurement). Describe comparability of assessment methods if there is more than one group<br>OK. p6-8
Bias	9	Describe any efforts to address potential sources of bias<br>OK. p17-18
Study size	10	Explain how the study size was arrived at<br>OK. p8
Quantitative variables	11	Explain how quantitative variables were handled in the analyses. If applicable, describe which groupings were chosen and why<br>OK. p6-8
Statistical methods	12	(*a*) Describe all statistical methods, including those used to control for confounding<br>OK. p8-9
		(*b*) Describe any methods used to examine subgroups and interactions<br>OK. p8-9
		(*c*) Explain how missing data were addressed<br>OK. p8-9
		(*d*) If applicable, explain how matching of cases and controls was addressed<br>Not applicable
		(*e*) Describe any sensitivity analyses

For peer review only

		Not applicable
**Results**		
Participants	13*	(a) Report numbers of individuals at each stage of study—eg numbers potentially eligible, examined for eligibility, confirmed eligible, included in the study, completing follow-up, and analysed<br>OK. p11
		(b) Give reasons for non-participation at each stage<br>OK. p11
		(c) Consider use of a flow diagram<br>OK. It is not necessary.
Descriptive data	14*	(a) Give characteristics of study participants (eg demographic, clinical, social) and information on exposures and potential confounders<br>OK. Tables 1 and 2.
		(b) Indicate number of participants with missing data for each variable of interest<br>OK. Tables 1-3.
Outcome data	15*	Report numbers in each exposure category, or summary measures of exposure<br>OK. Tables 1-3.
Main results	16	(*a*) Give unadjusted estimates and, if applicable, confounder-adjusted estimates and their precision (eg, 95% confidence interval). Make clear which confounders were adjusted for and why they were included<br>OK. Tables 1-3.
		(*b*) Report category boundaries when continuous variables were categorized<br>OK. Tables 1-3.
		(*c*) If relevant, consider translating estimates of relative risk into absolute risk for a meaningful time period<br>OK. It is not necessary.

For peer review only

Other analyses	17	Report other analyses done—eg analyses of subgroups and interactions, and sensitivity analyses<br>OK. p8-9.
**Discussion**		
Key results	18	Summarise key results with reference to study objectives<br>OK. p14-15.
Limitations	19	Discuss limitations of the study, taking into account sources of potential bias or imprecision. Discuss both direction and magnitude of any potential bias<br>OK. p17-18.
Interpretation	20	Give a cautious overall interpretation of results considering objectives, limitations, multiplicity of analyses, results from similar studies, and other relevant evidence<br>OK. p15-18.
Generalisability	21	Discuss the generalisability (external validity) of the study results<br>OK. p17-18
**Other information**		
Funding	22	Give the source of funding and the role of the funders for the present study and, if applicable, for the original study on which the present article is based<br>OK. p19

*Give information separately for cases and controls.

Note: An Explanation and Elaboration article discusses each checklist item and gives methodological background and published examples of transparent reporting. The STROBE checklist is best used in conjunction with this article (freely available on the Web sites of PLoS Medicine at http://www.plosmedicine.org/, Annals of Internal Medicine at http://www.annals.org/, and Epidemiology at http://www.epidem.com/). Information on the STROBE Initiative is available at http://www.strobe-statement.org.

For peer review only

BMJ Open

Factors related to the development of health-promoting community activities in Spanish primary health care: Two case-control studies

Journal:	*BMJ Open*
Manuscript ID	bmjopen-2017-015934.R2
Article Type:	Research
Date Submitted by the Author:	02-Jun-2017
Complete List of Authors:	March, Sebastià; Baleares Health services-IbSalut, Primary Care Research Unit of Mallorca; Instituto de investigación en salud de Palma (IDISPA)<br>Ripoll, Joana; Baleares Health services-IbSalut, Primary Care Research Unit of Mallorca; Baleares Health services-IbSalut, Primary Care Research Unit of Mallorca<br>Jordan Martin, Matilde Jordan Martin; Estrecho de Correa Primary health center, Madrid Health Services<br>Zabaleta del Olmo, Nieves; Fundacio Jordi Gol i Gurina, IDIAP<br>Benedé Azagra, Carmen Belén; Aragon Health Services, Oliver Primary health center<br>Elizalde Soto, Lázaro; Gobierno de Navarra Departamento de Salud, public health institute; Public Health and epidemiology CIBER,<br>Vidal, Mª Clara; Baleares Health services-IbSalut, Primary Care Research Unit of Mallorca; Instituto de investigación en salud de Palma (IDISPA)<br>Bauzà Amengual, M. de Lluc; Balearic Islands University,<br>Planas Juan, Trinidad; Baleares Health services-IbSalut, Son Gotleu Primary health care center<br>Maria Pèrez, Damiana Maria Pèrez; Son Gotleu Primary health center, Baleares Health services-IbSalut<br>Llull Sarralde, Micaela; Baleares Health services-IbSalut, San Agustí Primary health care center<br>Ruiz-Giménez, Juan Luís; Madrid Health Services, Madrid, Vicente Soldevilla Primary health care center<br>Bajo Viñas, Rosa; Madrid Health care services, Loeches Primary health care center<br>Solano Villarubia, Carmen Solano Villarubia; madrid Health care services, DA Suroeste Health center<br>Rodriguez Bajo, Maria<br>Cordoba Victoria, Manuela Cordoba Victoria; SOMAMFyC<br>Badia Capdevila, Marta; Institut Catala De La Salut, El carmel Health center<br>Serrano Ferrandez, Elena; Institut Catala De La Salut, El carmel health center<br>Bosom Diumenjo, Maria; Institut Catala De La Salut, Sant Rafel Primary Health Center<br>Montaner-Gomis, Isabel; Institut Catala De La Salut, El Carmel Health center<br>Bolibar-Ribas, Buenaventura; Fundacio Jordi Gol i Gurina, IDIAP<br>Antoñanzas Lombarte, Angel; Aragon Health services, Delicias del Sur health center

Bregel Cotaina, Samantha; Instituto Aragones de Ciencias de la Salud, Primary health care research unit
Calvo Tocado, Ana; Zaragoza Public Health provincial subdirection
Olivan Blázquez, Barbara; Instituto Aragones de Ciencias de la Salud, primary health care research unit
Magallon Botaya, Rosa; Aragon Health services, Arrabal health center
Marín Palacios, Pilar Marín Palacios; Gobierno de Navarra Departamento de Salud, Public health institute
Echauri Ozcoidi, Margarita; Gobierno de Navarra Departamento de Salud, public health institute
Perez-Jarauta, Mª Jose Perez-Jarauta; Gobierno de Navarra Departamento de Salud, public health institute
Llobera, Joan; Baleares Health services-IbSalut, 1. Primary Care Research Unit of Mallorca; Instituto de investigación en salud de Palma (IDISPA)
Ramos, Maria; Govern de les Illes Balears Conselleria de Salut Familia i Benestar Social, Direcció General de salut Pública i Participació; Instituto de investigación en salud de Palma (IDISPA)

**Primary Subject Heading**:	General practice / Family practice
Secondary Subject Heading:	Health services research, Public health
Keywords:	Health promotion, Primary health care, Health services research, Community health, Health team, Health professionals

SCHOLARONE™
Manuscripts

For peer review only

Factors related to the development of health-promoting community activities in Spanish primary health care: Two case-control studies

Sebastià March[1], Joana Ripoll[1], Matilde Jordan Martin[2], Edurne Zabaleta del Olmo[3], Carmen Belén Benedé Azagra[4], Lázaro Elizalde Soto[5], Mª Clara Vidal[1], María de Lluc Bauzà Amengual[6], Trinidad Planas Juan[7], Damiana Maria Pérez Mariano[7], Micaela Llull Sarralde[8], Juan Luís Ruiz-Giménez[9], Rosa Bajo Viñas[10] , Carmen Solano Villarubia[11], Maria Rodriguez Bajo[12], Manuela Cordoba Victoria[13], Marta Badia Capdevila[3], Elena Serrano Ferrandez[3], Maria Bosom Diumenjo[14], Isabel Montaner Gomis[15], Bonaventura Bolívar Ribas[3], Angel Antoñanzas Lombarte[16], Samantha Bregel Cotaina[17], Ana Calvo Tocado[18], Barbara Olivan Blázquez[17], Rosa Magallón Botaya[19], Pilar Marín Palacios[5], Margarita Echauri Ozcoidi[5], María Jose Perez-Jarauta[5], Joan Llobera[1], Maria Ramos[20].

1. Primary Care Research Unit of Mallorca, Baleares Health Services-IbSalut, Palma de Mallorca, Spain.
2. Estrecho de Correa Primary Health Center, Madrid Health Services, Madrid, Spain.
3. Primary Care Health Research Institute IDIAP Jordi Gol, Barcelona, Spain.
4. Oliver Primary Health Center, Aragon Health Services, Zaragoza, Spain.
5. Navarra Public Health Institute, and Public Health and Epidemiology CIBER, Pamplona, Spain.
6. Balearic Islands University, Palma de Mallorca, Spain.
7. Son Gotleu Primary Health Center, Baleares Health Services-IbSalut, Palma de Mallorca, Spain.
8. San Agustí Primary Health Center, Baleares Health Services-IbSalut, Palma de Mallorca, Spain.
9. Vicente Soldevilla Primary Health Center, Madrid Health Services, Madrid, Spain.
10. Loeches Primary Health Center, Madrid Health Services, Madrid, Spain.
11. DA Sureste Primary Health Center, Madrid Health Services, Madrid, Spain.
12. Freelance, Madrid, Spain
13. SOMAMFYC, Madrid, Spain
14. Sant Rafel Primary Health Center, Catalonian Health Services Cat-Salut, Barcelona, Spain.
15. El Carmel Primary Health Center, Catalonian Health Services Cat-Salut, Barcelona, Spain.
16. Delicias del Sur Primary Health Center, Aragon Health Services, Zaragoza, Spain.
17. Primary Care Research Unit, Aragon, Spain
18. Zaragoza Public Health department, Zaragoza, Spain.
19. Arrabal Primary Health Center, Aragon Health Services, Zaragoza, Spain.
20. Public Health Department, Balearic Islands Health Department, Spain.

Corresponding author:
Sebastià March
E-mail: smarch@ibsalut.caib.es,

For peer review only

Abstract

Objective: Spanish primary health care teams have the responsibility of performing health-promoting community activities, although such activities are not widespread. Our aim was to identify the factors related to participation in those activities.

Design: Two case-control studies. **Setting:** Performed in primary care of 5 Spanish regions. **Subjects:** In the first study, cases were teams that performed health promoting community activities and controls were those that did not. In the second study (on case teams from the first study), cases were professionals who developed these activities and controls were those who did not. **Main outcome measures:** Team, professional, and community characteristics collected through questionnaires (team managers/professionals) and from secondary sources.

Results: The first study examined 203 teams (103 cases, 100 controls). Adjusted factors associated with performing community activities were percentage of nurses (OR=1.07, 95%CI =1.01-1.14), community socio-economic status (higher *vs.* lower OR =2.16, 95%CI=1.18-3.95), and performing undergraduate training (OR=0.44, 95%CI=0.21-0.93). In the second study, 597 professionals responded (254 cases, 343 controls). Adjusted factors were professional classification (physicians do fewer activities than nurses and social workers do more), training in community activities (OR=1.9, 95%CI =1.2-3.1), team support (OR=2.9, 95%CI =1.5-5.7), seniority (OR=1.06, 95%CI=1.03-1.09), nursing tutor (OR =2.0, 95%CI =1.1-3.5), motivation (OR =3.7, 95%CI=1.8-7.5), collaboration with non-governmental organizations (OR=1.9, 95%CI=1.2-3.1), and participation in neighbourhood activities (OR=3.1, 95%CI=1.9-5.1).

Conclusions: Professional personal characteristics, as social sensitivity, profession, to feel team support, or motivation, have influence in performing health-promoting community

For peer review only - http://bmjopen.bmj.com/site/about/guidelines.xhtml

activities. In contrast to the opinion expressed by many professionals, workload is not related to performance of health-promoting community activities.

Keywords: Health promotion, Primary health care, Health services research, Community health, Health team, health professionals

Strengths and limitations of this study

- This study examined a topic relatively unexplored, as community health promotion by primary health care.

- A case-control approach was chosen, where cases were subjects (professionals or teams) who were developing community activities, and controls those who not.

- Design allows to explore many distinct factors simultaneously, from different sources, and in two levels: Health team and professional.

- An operative definition of community activities was used, which recognizes the heterogeneity of health promotion interventions developed with the community in Spanish primary health care.

- Because of its observational design, there are limitations to establishing causal inferences.

For peer review only

Introduction

The Ottawa Charter for Health Promotion[1] defined Health promotion as the process of enabling people to increase control over, and to improve, their health. This definition implies the importance of empowering people[2] to take control over what determines their health. One tool to work in this direction are health promoting community activities. These can come in a variety of forms, from walking groups in a neighbourhood to holding meetings with local authorities, schools or civil associations to develop specific health programs. Experts at numerous international conferences have agreed on the importance of health-promoting community activities for improving public health[1,3-4], and there is some evidence that these activities are effective[5].

Since the implementation of health care reform in Spain during the 1980's[6-7], primary health care teams have had responsibility for carrying out health promotion activities. These teams, located in health care centres throughout the country, are composed of physicians, community nurses, paediatricians, midwives, social workers, and other health care professionals (gynaecologists, psychologists, and psychiatrists) in a system that offers almost-universal and free health care. These conditions are ideal for an interdisciplinary approach to encourage health promoting activities. However, as in other countries[8], implementation of these interventions differs greatly among the different health care teams[9-11], and relatively few professionals are involved[11-13].

There has been limited studies of the factors related to the development of health-promoting interventions in the community. These factors may be broadly classified as related to the community, to the team, or to the individual professionals. With regard to community factors, some studies indicated that the rural/urban environment[14], size of the municipality[15], level of social interaction within the community[15-17], and presence of a focal point for participation in community health initiatives[15-16] influence the practice of health

For peer review only - http://bmjopen.bmj.com/site/about/guidelines.xhtml

promoting community activities. The primary health care team factors include job satisfaction[18], management of time and setting within the center[14], composition and organization of the team[15-16], years of team operation[15], intra-team support[17], and work burden[8,19]. Studies of professionals have highlighted sex[19-21], age[15,20], professional status[14], specific training[22-25], motivation[17-18], and the model applied (biomedical or psychosocial)[18]. The attitudes and beliefs of the professionals, such as trust in the effectiveness of preventive actions[18,22,25-27], self-reported efficacy in carrying out activities[8,22-23], or to support community participation[15,21] are also important. An additional element is professionals' social sensitivity, as evaluated through their personal political leanings[28]. According to professionals themselves, the most important factor is the lack of time available to assume the care burden[19,22,24-27]. All these features have been observed separately, mostly in descriptive studies.

This study presents some of the results of the Factors Related to Health-Promoting Community Activity Development in Primary Care (frAC) Project, whose complete methodology and aims were described elsewhere[29]. The purpose of this study is to identify primary health care team and community factors related to implementation of health promoting community activities in primary care and factors that may explain why only some professionals within a team participate in those community activities.

Methods

Two case-control studies were performed. In the first, the cases were the teams that performed health promoting community activities and the controls were those that did not. The second study examined only teams that implemented these community activities to adjust for community/team factors; in this second analysis the cases were professionals who developed these activities and the controls were those who did not. The study was set in

For peer review only - http://bmjopen.bmj.com/site/about/guidelines.xhtml

primary health care centers in five regions of Spain (Balearic Islands, Catalonia, Aragon, Madrid, and Navarra). Data collection was carried out between 2009-2010.

Selection of cases and controls

We used a conceptual definition of health promoting community activity reached in a previous study [30-31]. This was converted into an algorithm for use in this study[29]: this activity had to be a non-isolated activity carried out in the previous year, in which the professional participated on behalf of the health centre. Furthermore, it had to have involvement and active participation of the community, or the population had to have the capacity to influence the intervention, or it was a cross-sector activity involving collaboration with entities outside of health care (*e.g.* education, social services). Health preventive interventions which does not have a health promotion approach were excluded.

We telephoned all the health centres in the research areas asking if they had participated in health promoting community activities during the last year. If they did, we contacted a participant of the activity and gave him/her a questionnaire to confirm that the activity met our inclusion criteria. For the first study, health teams that participated in at least one community activity in the last year were selected as cases, and controls were those who had not participated. For the second study, we selected all professionals who participated in the implementation of those community activities as cases and professionals who did not participate as controls. In every health team we selected at least one control for every case.

Variables

Information was gathered through three distinct questionnaires (available on request) that were given to the community, the team, and the professionals[29]. Description of health promoting community activities are described elsewhere[30].

The data collected from the community (from secondary sources and from questionnaires to centre managers) were: demographic composition; socio-economic level; degree of social

For peer review only - http://bmjopen.bmj.com/site/about/guidelines.xhtml

interaction; health centre setting; geographical dispersion based on an ordinal variable used by the National Health System for resource allocation (from G1 [less dispersed] to G4 [more dispersed]); and existence of health boards (community participation agencies described in health legislation)[16].

The data collected from the team (collected from secondary sources and questionnaires to centre managers) were: percentages of different professionals (nurses, physicians, and paediatricians); year of opening; population of service area; professional/population and doctor/nurse ratios; availability of a space in the centre to carry out group activities and presence of a team member responsible for health education; collaboration with the area health board where applicable; training of resident physicians; nurses and nursing or physiotherapy students; evaluation of professional relationships between distinct professional disciplines (based on a Likert-type scale ranging from 1 [poor] to 5 [excellent]).

The data collected from the professionals (from individual self-administrated questionnaires) were: sex; age; profession; working situation; health care burden; size of quota allocated; average number and duration of consultations per day at the centre or at home; tutoring; participation in research or specific training in health promotion in the previous five years; years since graduation; years worked in primary care and as a member of the current health care team; autonomy to organize scheduling; arrangements with colleagues to cover health care tasks while carrying out community activities; support of fellow team members in conducting these activities; working atmosphere; job satisfaction; and self-assessed efficacy in conducting community work (based on a Likert-type scale ranging from 1 [poor] to 5 [excellent]).

Opinions and attitudes were measured by responses to various statements developed by the research team, using a Likert-type scale with four options ranging from "strongly

For peer review only - http://bmjopen.bmj.com/site/about/guidelines.xhtml

agree" to "strongly disagree". The statements dealt with trust in the effectiveness of the health promotion community activities, motivation to carry out these activities, citizen participation in health care decision-making, need to strengthen agencies involved in citizen participation in health care, responsibility for primary care in the community, definition of professional role, link between health education and community participation, and the effectiveness of the boards in driving community activities. A four-item validated scale was used to evaluate each professional's clinical practice style (biomedical-psychosocial)[32-33] by assessing the attitude towards the demands of psychosocial and clinical care at work. Finally, social sensitivity data were collected through proxy variables: participation during the previous twelve months in activities in the neighbourhood where the professional works; involvement in any NGO or civil association; and political tendency (assessed through a visual scale adapted from the Centre for Sociological Research[34] Spanish monthly barometer).

Calculation of sample size

For the first study, we calculated that at least 91 teams would be needed in each group based on an OR of 2.5, an alpha risk of 0.05, a beta of 0.2, and an expected proportion of 0.5 in the controls. For the second study, we calculated that at least 222 professionals would be needed in each group based on an OR of 1.75, an alpha risk of 0.05, a beta of 0.2, and an expected proportion of 0.5 in the controls.

Data analysis and management

A descriptive analysis of all variables was carried out to compare their distributions in each group. Categorical variables are expressed as percentages and continuous variables as means with 95% confidence intervals (95% CIs) or as medians and percentiles depending on the distribution. Main variable was, for both studies, to be case or control. The relationship with the main variable was assessed using the chi-squared test for categorical variables and Student's *t*-test and the Mann-Whitney U non-parametric test for continuous

For peer review only - http://bmjopen.bmj.com/site/about/guidelines.xhtml

variables. The strength of an association was expressed through unadjusted OR and its 95% CI.

We also performed a logistic regression analysis to calculate adjusted ORs. We constructed a first model using each variable whose *p*-value was less than 0.2 in the bivariate analysis. From this model, we tested and compared variations using the maximum likelihood method, eliminating variables that had *p*-values less than 0.05 in the Wald test. The collinearity of the variables was examined and interactions tested. We established a different model for the health care teams and for the professionals. All analyses were conducted using SPSS ver. 14 and Epidat ver. 3.1 software.

Ethical aspects

The study was approved by the Research Commissions in all the participant regions and by the Research Ethics Committee in Madrid (Area 1) and the University Research Institute in Primary Care (IDIAP) Jordi Gol in Barcelona. The questionnaire was completed anonymously by the professionals, with the identity of individuals and centres coded to ensure blinding of the researchers.

Results

Factors related to the health care team

We examined 203 health care teams (103 cases [50.7%] and 100 controls) in the first study. Table 1 shows the results of the initial bivariate analysis. The variables significantly related to team involvement in community activities were the socioeconomic level of the community, collaboration on training in physiotherapy or nursing, participation in a health board, percentage of nurses in the team, patient/physician ratio, and patient/nurse ratio.

For peer review only - http://bmjopen.bmj.com/site/about/guidelines.xhtml

Table 1. Relationship of community and primary health care team (PHT) characteristics with performance of health-promoting activities in the community.

Nominal Variables	PHT cases N (%)	PHT controls N (%)	Unadjusted OR (95% CI)
**Health Care setting**			
Urban	73 (71.6)	68 (68.0)	Ref.
Rural	29 (28.4)	32 (32.0)	0.84 (0.46-1.54)
**Socioeconomic level**			
High and medium-high	52 (51)	65 (65)	1
Medium-low and low	50 (49)	35 (35)	**1.79 (1.01-3.14)**
**Degree of social interaction**			
High	22 (23.9)	23 (26.1)	Ref.
Medium	43 (46.7)	35 (39.8)	1.28 (0.61-2.68)
Low	27 (29.3)	30 (34.1)	0.94 (0.43-2.06)
**Population of municipality**			
>1 million	47 (45.6)	48(48)	Ref.
500,000-1 million	7 (12.6)	7 (7)	1.90 (0.70-5.18)
10,000-500,000	22 (21.4)	33 (33)	0.68 (0.35-1.33)
<10,000	21 (20.4)	12 (12)	1.79 (0.79-4.04)
**Degree of geographical dispersion**			
G1 (less dispersed)	62 (66.7)	60 (63.8)	Ref.
G2	11 (11.8)	17 (18.1)	0.63 (0.27-1.45)
G3	9 (9.7)	11 (11.7)	0.79 (0.31-2.05)
G4 (more dispersed)	11 (11.8)	6(6.4)	1.77 (0.67-5.10)
**Active health board**	67 (66.3)	60 (61.9)	1.21 (0.68-2.17)
**Training centre**			
Resident Doctors	35 (34.7)	34 (34.3)	1.01 (0.56-1.82)
Resident Nurses	23 (23.5)	29 (30.2)	0.71 (0.37-1.34)
Nursing/physiotherapy students	73 (72.3)	84 (85.7)	**0.43 (0.21-0.89)**
**Space for group activities**	83 (81.4)	79 (79)	1.16 (0.58-2.32)
**Health education manager**	45 (44.1)	46 (46)	0.93 (0.53-1.61)
**Participation in health board**	56 (83.6)	38 (63.3)	**2.95 (1.28-6.78)**
**Continuous variables**	**Mean (95% CI)**		**p***
PHT operational years	14.3 (13-15.2)	12.9 (11.7-14.1)	.075
Total No. professionals	36.4 (33.1-39.6)	37.5 (34-41.1)	.486
% nurses	35.4 (34.5-36.4)	33.5 (32.4-34.5)	**.010**
% paediatricians	7.0 (6.3-7.7)	7.4 (6.7-8.1)	.450
Population covered by PHT	20031 (18059-22002)	21770.4 (19530-24010)	.247
	**Median (IQR)**		**p****
% doctors	33.3 (29.5-38.3)	33.3 (29.9-36.4)	.532
% non-EU immigrants	11.1 (5.8-16.2)	11.1 (8.6-15.7)	.690
% population <15 years	13.0 (11.6-14.7)	12.9 (11.3-14.4)	.352
% population >65 years	17.8 (15.0-21.8)	18.9 (13.8-21.5)	.976
% population women	51 (49.5-53)	51.2 (50.3-53.1)	.403
Population ratio registered/professional	524 (444-658)	578 (482-669)	.122
Population ratio registered/doctor	1659 (1454-1905)	1734 (1533-2040)	**.034**
Population ratio registered/nurse	1508 (1284-1847)	1723 (1434-2057)	**.008**
Doctor/nurse ratio	.94 (.86-1.09)	1 (.89-1.14)	.085
**Professional relationships**			
Doctor::nurse	4 (3-4)	4 (3-4)	.376
Doctor::social worker	4 (3-5)	4 (3-4)	.978
Nurse::social worker	4 (3-5)	4 (3-4)	.845

Data from Spain 2009. For dichotomous variables, the reference value is always absence of the variable.* Student's t-test P. ** Mann-Whitney U test P. Bold-face: $p \leq 0.05$.

For peer review only - http://bmjopen.bmj.com/site/about/guidelines.xhtml

Eleven teams (5.0%) were excluded from the logistic regression analysis due to missing data. The 3 variables remaining in the final model were percentage of nurses in the team (OR = 1.07 for every 1% increase; 95% CI = 1.01-1.14); socio-economic level of the community (high and medium-high *vs.* low and medium-low OR = 2.16; 95% CI = 1.18-3.95); and having undergraduate training at the health care centre (OR = 0.44; 95% CI = 0.21-0.93). Nagelkerke's R squared was 0.11.

Factors related to professionals

A total of 597 professionals responded to the questionnaire, 254 (42.4%) cases and 343 (57.5%) controls. Refused to participate 96 professionals (62 cases and 34 controls). Participants were 298 nurses (52.4%), 191 doctors (33.6%), 58 paediatricians (10.2%), and 22 social workers (3.8%). Tables 2 and 3 show the results of the bivariate descriptive analysis. Table 2 shows that the variables related to involvement in health promoting community activities were sex, profession, training, self-confidence, and work situation. Also years of experience, age, workload, practice style and political orientation seems to be related. Table 3 summarizes the responses of the professionals' opinions and attitudes to health promotion, community participation, and social sensitivity.

For peer review only

For peer review only - http://bmjopen.bmj.com/site/about/guidelines.xhtml

Table 2. Relationship of professional variables with performance of health-promoting community activities (CA).

Nominal Variables	Cases N (%)	Controls N (%)	Unadjusted OR (95% CI)*
**Sex**			
Male	41 (16.4)	84 (25.3)	Ref.
Female	209 (83.6)	248 (74.7)	**1.72 (1.13-2.61)**
**Profession**			
Nurse	156 (64.7)	142 (43.3)	Ref.
Doctor	40 (16.6)	151 (46)	**0.24 (0.16-0.36)**
Paediatrician	24 (10)	34 (10.4)	0.64 (0.36-1.3)
Social worker	21 (8.7)	1 (0.3)	**19.11 (2.53-143.9)**
**Specialized General practitioner**	22 (55)	91 (60.3)	0.81 (0.39-1.62)
**Tutor of Resident doctors**	10 (25)	37 (24.5)	1.02 (0.45-2.3)
**Nursing tutors**	93 (59.6)	58 (40.8)	**2.13 (1.34-3.39)**
**Work situation**			
Permanent	204 (80.3)	238 (70)	Ref.
Temporary contracts	40 (15.7)	74 (21.8)	**0.63 (0.41-0.96)**
Other	10 (3.9)	28 (8.2)	**0.41 (0.19-0.87)**
**Participation in research projects in last 5 years**	115 (47.7)	147 (43.9)	1.16 (0.83-1.62)
**Specific training in CA**	142 (58.2)	104 (31.7)	**2.99 (2.12-4.23)**
**Job satisfaction**			
None/little	18 (7.1)	34 (10)	Ref.
Average	64 (25.3)	129 (38.1)	0.93 (0.49-1.78)
Quite a lot/a lot	171 (67.5)	176 (51.9)	1.83 (0.99-3.37)
**Working environment satisfaction**			
None/little	14 (5.6)	33 (9.8)	Ref.
Average	82 (32.5)	103 (30.5)	1.87 (0.94-3.73)
Quite a lot/a lot	156 (61.9)	202 (59.8)	1.82 (0.94-3.51)
**Self-confidence to carry out CA**			
None/little	31 (12.3)	72 (21.6)	Ref.
Average	81 (32.1)	154 (46.2)	1.22 (0.74-2.01)
Quite a lot/a lot	140 (55.5)	107 (32.1)	**3.03 (1.86-4.96)**
**Continuous Variables**	**Median (p25-p75)**		**p***
**Age**	49 (42-54)	48 (39-53)	**.048**
**Years qualified**	25 (18-31)	24 (15-29)	**.024**
**Years in primary care**	19 (11-23)	17 (10-23)	.552
**Years in current health centre**	11 (4-18)	7 (2-15)	**.000**
**Size of assigned quota**	1737 (1300-2043)	1600 (1300-1900)	**.026**
**Average daily consultations**	20 (15-30)	30 (20-35)	**.000**
**Average daily home visits**	1 (0.5-3)	1 (1-2)	.932
**Average consultation time**	10 (10-15)	10 (7-12)	**.000**
**Average home-visit time**	25 (20-30)	20 (15-30)	**.026**
**Biomedical practice style (+) Psychosocial (-)**	8 (6-9)	8 (7-9)	**.040**
**Political orientation: Left (-) right (+).**	2 (1-5)	3 (2-5)	**.026**

Data from Spain 2009. For dichotomous variables, the reference value is always absence of the variable. Bold-face: Chi-squared p ≤less than 0.05. * Mann-Whitney U test.

For peer review only - http://bmjopen.bmj.com/site/about/guidelines.xhtml

Table 3. Professional attitudes in cases and controls regarding health-promoting community activities (CA).

Variable	Cases N (%)	Controls N (%)	Unadjusted OR (IC 95%)
**Confidence in CA efficacy**			
Disagree/strongly disagree	9 (3.6)	19 (5.7)	Ref.
Agree	110 (43.8)	196 (59.0)	1.18 (0.51-2.71)
Strongly agree	132 (52.6)	117 (35.2)	**2.38 (1.03-5.46)**
**Motivated to carry out CA**			
Disagree- strongly disagree	44 (17.5)	149 (45.4)	Ref.
Agree	113 (45.0)	131 (39.9)	**2.92 (1.91-4.44)**
Strongly agree	94 (37.5)	48 (14.6)	**6.63 (4.08-10.7)**
**Citizen participation in health**			
Disagree/strongly disagree	23 (9.2)	46 (14.0)	Ref.
Agree	122 (49.0)	191 (58.1)	1.27 (0.73-2.21)
Strongly agree	104 (41.8)	92 (28.0)	**2.26 (1.27-4.01)**
**Citizen participation in health services**			
Disagree/strongly disagree	24 (9.6)	48 (14.5)	Ref.
Agree	109 (43.6)	198 (59.8)	1.10 (0.64-1.89)
Strongly agree	117 (46.8)	85 (25.7)	**2.75 (1.56-4.83)**
**Professionals' role in the community is well-defined**			
Disagree/strongly disagree	126 (51)	223 (66.8)	Ref.
Agree	103 (41.7)	97 (29)	**1.87 (1.32-2.67)**
Strongly agree	18 (7.3)	14 (4.2)	**2.27 (1.09-4.73)**
**Health education and community participation are closely linked**			
Disagree/strongly disagree	20 (8.1)	39 (11.7)	Ref.
Agree	128 (51.6)	194 (58.4)	1.28 (0.71-2.31)
Strongly disagree	100 (40.3)	99 (29.8)	**1.97 (1.07-3.61)**
**The health board drives CA**			
Disagree/strongly disagree	80 (33.8)	125 (39.8)	Ref.
Agree	123 (51.9)	163 (51.9)	1.17 (0.81-1.69)
Strongly agree	34 (14.3)	26 (8.3)	**2.04 (1.14-3.65)**
**Participates in activities in the neighbourhood where working**	138 (55.0)	83 (24.6)	**3.75 (2.64-5.33)**
**Collaborates with an NGO or civic entity**	151 (59.9)	133 (39.7)	**2.27 (1.62-3.17)**
**Freedom to organise timetable**	163 (65.7)	157 (47.7)	**2.10 (1.49-2.95)**
**Possibility colleagues will cover while performing CA**	99 (40.6)	79 (23.9)	**2.16 (1.51-3.11)**
**Can depend on colleagues to collaborate on CA**	215 (86.3)	222 (67.7)	**3.02 (1.96-4.63)**

Data from Spain 2009. Bold-face: Chi-squared $p \leq 0.05$

Due to missing data, 100 (16.8%) professionals were excluded from the multivariate analysis, leaving a total of 497. There were no significant differences between the excluded and included individuals. The final multivariate model (Table 4) shows that the following

For peer review only - http://bmjopen.bmj.com/site/about/guidelines.xhtml

factors were significantly associated with health-promoting community activities: profession (social workers do the most, followed by nurses and then physicians); specific training in community activities; motivation; social sensitivity (collaboration with NGOs and participation in neighbourhood activities); perception of support and coverage by colleagues while doing community activities; years working in the health care team; and being a nursing-student tutor.

For peer review only

For peer review only - http://bmjopen.bmj.com/site/about/guidelines.xhtml

Table 4. Multivariate model of performing health-promoting community activities (CA)

Variable	General model (n=497) OR (95% CI)
**Profession**	
Nurse	Ref.
Doctor	0.44 (0.24-0.82)
Paediatrician	1.55 (0.69-3.46)
Social worker	25.26 (2.53-251.9)
**Specific training in CA**	1.91 (1.17-3.11)
**Collaboration with NGO or civic entity**	1.91 (1.17-3.13)
**Possibility of work covered by colleagues**	1.80 (1.08-2.99)
**Support from colleagues in doing CA**	2.92 (1.49-5.73)
**Motivated to perform CA**	
Disagree or strongly disagree	Ref.
Agree	2.29 (1.28-4.08)
Strongly disagree	3.74 (1.85-7.54)
**Participates in activities in the neighbourhood**	3.06 (1.86-5.05)
**Years at health centre**†	1.06 (1.03-1.09)
**Nursing-student tutor**	1.96 (1.09-3.53)
**Model Validity**	**Value**
**Chi^2**	221.338
**Nagelkerke R squared**	.483

Discussion

Our study of the participation of health care workers in health-promoting activities in the community suggests that factors of the individual health care professional were more

For peer review only - http://bmjopen.bmj.com/site/about/guidelines.xhtml

important than those of the health care team or the community. In particular, the specific profession, attitudes, and opinions of an individual seemed to be crucial. Professional workload, which was one of the more mentioned factors by the professionals in others studies, does not have any impact when adjusted by other individual factors. These results confirms the frAC Project[29] hypothesis and those of other authors[21,28]

Our assessment of variables related with health care team participation in health promoting community activities suggests that team characteristics had greater impact than community characteristics. One exception was the socio-economic level of the community. This makes sense in the context of the Spanish health care system, which attempts to offer equitable care to people according to their needs and community interventions are important tools to reduce inequalities in health care[35]. It should be noted that we found no relationship between the level of social interaction in the community and the implementation of community activities, in contrast with the findings of other authors[16-17].

Our results showing the important role of nurses in health promoting community activities are consistent with those of other authors[13-14,17,36] who highlighted the role of nurses in the development of community activities. This result is expected because nurses usually receive more training in community care. Another crucial group is social workers. Previous research reported the importance of social workers[13], but their role in the community varies in different regions of Spain. Their presence seems fundamental for development of health-promoting community activities in regions where they have significant roles in the community. In Spain, community work is generally not provided as part of primary health care; instead, it is relegated to professional volunteers or specific programs. Thus, our results recognize the importance of work by nurses and social workers and points to the possibility that their roles could be modified or clarified. We also found that health centres that train undergraduate nurses have a lower level of community activities but that professional nurse-

For peer review only - http://bmjopen.bmj.com/site/about/guidelines.xhtml

trainers have a greater involvement in health promoting community activities. This apparent contradiction may be resolved by considering two factors. First, the variable at the health centre level included training in various disciplines (from nursing to physiotherapy) without distinction, even though there is clearly difference among these in communitarian tradition. Second, the need for increased training would make it difficult for the health team to engage in community activities although, among the cases trainers carried out more activities than others. Health care workers who worked for more years at a centre were also more likely to perform community activities. This may be because medium- and long-term projects require continuity and stability, and it is more difficult for professionals who continually change centres or have temporary contracts to participate in health promoting community activities.

The pressures of working in health care and the heavy work burden were the main arguments offered by professionals when asked about their low participation in health promotion and prevention activities[19,22,24-27]. Our bivariate analysis supported this relationship, although the multivariate analysis did not. This implies that the professionals' perception was incorrect. In fact, the pressure of working in health care and the heavy work burden are not associated with low participation in health promoting community activities.

Our results indicated the importance of the opinions and attitudes of professionals when performing health promoting community activities, in accordance with other studies[15,21-22]. In fact, all variables regarding the opinions and attitudes of health care workers were significantly different between cases and controls. This suggests that individuals who perform community activities have similar attitudes: they employ a biopsychosocial[12,37] practice model, are more oriented towards health promotion, show recognition of the need for citizen participation in health services, and are more left-leaning in ideology. We also found that social sensitivity was an important variable in the model for professionals. This variable measures involvement of the professional with the community beyond consultation work. It is

For peer review only - http://bmjopen.bmj.com/site/about/guidelines.xhtml

For peer review only

not clear how some practices, responsibility for which is stipulated in primary care legislation and which should be similar for all professionals to assure fairness within the system, can be affected by a professional's personal discourse. Most health care professionals that we interviewed – both cases and controls -- said that their community roles were not well defined. This suggests that greater priority should be given to develop health promoting community activities in primary health care, and to clarify the role and responsibility of every professional about this question.

We found that having support within the health care team is associated with performing health promoting community activities. This finding should be highlighted, because all the professionals in this study were selected from the centres that engage in community activities so that, presumably, all cases can count on some support from their centres. It may be that some professionals were unaware of what their colleagues were doing, or that there were barriers to working with particular colleagues. Regardless of the explanation, this result suggests that application of the teamwork model[38], an assumed key feature of primary health care, has certain weaknesses.

Strengths and limitations

This study had an observational design, so we could not definitively establish causal relationships. Similarly, our definition of "health promoting community activities" may be a limitation because this concept can be defined or perceived in different ways; we made an attempt to control for this by use of a definition generated by expert consensus, which we then transformed into an algorithm. The questionnaires we used were *ad hoc* and some of the data regarding team variables, such as degree of social interaction in the community and the relationship between professionals, were provided by team managers. This may have led to a bias in information gathering due to a lack of knowledge or other unknown factors. Some variables that we did not examine could have explained participation in community activities,

For peer review only - http://bmjopen.bmj.com/site/about/guidelines.xhtml

such as support from primary care management, the health care administration and the centres' own coordinators or nursing managers. It would be valuable to assess the effect of these variables in future studies of the effect of team activities and priorities.

The main strengths of this study are that it examined a facet of health care that is relatively unexplored; the study subjects were from 5 different regions of Spain; the sample size was large; and it considered many distinct factors simultaneously. Furthermore, our design of the second study, in which cases and controls were matched by teams, adjusted for the effect of team and community variables, so a multilevel analysis was not necessary.

Implications for health care practice

Health care administrators who want to develop health-promotion activities for the community must provide visible and formal support for community initiatives, define better the expected communitarian tasks of each sanitarian professional, and provide job stability for all participating workers. Specific training is also important for the formation of health-promoting community activities. It may seem that attitudes, social sensitivity, or personal opinions are not modifiable; however, in our opinion, it is possible to modify these factors during undergraduate education. So, community perspective and their associated responsibility should be included in undergraduate sanitary formation.

Conclusions

A key responsibility of primary health care is provision of health-promoting community activities. We found that the actual implementation of these activities depends more on the characteristics of individual professionals and on some characteristics of health care team or community than on his professional responsibility like primary health care workers. There were also significant differences among different types of professionals, and the contributions of nurses and social workers are fundamental. The pressures perceived by

For peer review only - http://bmjopen.bmj.com/site/about/guidelines.xhtml

members of the health care team were not associated with involvement in community activities.

Acknowledgements

We want to express thanks to Magdalena Esteva for her patient reading of the manuscript.

Competing interests

All authors have completed the ICMJE uniform disclosure form at www.icmje.org/coi_disclosure.pdf and declare: no financial relationships with any organisations that might have an interest in the submitted work in the previous three years; no other relationships or activities that could appear to have influenced the submitted work

Funding

This study was supported by a grant from the Carlos III Health Institute (PI07/90383; PI07/90925; PI07/90636). It also received support from the Health Promotion and Preventive Activities in Primary Health Care Research Network (IAPP network RD 06/0018/), the ERDF funds, and the Institut de Salut de Palma (IDISPA). SM was contracted with the aid of a grant to stabilise employment in health research from the Carlos III Health Institute and benefited from a grant for further studies from the same institution. All institutions are public and did not have any role in study design, analysis, or publication.

Contributors

SM was the lead investigator, and was responsible for conception and design of the study, analysing data and writing the manuscript. **MR** has the original idea of the study. **SM, JR, JLRG, IMG, CBBA,** and **LES**, coordinated the field work in everyone of the regions involved. SM, JR, MJM, EZO, CBBA, LES, CV, MLLBA, TPJ, DMPM, MLLS, JLRG, RBV, CSV, MRB, MCV, MBC, ESF, MBD, IMG, BBR, AAL, SBC, ACT, BOB, RMB, PMP, MEO, MJPJ, MR contributed to the design and development of the study, have read the manuscript critically, made contributions and approved the final version.

For peer review only - http://bmjopen.bmj.com/site/about/guidelines.xhtml

Data Sharing Statement: Questionnaires and anonymised data are available on demand.

For peer review only

For peer review only - http://bmjopen.bmj.com/site/about/guidelines.xhtml

References

1. World Health Organisation. **The Ottawa Charter for Health Promotion**, Health Promotion. 1986 1 (4): i-v.

2. World Health Organization. **Health Promotion Glossary.** WHO 1998. [http://apps.who.int/iris/bitstream/10665/64546/1/WHO_HPR_HEP_98.1.pdf]

3. World Health Organisation: **Declaration of Alma Ata.** *WHO* 1978. [http://www.who.int/hpr/NPH/docs/declaration_almaata.pdf]

4. World Health Organisation: **Declarations of Jakarta** WHO 1997. [http://www.who.int/hpr/NPH/docs/jakarta_declaration_sp.pdf]

5. March S, Torres E, Ramos M. **Adult community health-promoting interventions in primary health care: A systematic review.** *Prev Med* 2015; 76 (2015) S94–S104

6. Ley General de Sanidad 14/1986, de 25 de abril. Boletín Oficial del Estado,nº102,(29/4/1986).

7. Real Decreto 1030/2006 del 15 de septiembre por el que se establece la cartera de servicios comunes del Sistema Nacional de Salud y el procedimiento para su actualización. *Boletín Oficial del Estado*,nº222,(16-9-2006).

8. Guldan GS: **Obstacles to community Health promotion**. *Soc sci med* 1996, **5**:689-695.

9. Irigoyen J: **La participación comunitaria en salud: Una reformulación necesaria.** In: *Nuevas perspectivas en Atención Primaria de Salud.* Edited by Aranda. Madrid: Díaz de Santos; 1994:183-221.

10. Gérvas J, Pérez-Fernández M, Palomo-Cobos L, Pastor-Sánchez R: **Veinte años de reforma de la Atención Primaria en España. Valoración para un aprendizaje por acierto/error.** Madrid: Ministerio Sanidad y Consumo 2005. [http://equipocesca.org/new/wp-content/uploads/2010/08/20-anos-de-reforma-de-la-ap-en-espana.pdf]

For peer review only - http://bmjopen.bmj.com/site/about/guidelines.xhtml

11. Regato P, Domínguez J, Sancho M: **Coordinación sanitaria y abordaje comunitario en atención primaria**. In: *La Atención Primaria de Salud en España y en sus comunidades autónomas.* Coordinated by Navarro V, Martín-Zurro A. Barcelona: Semfyc; 2009:149-168.

12. Turabián JL, Pérez B: **Grandes misterios. ¿Puedes ver al monstruo del lago Ness?: El modelo biopsicosocial y las actividades comunitarias.** *Aten Primaria* 2007, **39**:261-4.

13. March S, Soler M, Miller F, et al: **Variabilidad en la implantación de las actividades comunitarias de promoción de la salud en España.** *An Sist Sanit Navar.* In press.

14. Sáez S, Marqués F, Pallarés J, Serrano J, Orrio C, Martínez V: **Perfil de los profesionales de la salud de Lleida respecto a la educación para la salud (EpS).** *Aten Primaria* 1999, **24**:157-161.

15. Pérez-Franco B, Turabián JL, Magaña E, et al: **Factores asociados a la participación comunitaria en la atención primaria de la provincia de Toledo.** *Aten primaria* 1993, **12**: 339-344.

16. MJ Aguilar Idáñez: **La participación comunitaria en salud: ¿Mito o realidad?** Madrid: Díaz de Santos; 2001.

17. López E, Forcada C, Miller Frederick: **Factores que facilitan y dificultan el desarrollo de los proyectos comunitarios. Estudio observacional de la red AUPA de centros de atención primaria de Cataluña.** *Aten primaria* 2010, **42**:218-25.

18. Duro JC: **Actitudes de los profesionales de atención primaria de Madrid sobre los grupos.** *Aten Primaria* 2005, **36**:71-7.

19. Díaz G: **Factores relacionados con la práctica de educación sanitaria por los médicos de Atención primaria de Galicia.** *Rev Esp Salud Pública* 2001, **75**:529-540.

For peer review only - http://bmjopen.bmj.com/site/about/guidelines.xhtml

20. Maheux B Dufort, F Lambert J et al: **The professional attitudes and clinical practices of men and women generalists.** *Can Fam Physician* 1989, **35**:59-63.

21. Maheux B, Pineault R, Lambert J et al: **Factors influencing physicians' preventive practices.** *Am J Prev Med* 1989, **5**:201-206.

22. Myrand A, Beehler G, Kuo C, Mahoney M: **Explaining the de-prioritization of primary prevention: Physicians' perceptions of their role in the delivery of primary care.** *BMC Public Health* 2003, **3**:15. doi:10.1186/1471-2458-3-15.

23. Walter U, Flick U, Neuber A, et al: **Putting prevention into practice: qualitative study of factors that inhibit and promote preventive care by general practitioners, with a focus on elderly patients.** *BMC Family practice* 2010, **11**:68. doi:10.1186/1471-2296-11-68

24. Puig ribera A, McKenna J, Riddoch C: **Attitudes and practices of physicians and nurses regarding physical activity promotion in the Catalan primary health-care system.** *Europ J Public Health* 2005, **15**:569–75.

25. Wilhemsson S, Lindberg L: **Health promotion: Facilitators and barriers perceived by district nurses.** *Int J Nurs Practice* 2009, **15**:156-163.

26. Rubio-Valera M, Pons-Vigues M, Martinez-Andres M et al. **Barriers and Facilitators for the implementation of primary prevention and health promotion activities in primary care: A Synthetesis through meta-etnography.** Plos one 2014;9:e89554. Doi: 10.1371/journal.pone.0089554.

27. Badertscher N, Rossi PO, Rieder A et al: **Attitudes, barriers and facilitators for health promotion in the elderly in primary care.** *Swiss Med Wkly* 2012;**142**:w13606.

28. Maheux B, Pineault L, Béland F et al: **Factors influencing physicians' orientation toward prevention.** *Am J Prev Med.* 1987, **3**:12-8.

For peer review only

29. March S, Ripoll J,Ruiz-Giménez JL, et al: **Observational study on factors related to health-promoting community activity development in primary care (frAC Project): a study protocol.** *BMJ Open* 2012, **2**. e001287 doi:10.1136/bmjopen-2012-001287.

30. March S, Jordán M, Montaner I et al. **¿Qué hacemos en el barrio? Descripción de las actividades comunitarias de promoción de la salud en atención primaria: Proyecto frAC.** Gac Sanit 2014; **28**:267-73.

31. March S, Ramos M, Soler M, Ruiz-JiménezJL, Miller F, Domínguez J, et al: **Revisión documental de experiencias de actividad comunitaria en atención primaria de salud.** *Aten Primaria* 2011, **43**:289-296.

32. T Martínez-cañavate: **Modelo multinivel explicativo de la utilización de las consultas de atención primaria en Andalucía**. *Phd tesis.* Granada university, faculty of medicine; 2007. [http://hera.ugr.es/tesisugr/16905234.pdf]

33. Mira JJ, Llinás G, Gil V, Orozco D, Palazón I, Vitaller J: **Validación de un instrumento para identificar estilos de práctica profesional del médico de atención primaria.** *Aten Primaria* 1998, **21**:14-22.

34. Rey del Castillo P: **Nota metodológica sobre los indicadores del barómetro.** *Rev Española de Investigaciones Sociológicas* 2004, **108**:151-78.

35. Comisión para reducir las desigualdades en salud en España: **Propuesta de políticas e intervenciones para reducir las desigualdades en salud en Espanya**. *Gac Sanit* 2012. doi:10.1016/j.gaceta.2011.07.024.

36. Hogg W, Dahrouge S. Russell G et al: **Health promotion activity in primary care: performance of models and associated factors.** *Open medicine* 2008, **3**:165-73.

37. Engel GL: **The need for a new medical model: a challenge to biomedicine.** Science 1977, **196**:129–36.

For peer review only - http://bmjopen.bmj.com/site/about/guidelines.xhtml

38. Grumbach K, Bodenheimer T: **Can Health Care teams improve Primary Care practice?** JAMA 2004, **291**:1246-51.

For peer review only

For peer review only - http://bmjopen.bmj.com/site/about/guidelines.xhtml

STROBE Statement—Checklist of items that should be included in reports of ***case-control studies***

	Item No	Recommendation
**Title and abstract**	1	(*a*) Indicate the study's design with a commonly used term in the title or the abstract<br>OK. p1.
		(*b*) Provide in the abstract an informative and balanced summary of what was done and what was found<br>OK. p2
**Introduction**		
Background/rationale	2	Explain the scientific background and rationale for the investigation being reported<br>OK. p4-5
Objectives	3	State specific objectives, including any prespecified hypotheses<br>OK. p5
**Methods**		
Study design	4	Present key elements of study design early in the paper<br>OK. p5
Setting	5	Describe the setting, locations, and relevant dates, including periods of recruitment, exposure, follow-up, and data collection<br>OK. p6
Participants	6	(*a*) Give the eligibility criteria, and the sources and methods of case ascertainment and control selection. Give the rationale for the choice of cases and controls<br>OK. p6
		(*b*) For matched studies, give matching criteria and the number of controls per case<br>OK. p6.
Variables	7	Clearly define all outcomes, exposures, predictors, potential confounders, and effect modifiers. Give diagnostic criteria, if applicable<br>OK. p6-8.
Data sources/ measurement	8*	For each variable of interest, give sources of data and details of methods of assessment (measurement). Describe comparability of assessment methods if there is more than one group<br>OK. p6-8
Bias	9	Describe any efforts to address potential sources of bias<br>OK. p17-18
Study size	10	Explain how the study size was arrived at<br>OK. p8
Quantitative variables	11	Explain how quantitative variables were handled in the analyses. If applicable, describe which groupings were chosen and why<br>OK. p6-8
Statistical methods	12	(*a*) Describe all statistical methods, including those used to control for confounding<br>OK. p8-9
		(*b*) Describe any methods used to examine subgroups and interactions<br>OK. p8-9
		(*c*) Explain how missing data were addressed<br>OK. p8-9
		(*d*) If applicable, explain how matching of cases and controls was addressed<br>Not applicable
		(*e*) Describe any sensitivity analyses

For peer review only

		Not applicable
**Results**		
Participants	13*	(a) Report numbers of individuals at each stage of study—eg numbers potentially eligible, examined for eligibility, confirmed eligible, included in the study, completing follow-up, and analysed<br>OK. p11
		(b) Give reasons for non-participation at each stage<br>OK. p11
		(c) Consider use of a flow diagram<br>OK. It is not necessary.
Descriptive data	14*	(a) Give characteristics of study participants (eg demographic, clinical, social) and information on exposures and potential confounders<br>OK. Tables 1 and 2.
		(b) Indicate number of participants with missing data for each variable of interest<br>OK. Tables 1-3.
Outcome data	15*	Report numbers in each exposure category, or summary measures of exposure<br>OK. Tables 1-3.
Main results	16	(*a*) Give unadjusted estimates and, if applicable, confounder-adjusted estimates and their precision (eg, 95% confidence interval). Make clear which confounders were adjusted for and why they were included<br>OK. Tables 1-3.
		(*b*) Report category boundaries when continuous variables were categorized<br>OK. Tables 1-3.
		(*c*) If relevant, consider translating estimates of relative risk into absolute risk for a meaningful time period<br>OK. It is not necessary.

For peer review only

For peer review only - http://bmjopen.bmj.com/site/about/guidelines.xhtml

Other analyses	17	Report other analyses done—eg analyses of subgroups and interactions, and sensitivity analyses<br>OK. p8-9.
**Discussion**		
Key results	18	Summarise key results with reference to study objectives<br>OK. p14-15.
Limitations	19	Discuss limitations of the study, taking into account sources of potential bias or imprecision. Discuss both direction and magnitude of any potential bias<br>OK. p17-18.
Interpretation	20	Give a cautious overall interpretation of results considering objectives, limitations, multiplicity of analyses, results from similar studies, and other relevant evidence<br>OK. p15-18.
Generalisability	21	Discuss the generalisability (external validity) of the study results<br>OK. p17-18
**Other information**		
Funding	22	Give the source of funding and the role of the funders for the present study and, if applicable, for the original study on which the present article is based<br>OK. p19

*Give information separately for cases and controls.

Note: An Explanation and Elaboration article discusses each checklist item and gives methodological background and published examples of transparent reporting. The STROBE checklist is best used in conjunction with this article (freely available on the Web sites of PLoS Medicine at http://www.plosmedicine.org/, Annals of Internal Medicine at http://www.annals.org/, and Epidemiology at http://www.epidem.com/). Information on the STROBE Initiative is available at http://www.strobe-statement.org.

For peer review only

BMJ Open

Factors related to the development of health-promoting community activities in Spanish primary health care: Two case-control studies

Journal:	*BMJ Open*
Manuscript ID	bmjopen-2017-015934.R3
Article Type:	Research
Date Submitted by the Author:	30-Jun-2017
Complete List of Authors:	March, Sebastià; Baleares Health services-IbSalut, Primary Care Research Unit of Mallorca; Instituto de investigación en salud de Palma (IDISPA)<br>Ripoll, Joana; Baleares Health services-IbSalut, Primary Care Research Unit of Mallorca; Baleares Health services-IbSalut, Primary Care Research Unit of Mallorca<br>Jordan Martin, Matilde Jordan Martin; Estrecho de Correa Primary health center, Madrid Health Services<br>Zabaleta del Olmo, Nieves; Fundacio Jordi Gol i Gurina, IDIAP<br>Benedé Azagra, Carmen Belén; Aragon Health Services, Oliver Primary health center<br>Elizalde Soto, Lázaro; Gobierno de Navarra Departamento de Salud, public health institute; Public Health and epidemiology CIBER,<br>Vidal, Mª Clara; Baleares Health services-IbSalut, Primary Care Research Unit of Mallorca; Instituto de investigación en salud de Palma (IDISPA)<br>Bauzà Amengual, M. de Lluc; Balearic Islands University,<br>Planas Juan, Trinidad; Baleares Health services-IbSalut, Son Gotleu Primary health care center<br>Maria Pèrez, Damiana Maria Pèrez; Son Gotleu Primary health center, Baleares Health services-IbSalut<br>Llull Sarralde, Micaela; Baleares Health services-IbSalut, San Agustí Primary health care center<br>Ruiz-Giménez, Juan Luís; Madrid Health Services, Madrid, Vicente Soldevilla Primary health care center<br>Bajo Viñas, Rosa; Madrid Health care services, Loeches Primary health care center<br>Solano Villarubia, Carmen Solano Villarubia; madrid Health care services, DA Suroeste Health center<br>Rodriguez Bajo, Maria<br>Cordoba Victoria, Manuela Cordoba Victoria; SOMAMFyC<br>Badia Capdevila, Marta; Institut Catala De La Salut, El carmel Health center<br>Serrano Ferrandez, Elena; Institut Catala De La Salut, El carmel health center<br>Bosom Diumenjo, Maria; Institut Catala De La Salut, Sant Rafel Primary Health Center<br>Montaner-Gomis, Isabel; Institut Catala De La Salut, El Carmel Health center<br>Bolibar-Ribas, Buenaventura; Fundacio Jordi Gol i Gurina, IDIAP<br>Antoñanzas Lombarte, Angel; Aragon Health services, Delicias del Sur health center

	Bregel Cotaina, Samantha; Instituto Aragones de Ciencias de la Salud, Primary health care research unit<br>Calvo Tocado, Ana; Zaragoza Public Health provincial subdirection<br>Olivan Blázquez, Barbara; Instituto Aragones de Ciencias de la Salud, primary health care research unit<br>Magallon Botaya, Rosa; Aragon Health services, Arrabal health center<br>Marín Palacios, Pilar Marín Palacios; Gobierno de Navarra Departamento de Salud, Public health institute<br>Echauri Ozcoidi, Margarita; Gobierno de Navarra Departamento de Salud, public health institute<br>Perez-Jarauta, Mª Jose Perez-Jarauta; Gobierno de Navarra Departamento de Salud, public health institute<br>Llobera, Joan; Baleares Health services-IbSalut, 1. Primary Care Research Unit of Mallorca; Instituto de investigación en salud de Palma (IDISPA)<br>Ramos, Maria; Govern de les Illes Balears Conselleria de Salut Familia i Benestar Social, Direcció General de salut Pública i Participació; Instituto de investigación en salud de Palma (IDISPA)
**Primary Subject Heading**:	General practice / Family practice
Secondary Subject Heading:	Health services research, Public health
Keywords:	Health promotion, Primary health care, Health services research, Community health, Health team, Health professionals

SCHOLARONE™ Manuscripts

For peer review only

Factors related to the development of health-promoting community activities in Spanish primary health care: Two case-control studies

Sebastià March[1], Joana Ripoll[1], Matilde Jordan Martin[2], Edurne Zabaleta-del-Olmo[3][3], Carmen Belén Benedé Azagra[4], Lázaro Elizalde Soto[5], Mª Clara Vidal[1], María de Lluc Bauzà Amengual[6], Trinidad Planas Juan[7], Damiana Maria Pérez Mariano[7], Micaela Llull Sarralde[8], Juan Luís Ruiz-Giménez[9], Rosa Bajo Viñas[10] , Carmen Solano Villarubia[11], Maria Rodriguez Bajo[12], Manuela Cordoba Victoria[13], Marta Badia Capdevila[3], Elena Serrano Ferrandez[3], Maria Bosom Diumenjo[14], Isabel Montaner Gomis[15], Bonaventura Bolíbar-Ribas [3], Angel Antoñanzas Lombarte[16], Samantha Bregel Cotaina[17], Ana Calvo Tocado[18], Barbara Olivan Blázquez[17], Rosa Magallón Botaya[19], Pilar Marín Palacios[5], Margarita Echauri Ozcoidi[5], María Jose Perez-Jarauta[5], Joan Llobera[1], Maria Ramos[20].

1. Primary Care Research Unit of Mallorca, Baleares Health Services-IbSalut, Palma de Mallorca, Spain.
2. Estrecho de Correa Primary Health Center, Madrid Health Services, Madrid, Spain.
3. Institut Universitari d'Investigació en Atenció Primària Jordi Gol (IDIAP Jordi Gol), Barcelona, Spain..
4. Oliver Primary Health Center, Aragon Health Services, Zaragoza, Spain.
5. Navarra Public Health Institute, and Public Health and Epidemiology CIBER, Pamplona, Spain.
6. Balearic Islands University, Palma de Mallorca, Spain.
7. Son Gotleu Primary Health Center, Baleares Health Services-IbSalut, Palma de Mallorca, Spain.
8. San Agustí Primary Health Center, Baleares Health Services-IbSalut, Palma de Mallorca, Spain.
9. Vicente Soldevilla Primary Health Center, Madrid Health Services, Madrid, Spain.
10. Loeches Primary Health Center, Madrid Health Services, Madrid, Spain.
11. DA Sureste Primary Health Center, Madrid Health Services, Madrid, Spain.
12. Freelance, Madrid, Spain
13. SOMAMFYC, Madrid, Spain
14. Sant Rafael Primary Health Center, Àmbit d'Atenció Primària Barcelona-Ciutat, Institut Català de la Salut, Barcelona, Spain.
15. El Carmel Primary Health Center, Àmbit d'Atenció Primària Barcelona-Ciutat, Institut Català de la Salut, Barcelona, Spain.
16. Delicias del Sur Primary Health Center, Aragon Health Services, Zaragoza, Spain.
17. Primary Care Research Unit, Aragon, Spain
18. Zaragoza Public Health department, Zaragoza, Spain.
19. Arrabal Primary Health Center, Aragon Health Services, Zaragoza, Spain.
20. Public Health Department, Balearic Islands Health Department, Spain.

Corresponding author:
Sebastià March
E-mail: smarch@ibsalut.caib.es,

For peer review only - http://bmjopen.bmj.com/site/about/guidelines.xhtml

Abstract

Objective: Spanish primary health care teams have the responsibility of performing health-promoting community activities, although such activities are not widespread. Our aim was to identify the factors related to participation in those activities.

Design: Two case-control studies. **Setting:** Performed in primary care of 5 Spanish regions. **Subjects:** In the first study, cases were teams that performed health promoting community activities and controls were those that did not. In the second study (on case teams from the first study), cases were professionals who developed these activities and controls were those who did not. **Main outcome measures:** Team, professional, and community characteristics collected through questionnaires (team managers/professionals) and from secondary sources.

Results: The first study examined 203 teams (103 cases, 100 controls). Adjusted factors associated with performing community activities were percentage of nurses (OR=1.07, 95%CI =1.01-1.14), community socio-economic status (higher *vs.* lower OR =2.16, 95%CI=1.18-3.95), and performing undergraduate training (OR=0.44, 95%CI=0.21-0.93). In the second study, 597 professionals responded (254 cases, 343 controls). Adjusted factors were professional classification (physicians do fewer activities than nurses and social workers do more), training in community activities (OR=1.9, 95%CI =1.2-3.1), team support (OR=2.9, 95%CI =1.5-5.7), seniority (OR=1.06, 95%CI=1.03-1.09), nursing tutor (OR =2.0, 95%CI =1.1-3.5), motivation (OR =3.7, 95%CI=1.8-7.5), collaboration with non-governmental organizations (OR=1.9, 95%CI=1.2-3.1), and participation in neighbourhood activities (OR=3.1, 95%CI=1.9-5.1).

Conclusions: Professional personal characteristics, as social sensitivity, profession, to feel team support, or motivation, have influence in performing health-promoting community

For peer review only - http://bmjopen.bmj.com/site/about/guidelines.xhtml

activities. In contrast to the opinion expressed by many professionals, workload is not related to performance of health-promoting community activities.

Keywords: Health promotion, Primary health care, Health services research, Community health, Health team, health professionals

Strengths and limitations of this study

- This study examined a topic relatively unexplored, community health promotion by primary health care teams.

- A case-control approach was chosen, where cases were subjects (professionals or teams) who were developing health promoting community activities, and controls those who did not.

- Design allows explor many distinct factors simultaneously, from different sources, and in two levels: Health team and professional.

- An operative definition of community activities was used, which recognizes the heterogeneity of health promotion interventions developed with the community in Spanish primary health care settings.

- Because of its observational design, there are limitations to establishing causal inferences.

For peer review only

Introduction

The Ottawa Charter for Health Promotion[1] defined Health promotion as the process of enabling people to increase control over, and to improve, their health. This definition implies the importance of empowering people[2] to take control over what determines their health. One tool to work in this direction are health promoting community activities. These can come in a variety of forms, from walking groups in a neighbourhood to holding meetings with local authorities, schools or civil associations to develop specific health programs. Experts at numerous international conferences have agreed on the importance of health-promoting community activities for improving public health[1,3-4], and there is some evidence that these activities are effective[5].

Since the implementation of health care reform in Spain during the 1980's[6-7], primary health care teams have had responsibility for carrying out health promotion activities. These teams, located in health care centres throughout the country, are composed of physicians, community nurses, paediatricians, midwives, social workers, and other health care professionals (gynaecologists, psychologists, and psychiatrists) in a system that offers almost-universal and free health care. These conditions are ideal for an interdisciplinary approach to encourage health promoting community activities. However, as in other countries[8], implementation of these interventions differs greatly among the different health care teams[9-11], and relatively few professionals are involved[11-13].

There has been limited studies of the factors related to the development of health-promoting interventions in the community. These factors may be broadly classified as those related to the community, to the team, or to the individual professionals. With regard to community factors, some studies indicated that the rural/urban environment[14], size of the municipality[15], level of social interaction within the community[15-17], and presence of a focal point for participation in community health initiatives[15-16] influence the practice of health

For peer review only - http://bmjopen.bmj.com/site/about/guidelines.xhtml

promoting community activities. The primary health care team factors include job satisfaction[18], management of time and setting within the center[14], composition and organization of the team[15-16], years of team operation[15], intra-team support[17], and work burden[8,19]. Studies of professionals have highlighted sex[19-21], age[15,20], professional status[14], specific training[22-25], motivation[17-18], and the model applied (biomedical or psychosocial)[18]. The attitudes and beliefs of the professionals, such as trust in the effectiveness of preventive actions[18,22,25-27], self-reported efficacy in carrying out activities[8,22-23], or to support community participation[15,21] are also important. An additional element is professionals' social sensitivity, as evaluated through their personal political leanings[28]. According to professionals themselves, the most important factor is the lack of time available to assume the care burden[19,22,24-27]. All these features have been observed separately, mostly in descriptive studies.

This study presents some of the results of the Factors Related to Health-Promoting Community Activity Development in Primary Care (frAC) Project, whose complete methodology and aims were described elsewhere[29]. The purpose of this study is to identify primary health care team and community factors related to implementation of health promoting community activities in primary care and factors that may explain why only some professionals within a team participate in those community activities.

Methods

Two case-control studies were performed. In the first, the cases were the teams that performed health promoting community activities and the controls were those that did not. The second study examined only teams that implemented these community activities to adjust for community/team factors; in this second analysis the cases were professionals who developed these activities and the controls were those who did not. The study was set in

For peer review only - http://bmjopen.bmj.com/site/about/guidelines.xhtml

For peer review only

primary health care centers in five regions of Spain (Balearic Islands, Catalonia, Aragon, Madrid, and Navarra). Data collection was carried out between 2009-2010.

Selection of cases and controls

We used a conceptual definition of health promoting community activity reached in a previous study [30-31]. This was converted into an algorithm for use in this study[29]: this activity had to be a non-isolated activity carried out in the previous year, in which the professional participated on behalf of the health centre. Furthermore, it had to have involvement and active participation of the community, or the population had to have the capacity to influence the intervention, or it was a cross-sector activity involving collaboration with entities outside of health care (*e.g.* education, social services). Health preventive interventions which does not have a health promotion approach were excluded.

We telephoned all the health centres in the research areas asking if they had participated in health promoting community activities during the last year. If they did, we contacted a participant of the activity and gave him/her a questionnaire to confirm that the activity met our inclusion criteria. For the first study, health teams that participated in at least one community activity in the last year were selected as cases, and controls were those who had not participated. For the second study, we selected all professionals who participated in the implementation of those community activities as cases and professionals who did not participate as controls. In every health team we selected at least one control for every case.

Variables

Information was gathered through three distinct questionnaires (available on request) that were given to the community, the team, and the professionals[29]. Description of health promoting community activities are described elsewhere[30].

The data collected from the community (from from questionnaires to centre managers and secondary sources) were: demographic composition; socio-economic level; degree of social

For peer review only - http://bmjopen.bmj.com/site/about/guidelines.xhtml

interaction; health centre setting; geographical dispersion based on an ordinal variable used by the National Health System for resource allocation (from G1 [less dispersed] to G4 [more dispersed]); and existence of health boards (community participation agencies described in health legislation)[16].

The data collected from the team (collected from secondary sources and questionnaires to centre managers) were: percentages of different professionals (nurses, physicians, and paediatricians); year of opening; population of service area; professional/population and doctor/nurse ratios; availability of a space in the centre to carry out group activities and presence of a team member responsible for health education; collaboration with the area health board where applicable; training of resident physicians; nurses and nursing or physiotherapy students; evaluation of professional relationships between distinct professional disciplines (based on a Likert-type scale ranging from 1 [poor] to 5 [excellent]).

The data collected from the professionals (from individual self-administrated questionnaires) were: sex; age; profession; working situation; health care burden; size of quota allocated; average number and duration of consultations per day at the centre or at home; tutoring; participation in research or specific training in health promotion in the previous five years; years since graduation; years worked in primary care and as a member of the current health care team; autonomy to organize scheduling; arrangements with colleagues to cover health care tasks while carrying out community activities; support of fellow team members in conducting these activities; working atmosphere; job satisfaction; and self-assessed efficacy in conducting community work (based on a Likert-type scale ranging from 1 [poor] to 5 [excellent]).

Opinions and attitudes were measured by responses to various statements developed by the research team, using a Likert-type scale with four options ranging from "strongly

For peer review only - http://bmjopen.bmj.com/site/about/guidelines.xhtml

agree" to "strongly disagree". The statements dealt with trust in the effectiveness of the health promotion community activities, motivation to carry out these activities, citizen participation in health care decision-making, need to strengthen agencies involved in citizen participation in health care, responsibility for primary care in the community, definition of professional role, link between health education and community participation, and the effectiveness of the boards in driving community activities. A four-item validated scale was used to evaluate each professional's clinical practice style (biomedical-psychosocial)[32-33] by assessing the attitude towards the demands of psychosocial and clinical care at work. Finally, social sensitivity data were collected through proxy variables: participation during the previous twelve months in activities in the neighbourhood where the professional works; involvement in any NGO or civil association; and political tendency (assessed through a visual scale adapted from the Centre for Sociological Research[34] Spanish monthly barometer).

Calculation of sample size

For the first study, we calculated that at least 91 teams would be needed in each group based on an OR of 2.5, an alpha risk of 0.05, a beta of 0.2, and an expected proportion of 0.5 in the controls. For the second study, we calculated that at least 222 professionals would be needed in each group based on an OR of 1.75, an alpha risk of 0.05, a beta of 0.2, and an expected proportion of 0.5 in the controls.

Data analysis and management

A descriptive analysis of all variables was carried out to compare their distributions in each group. Categorical variables are expressed as percentages and continuous variables as means with 95% confidence intervals (95% CIs) or as medians and percentiles depending on the distribution. Main variable was, for both studies, to be case or control. The relationship with the main variable was assessed using the chi-squared test for categorical variables and Student's *t*-test and the Mann-Whitney U non-parametric test for continuous

For peer review only - http://bmjopen.bmj.com/site/about/guidelines.xhtml

variables. The strength of an association was expressed through unadjusted OR and its 95% CI.

We also performed a logistic regression analysis to calculate adjusted ORs. We constructed a first model using each variable whose *p*-value was less than 0.2 in the bivariate analysis. From this model, we tested and compared variations using the maximum likelihood method, eliminating variables that had *p*-values less than 0.05 in the Wald test. The collinearity of the variables was examined and interactions tested. We established a different model for the health care teams and for the professionals. All analyses were conducted using SPSS ver. 14 and Epidat ver. 3.1 software.

Ethical aspects

The study was approved by the Research Commissions in all the participant regions and by the Research Ethics Committee in Madrid (Area 1) and the University Research Institute in Primary Care (IDIAP) Jordi Gol in Barcelona. The questionnaire was completed anonymously by the professionals, with the identity of individuals and centres coded to ensure blinding of the researchers.

Results

Factors related to the health care team

We examined 203 health care teams (103 cases [50.7%] and 100 controls) in the first study. Table 1 shows the results of the initial bivariate analysis. The variables significantly related to team involvement in community activities were the socioeconomic level of the community, collaboration on training in physiotherapy or nursing, participation in a health board, percentage of nurses in the team, patient/physician ratio, and patient/nurse ratio.

For peer review only - http://bmjopen.bmj.com/site/about/guidelines.xhtml

Table 1. Relationship of community and primary health care team (PHT) characteristics with performance of health-promoting activities in the community.

Nominal Variables	PHT cases N (%)	PHT controls N (%)	Unadjusted OR (95% CI)
**Health Care setting**			
Urban	73 (71.6)	68 (68.0)	Ref.
Rural	29 (28.4)	32 (32.0)	0.84 (0.46-1.54)
**Socioeconomic level**			
High and medium-high	52 (51)	65 (65)	1
Medium-low and low	50 (49)	35 (35)	**1.79 (1.01-3.14)**
**Degree of social interaction**			
High	22 (23.9)	23 (26.1)	Ref.
Medium	43 (46.7)	35 (39.8)	1.28 (0.61-2.68)
Low	27 (29.3)	30 (34.1)	0.94 (0.43-2.06)
**Population of municipality**			
>1 million	47 (45.6)	48(48)	Ref.
500,000-1 million	7 (12.6)	7 (7)	1.90 (0.70-5.18)
10,000-500,000	22 (21.4)	33 (33)	0.68 (0.35-1.33)
<10,000	21 (20.4)	12 (12)	1.79 (0.79-4.04)
**Degree of geographical dispersion**			
G1 (less dispersed)	62 (66.7)	60 (63.8)	Ref.
G2	11 (11.8)	17 (18.1)	0.63 (0.27-1.45)
G3	9 (9.7)	11 (11.7)	0.79 (0.31-2.05)
G4 (more dispersed)	11 (11.8)	6(6.4)	1.77 (0.67-5.10)
**Active health board**	67 (66.3)	60 (61.9)	1.21 (0.68-2.17)
**Training centre**			
Resident Doctors	35 (34.7)	34 (34.3)	1.01 (0.56-1.82)
Resident Nurses	23 (23.5)	29 (30.2)	0.71 (0.37-1.34)
Nursing/physiotherapy students	73 (72.3)	84 (85.7)	**0.43 (0.21-0.89)**
**Space for group activities**	83 (81.4)	79 (79)	1.16 (0.58-2.32)
**Health education manager**	45 (44.1)	46 (46)	0.93 (0.53-1.61)
**Participation in health board**	56 (83.6)	38 (63.3)	**2.95 (1.28-6.78)**
**Continuous variables**	**Mean (95% CI)**		**p[*]**
PHT operational years	14.3 (13-15.2)	12.9 (11.7-14.1)	.075
Total No. professionals	36.4 (33.1-39.6)	37.5 (34-41.1)	.486
% nurses	35.4 (34.5-36.4)	33.5 (32.4-34.5)	**.010**
% paediatricians	7.0 (6.3-7.7)	7.4 (6.7-8.1)	.450
Population covered by PHT	20031 (18059-22002)	21770.4 (19530-24010)	.247
	**Median (IQR)**		**p[**]**
% doctors	33.3 (29.5-38.3)	33.3 (29.9-36.4)	.532
% non-EU immigrants	11.1 (5.8-16.2)	11.1 (8.6-15.7)	.690
% population <15 years	13.0 (11.6-14.7)	12.9 (11.3-14.4)	.352
% population >65 years	17.8 (15.0-21.8)	18.9 (13.8-21.5)	.976
% population women	51 (49.5-53)	51.2 (50.3-53.1)	.403
Population ratio registered/professional	524 (444-658)	578 (482-669)	.122
Population ratio registered/doctor	1659 (1454-1905)	1734 (1533-2040)	**.034**
Population ratio registered/nurse	1508 (1284-1847)	1723 (1434-2057)	**.008**
Doctor/nurse ratio	.94 (.86-1.09)	1 (.89-1.14)	.085
**Professional relationships**			
Doctor::nurse	4 (3-4)	4 (3-4)	.376
Doctor::social worker	4 (3-5)	4 (3-4)	.978
Nurse::social worker	4 (3-5)	4 (3-4)	.845

Data from Spain 2009. For dichotomous variables, the reference value is always absence of the variable.[*] Student's t-test P. [**] Mann-Whitney U test P. Bold-face: $p \leq 0.05$.

For peer review only

For peer review only - http://bmjopen.bmj.com/site/about/guidelines.xhtml

Eleven teams (5.0%) were excluded from the logistic regression analysis due to missing data. The 3 variables remaining in the final model were percentage of nurses in the team (OR = 1.07 for every 1% increase; 95% CI = 1.01-1.14); socio-economic level of the community (high and medium-high *vs.* low and medium-low OR = 2.16; 95% CI = 1.18-3.95); and having undergraduate training at the health care centre (OR = 0.44; 95% CI = 0.21-0.93). Nagelkerke's R squared was 0.11.

Factors related to professionals

A total of 597 professionals responded to the questionnaire, 254 (42.4%) cases and 343 (57.5%) controls. Refused to participate 96 professionals (62 cases and 34 controls). Participants were 298 nurses (52.4%), 191 doctors (33.6%), 58 paediatricians (10.2%), and 22 social workers (3.8%). Tables 2 and 3 show the results of the bivariate descriptive analysis. Table 2 shows that the variables related to involvement in health promoting community activities were sex, profession, training, self-confidence, and work situation. Also years of experience, age, workload, practice style and political orientation seems to be related. Table 3 summarizes the responses of the professionals' opinions and attitudes to health promotion, community participation, and social sensitivity.

For peer review only

Table 2. Relationship of professional variables with performance of health-promoting community activities (CA).

Nominal Variables	Cases N (%)	Controls N (%)	Unadjusted OR (95% CI)*
**Sex**			
Male	41 (16.4)	84 (25.3)	Ref.
Female	209 (83.6)	248 (74.7)	**1.72 (1.13-2.61)**
**Profession**			
Nurse	156 (64.7)	142 (43.3)	Ref.
Doctor	40 (16.6)	151 (46)	**0.24 (0.16-0.36)**
Paediatrician	24 (10)	34 (10.4)	0.64 (0.36-1.3)
Social worker	21 (8.7)	1 (0.3)	**19.11 (2.53-143.9)**
**Specialized General practitioner**	22 (55)	91 (60.3)	0.81 (0.39-1.62)
**Tutor of Resident doctors**	10 (25)	37 (24.5)	1.02 (0.45-2.3)
**Nursing tutors**	93 (59.6)	58 (40.8)	**2.13 (1.34-3.39)**
**Work situation**			
Permanent	204 (80.3)	238 (70)	Ref.
Temporary contracts	40 (15.7)	74 (21.8)	**0.63 (0.41-0.96)**
Other	10 (3.9)	28 (8.2)	**0.41 (0.19-0.87)**
**Participation in research projects in last 5 years**	115 (47.7)	147 (43.9)	1.16 (0.83-1.62)
**Specific training in CA**	142 (58.2)	104 (31.7)	**2.99 (2.12-4.23)**
**Job satisfaction**			
None/little	18 (7.1)	34 (10)	Ref.
Average	64 (25.3)	129 (38.1)	0.93 (0.49-1.78)
Quite a lot/a lot	171 (67.5)	176 (51.9)	1.83 (0.99-3.37)
**Working environment satisfaction**			
None/little	14 (5.6)	33 (9.8)	Ref.
Average	82 (32.5)	103 (30.5)	1.87 (0.94-3.73)
Quite a lot/a lot	156 (61.9)	202 (59.8)	1.82 (0.94-3.51)
**Self-confidence to carry out CA**			
None/little	31 (12.3)	72 (21.6)	Ref.
Average	81 (32.1)	154 (46.2)	1.22 (0.74-2.01)
Quite a lot/a lot	140 (55.5)	107 (32.1)	**3.03 (1.86-4.96)**
**Continuous Variables**	**Median (p25-p75)**		**p***
**Age**	49 (42-54)	48 (39-53)	**.048**
**Years qualified**	25 (18-31)	24 (15-29)	**.024**
**Years in primary care**	19 (11-23)	17 (10-23)	.552
**Years in current health centre**	11 (4-18)	7 (2-15)	**.000**
**Size of assigned quota**	1737 (1300-2043)	1600 (1300-1900)	**.026**
**Average daily consultations**	20 (15-30)	30 (20-35)	**.000**
**Average daily home visits**	1 (0.5-3)	1 (1-2)	.932
**Average consultation time**	10 (10-15)	10 (7-12)	**.000**
**Average home-visit time**	25 (20-30)	20 (15-30)	**.026**
**Biomedical practice style (+) Psychosocial (-)**	8 (6-9)	8 (7-9)	**.040**
**Political orientation: Left (-) right (+).**	2 (1-5)	3 (2-5)	**.026**

Data from Spain 2009. For dichotomous variables, the reference value is always absence of the variable. Bold-face: Chi-squared p ≤less than 0.05. * Mann-Whitney U test.

For peer review only

For peer review only - http://bmjopen.bmj.com/site/about/guidelines.xhtml

Table 3. Professional attitudes in cases and controls regarding health-promoting community activities (CA).

Variable	Cases N (%)	Controls N (%)	Unadjusted OR (IC 95%)
**Confidence in CA efficacy**			
Disagree/strongly disagree	9 (3.6)	19 (5.7)	Ref.
Agree	110 (43.8)	196 (59.0)	1.18 (0.51-2.71)
Strongly agree	132 (52.6)	117 (35.2)	**2.38 (1.03-5.46)**
**Motivated to carry out CA**			
Disagree- strongly disagree	44 (17.5)	149 (45.4)	Ref.
Agree	113 (45.0)	131 (39.9)	**2.92 (1.91-4.44)**
Strongly agree	94 (37.5)	48 (14.6)	**6.63 (4.08-10.7)**
**Citizen participation in health**			
Disagree/strongly disagree	23 (9.2)	46 (14.0)	Ref.
Agree	122 (49.0)	191 (58.1)	1.27 (0.73-2.21)
Strongly agree	104 (41.8)	92 (28.0)	**2.26 (1.27-4.01)**
**Citizen participation in health services**			
Disagree/strongly disagree	24 (9.6)	48 (14.5)	Ref.
Agree	109 (43.6)	198 (59.8)	1.10 (0.64-1.89)
Strongly agree	117 (46.8)	85 (25.7)	**2.75 (1.56-4.83)**
**Professionals' role in the community is well-defined**			
Disagree/strongly disagree	126 (51)	223 (66.8)	Ref.
Agree	103 (41.7)	97 (29)	**1.87 (1.32-2.67)**
Strongly agree	18 (7.3)	14 (4.2)	**2.27 (1.09-4.73)**
**Health education and community participation are closely linked**			
Disagree/strongly disagree	20 (8.1)	39 (11.7)	Ref.
Agree	128 (51.6)	194 (58.4)	1.28 (0.71-2.31)
Strongly disagree	100 (40.3)	99 (29.8)	**1.97 (1.07-3.61)**
**The health board drives CA**			
Disagree/strongly disagree	80 (33.8)	125 (39.8)	Ref.
Agree	123 (51.9)	163 (51.9)	1.17 (0.81-1.69)
Strongly agree	34 (14.3)	26 (8.3)	**2.04 (1.14-3.65)**
**Participates in activities in the neighbourhood where working**	138 (55.0)	83 (24.6)	**3.75 (2.64-5.33)**
**Collaborates with an NGO or civic entity**	151 (59.9)	133 (39.7)	**2.27 (1.62-3.17)**
**Freedom to organise timetable**	163 (65.7)	157 (47.7)	**2.10 (1.49-2.95)**
**Possibility colleagues will cover while performing CA**	99 (40.6)	79 (23.9)	**2.16 (1.51-3.11)**
**Can depend on colleagues to collaborate on CA**	215 (86.3)	222 (67.7)	**3.02 (1.96-4.63)**

Data from Spain 2009. Bold-face: Chi-squared $p \leq 0.05$

Due to missing data, 100 (16.8%) professionals were excluded from the multivariate analysis, leaving a total of 497. There were no significant differences between the excluded and included individuals. The final multivariate model (Table 4) shows that the following

For peer review only - http://bmjopen.bmj.com/site/about/guidelines.xhtml

factors were significantly associated with health-promoting community activities: profession (social workers do the most, followed by nurses and then physicians); specific training in community activities; motivation; social sensitivity (collaboration with NGOs and participation in neighbourhood activities); perception of support and coverage by colleagues while doing community activities; years working in the health care team; and being a nursing-student tutor.

For peer review only

For peer review only - http://bmjopen.bmj.com/site/about/guidelines.xhtml

Table 4. Multivariate model of performing health-promoting community activities (CA)

Variable	General model (n=497) OR (95% CI)
**Profession**	
Nurse	Ref.
Doctor	0.44 (0.24-0.82)
Paediatrician	1.55 (0.69-3.46)
Social worker	25.26 (2.53-251.9)
**Specific training in CA**	1.91 (1.17-3.11)
**Collaboration with NGO or civic entity**	1.91 (1.17-3.13)
**Possibility of work covered by colleagues**	1.80 (1.08-2.99)
**Support from colleagues in doing CA**	2.92 (1.49-5.73)
**Motivated to perform CA**	
Disagree or strongly disagree	Ref.
Agree	2.29 (1.28-4.08)
Strongly disagree	3.74 (1.85-7.54)
**Participates in activities in the neighbourhood**	3.06 (1.86-5.05)
**Years at health centre**[†]	1.06 (1.03-1.09)
**Nursing-student tutor**	1.96 (1.09-3.53)
**Model Validity**	**Value**
**Chi^2**	221.338
**Nagelkerke R squared**	.483

Discussion

Our study of the participation of health care workers in health-promoting activities in the community suggests that factors of the individual health care professional were more

For peer review only - http://bmjopen.bmj.com/site/about/guidelines.xhtml

important than those of the health care team or the community. In particular, the specific profession, attitudes, and opinions of an individual seemed to be crucial. Professional workload, which was one of the more mentioned factors by the professionals in others studies, does not have any impact when adjusted by other individual factors. These results confirms the frAC Project[29] hypothesis and those of other authors[21,28]

Our assessment of variables related with health care team participation in health promoting community activities suggests that team characteristics had greater impact than community characteristics. One exception was the socio-economic level of the community. This makes sense in the context of the Spanish health care system, which attempts to offer equitable care to people according to their needs and community interventions are important tools to reduce inequalities in health care[35]. It should be noted that we found no relationship between the level of social interaction in the community and the implementation of community activities, in contrast with the findings of other authors[16-17].

Our results showing the important role of nurses in health promoting community activities are consistent with those of other authors[13-14,17,36] who highlighted the role of nurses in the development of community activities. This result is expected because nurses usually receive more training in community care. Another crucial group is social workers. Previous research reported the importance of social workers[13], but their role in the community varies in different regions of Spain. Their presence seems fundamental for development of health-promoting community activities in regions where they have significant roles in the community. In Spain, community work is generally not provided as part of primary health care; instead, it is relegated to professional volunteers or specific programs. Thus, our results recognize the importance of work by nurses and social workers and points to the possibility that their roles could be modified or clarified. We also found that health centres that train undergraduate nurses have a lower level of community activities but that professional nurse-

For peer review only - http://bmjopen.bmj.com/site/about/guidelines.xhtml

trainers have a greater involvement in health promoting community activities. This apparent contradiction may be resolved by considering two factors. First, the variable at the health centre level included training in various disciplines (from nursing to physiotherapy) without distinction, even though there is clearly difference among these in communitarian tradition. Second, the need for increased training would make it difficult for the health team to engage in community activities although, among the cases trainers carried out more activities than others. Health care workers who worked for more years at a centre were also more likely to perform community activities. This may be because medium- and long-term projects require continuity and stability, and it is more difficult for professionals who continually change centres or have temporary contracts to participate in health promoting community activities.

The pressures of working in health care and the heavy work burden were the main arguments offered by professionals when asked about their low participation in health promotion and prevention activities[19,22,24-27]. Our bivariate analysis supported this relationship, although the multivariate analysis did not. This implies that the professionals' perception was incorrect. In fact, the pressure of working in health care and the heavy work burden are not associated with low participation in health promoting community activities.

Our results indicated the importance of the opinions and attitudes of professionals when performing health promoting community activities, in accordance with other studies[15,21-22]. In fact, all variables regarding the opinions and attitudes of health care workers were significantly different between cases and controls. This suggests that individuals who perform community activities have similar attitudes: they employ a biopsychosocial[12,37] practice model, are more oriented towards health promotion, show recognition of the need for citizen participation in health services, and are more left-leaning in ideology. We also found that social sensitivity was an important variable in the model for professionals. This variable measures involvement of the professional with the community beyond consultation work. It is

For peer review only - http://bmjopen.bmj.com/site/about/guidelines.xhtml

not clear how some practices, responsibility for which is stipulated in primary care legislation and which should be similar for all professionals to assure fairness within the system, can be affected by a professional's personal discourse. Most health care professionals that we interviewed – both cases and controls -- said that their community roles were not well defined. This suggests that greater priority should be given to develop health promoting community activities in primary health care, and to clarify the role and responsibility of every professional about this question.

We found that having support within the health care team is associated with performing health promoting community activities. This finding should be highlighted, because all the professionals in this study were selected from the centres that engage in community activities so that, presumably, all cases can count on some support from their centres. It may be that some professionals were unaware of what their colleagues were doing, or that there were barriers to working with particular colleagues. Regardless of the explanation, this result suggests that application of the teamwork model[38], an assumed key feature of primary health care, has certain weaknesses.

Strengths and limitations

This study had an observational design, so we could not definitively establish causal relationships. Similarly, our definition of "health promoting community activities" may be a limitation because this concept can be defined or perceived in different ways; we made an attempt to control for this by use of a definition generated by expert consensus, which we then transformed into an algorithm. The questionnaires we used were *ad hoc* and some of the data regarding team variables, such as degree of social interaction in the community and the relationship between professionals, were provided by team managers. This may have led to a bias in information gathering due to a lack of knowledge or other unknown factors. Some variables that we did not examine could have explained participation in community activities,

For peer review only - http://bmjopen.bmj.com/site/about/guidelines.xhtml

such as support from primary care management, the health care administration and the centres' own coordinators or nursing managers. It would be valuable to assess the effect of these variables in future studies of the effect of team activities and priorities.

The main strengths of this study are that it examined a facet of health care that is relatively unexplored; the study subjects were from 5 different regions of Spain; the sample size was large; and it considered many distinct factors simultaneously. Furthermore, our design of the second study adjusted the effect of team and community variables matching cases and controls by teams, so a multilevel analysis was not necessary.

Implications for health care practice

Health care administrators who want to develop health-promotion activities for the community must provide visible and formal support for community initiatives, define better the expected communitarian tasks of each sanitarian professional, and provide job stability for all participating workers. Specific training is also important for the formation of health-promoting community activities. It may seem that attitudes, social sensitivity, or personal opinions are not modifiable; however, in our opinion, it is possible to modify these factors during undergraduate education. So, community perspective and their associated responsibility should be included in healthcare workers training

Conclusions

A key responsibility of primary health care is provision of health-promoting community activities, although not every team or professional makes them. We describe some factors related with this implication, as to work in a low socio-economic level community or to have specific training, support, motivation. social sensitivity or more years in the health team. There were also differences among professional categories, highlighting nurses and

For peer review only - http://bmjopen.bmj.com/site/about/guidelines.xhtml

social workers contributions. The pressures perceived by the professionals were not associated with involvement in community activities.

Acknowledgements

We want to express thanks to Magdalena Esteva for her patient reading of the manuscript.

Competing interests

All authors have completed the ICMJE uniform disclosure form at www.icmje.org/coi_disclosure.pdf and declare: no financial relationships with any organisations that might have an interest in the submitted work in the previous three years; no other relationships or activities that could appear to have influenced the submitted work

Funding

This study was supported by a grant from the Carlos III Health Institute (PI07/90383; PI07/90925; PI07/90636). It also received support from the Health Promotion and Preventive Activities in Primary Health Care Research Network (IAPP network RD 06/0018/), the ERDF funds, and the Institut de Salut de Palma (IDISPA). SM was contracted with the aid of a grant to stabilise employment in health research from the Carlos III Health Institute and benefited from a grant for further studies from the same institution. All institutions are public and did not have any role in study design, analysis, or publication.

Contributors

SM was the lead investigator, and was responsible for conception and design of the study, analysing data and writing the manuscript. **MR** has the original idea of the study. **SM, JR, JLRG, IMG, CBBA,** and **LES**, coordinated the field work in everyone of the regions involved. SM, JR, MJM, EZO, CBBA, LES, CV, MLLBA, TPJ, DMPM, MLLS, JLRG, RBV, CSV, MRB, MCV, MBC, ESF, MBD, IMG, BBR, AAL, SBC, ACT, BOB, RMB, PMP, MEO, MJPJ, MR contributed to the design and development of the study, have read the manuscript critically, made contributions and approved the final version.

For peer review only - http://bmjopen.bmj.com/site/about/guidelines.xhtml

Data Sharing Statement: Questionnaires and anonymised data are available on demand.

For peer review only

For peer review only - http://bmjopen.bmj.com/site/about/guidelines.xhtml

References

1. World Health Organisation. **The Ottawa Charter for Health Promotion**, Health Promotion. 1986 1 (4): i-v.

2. World Health Organization. **Health Promotion Glossary.** WHO 1998. [http://apps.who.int/iris/bitstream/10665/64546/1/WHO_HPR_HEP_98.1.pdf]

3. World Health Organisation: **Declaration of Alma Ata.** *WHO* 1978. [http://www.who.int/hpr/NPH/docs/declaration_almaata.pdf]

4. World Health Organisation: **Declarations of Jakarta** WHO 1997. [http://www.who.int/hpr/NPH/docs/jakarta_declaration_sp.pdf]

5. March S, Torres E, Ramos M. **Adult community health-promoting interventions in primary health care: A systematic review.** *Prev Med* 2015; 76 (2015) S94–S104

6. Ley General de Sanidad 14/1986, de 25 de abril. Boletín Oficial del Estado,nº102,(29/4/1986).

7. Real Decreto 1030/2006 del 15 de septiembre por el que se establece la cartera de servicios comunes del Sistema Nacional de Salud y el procedimiento para su actualización. *Boletín Oficial del Estado*,nº222,(16-9-2006).

8. Guldan GS: **Obstacles to community Health promotion**. *Soc sci med* 1996, **5**:689-695.

9. Irigoyen J: **La participación comunitaria en salud: Una reformulación necesaria.** In: *Nuevas perspectivas en Atención Primaria de Salud.* Edited by Aranda. Madrid: Díaz de Santos; 1994:183-221.

10. Gérvas J, Pérez-Fernández M, Palomo-Cobos L, Pastor-Sánchez R: **Veinte años de reforma de la Atención Primaria en España. Valoración para un aprendizaje por acierto/error.** Madrid: Ministerio Sanidad y Consumo 2005. [http://equipocesca.org/new/wp-content/uploads/2010/08/20-anos-de-reforma-de-la-ap-en-espana.pdf]

For peer review only - http://bmjopen.bmj.com/site/about/guidelines.xhtml

11. Regato P, Domínguez J, Sancho M: **Coordinación sanitaria y abordaje comunitario en atención primaria**. In: *La Atención Primaria de Salud en España y en sus comunidades autónomas.* Coordinated by Navarro V, Martín-Zurro A. Barcelona: Semfyc; 2009:149-168.

12. Turabián JL, Pérez B: **Grandes misterios. ¿Puedes ver al monstruo del lago Ness?: El modelo biopsicosocial y las actividades comunitarias.** *Aten Primaria* 2007, **39**:261-4.

13. March S, Soler M, Miller F, et al: **Variabilidad en la implantación de las actividades comunitarias de promoción de la salud en España.** *An Sist Sanit Navar.* In press.

14. Sáez S, Marqués F, Pallarés J, Serrano J, Orrio C, Martínez V: **Perfil de los profesionales de la salud de Lleida respecto a la educación para la salud (EpS).** *Aten Primaria* 1999, **24**:157-161.

15. Pérez-Franco B, Turabián JL, Magaña E, et al: **Factores asociados a la participación comunitaria en la atención primaria de la provincia de Toledo.** *Aten primaria* 1993, **12**: 339-344.

16. MJ Aguilar Idáñez: **La participación comunitaria en salud: ¿Mito o realidad?** Madrid: Díaz de Santos; 2001.

17. López E, Forcada C, Miller Frederick: **Factores que facilitan y dificultan el desarrollo de los proyectos comunitarios. Estudio observacional de la red AUPA de centros de atención primaria de Cataluña.** *Aten primaria* 2010, **42**:218-25.

18. Duro JC: **Actitudes de los profesionales de atención primaria de Madrid sobre los grupos.** *Aten Primaria* 2005, **36**:71-7.

19. Díaz G: **Factores relacionados con la práctica de educación sanitaria por los médicos de Atención primaria de Galicia.** *Rev Esp Salud Pública* 2001, **75**:529-540.

For peer review only - http://bmjopen.bmj.com/site/about/guidelines.xhtml

20. Maheux B Dufort, F Lambert J et al: **The professional attitudes and clinical practices of men and women generalists.** *Can Fam Physician* 1989, **35**:59-63.

21. Maheux B, Pineault R, Lambert J et al: **Factors influencing physicians' preventive practices.** *Am J Prev Med* 1989, **5**:201-206.

22. Myrand A, Beehler G, Kuo C, Mahoney M: **Explaining the de-prioritization of primary prevention: Physicians' perceptions of their role in the delivery of primary care.** *BMC Public Health* 2003, **3**:15. doi:10.1186/1471-2458-3-15.

23. Walter U, Flick U, Neuber A, et al: **Putting prevention into practice: qualitative study of factors that inhibit and promote preventive care by general practitioners, with a focus on elderly patients.** *BMC Family practice* 2010, **11**:68. doi:10.1186/1471-2296-11-68

24. Puig ribera A, McKenna J, Riddoch C: **Attitudes and practices of physicians and nurses regarding physical activity promotion in the Catalan primary health-care system.** *Europ J Public Health* 2005, **15**:569–75.

25. Wilhemsson S, Lindberg L: **Health promotion: Facilitators and barriers perceived by district nurses.** *Int J Nurs Practice* 2009, **15**:156-163.

26. Rubio-Valera M, Pons-Vigues M, Martinez-Andres M et al. **Barriers and Facilitators for the implementation of primary prevention and health promotion activities in primary care: A Synthethesis through meta-etnography.** Plos one 2014;9:e89554. Doi: 10.1371/journal.pone.0089554.

27. Badertscher N, Rossi PO, Rieder A et al: **Attitudes, barriers and facilitators for health promotion in the elderly in primary care.** *Swiss Med Wkly* 2012;**142**:w13606.

28. Maheux B, Pineault L, Béland F et al: **Factors influencing physicians' orientation toward prevention.** *Am J Prev Med.* 1987, **3**:12-8.

For peer review only

For peer review only - http://bmjopen.bmj.com/site/about/guidelines.xhtml

29. March S, Ripoll J,Ruiz-Giménez JL, et al: **Observational study on factors related to health-promoting community activity development in primary care (frAC Project): a study protocol.** *BMJ Open* 2012, **2**. e001287 doi:10.1136/bmjopen-2012-001287.

30. March S, Jordán M, Montaner I et al. **¿Qué hacemos en el barrio? Descripción de las actividades comunitarias de promoción de la salud en atención primaria: Proyecto frAC.** Gac Sanit 2014; **28**:267-73.

31. March S, Ramos M, Soler M, Ruiz-JiménezJL, Miller F, Domínguez J, et al: **Revisión documental de experiencias de actividad comunitaria en atención primaria de salud.** *Aten Primaria* 2011, **43**:289-296.

32. T Martínez-cañavate: **Modelo multinivel explicativo de la utilización de las consultas de atención primaria en Andalucía**. *Phd tesis.* Granada university, faculty of medicine; 2007. [http://hera.ugr.es/tesisugr/16905234.pdf]

33. Mira JJ, Llinás G, Gil V, Orozco D, Palazón I, Vitaller J: **Validación de un instrumento para identificar estilos de práctica profesional del médico de atención primaria.** *Aten Primaria* 1998, **21**:14-22.

34. Rey del Castillo P: **Nota metodológica sobre los indicadores del barómetro.** *Rev Española de Investigaciones Sociológicas* 2004, **108**:151-78.

35. Comisión para reducir las desigualdades en salud en España: **Propuesta de políticas e intervenciones para reducir las desigualdades en salud en Espanya**. *Gac Sanit* 2012. doi:10.1016/j.gaceta.2011.07.024.

36. Hogg W, Dahrouge S. Russell G et al: **Health promotion activity in primary care: performance of models and associated factors.** *Open medicine* 2008, **3**:165-73.

37. Engel GL: **The need for a new medical model: a challenge to biomedicine.** Science 1977, **196**:129–36.

For peer review only

For peer review only - http://bmjopen.bmj.com/site/about/guidelines.xhtml

38. Grumbach K, Bodenheimer T: **Can Health Care teams improve Primary Care practice?** JAMA 2004, **291**:1246-51.

For peer review only

For peer review only - http://bmjopen.bmj.com/site/about/guidelines.xhtml

STROBE Statement—Checklist of items that should be included in reports of ***case-control studies***

	Item No	Recommendation
**Title and abstract**	1	(*a*) Indicate the study's design with a commonly used term in the title or the abstract<br>OK. p1.
		(*b*) Provide in the abstract an informative and balanced summary of what was done and what was found<br>OK. p2
**Introduction**		
Background/rationale	2	Explain the scientific background and rationale for the investigation being reported<br>OK. p4-5
Objectives	3	State specific objectives, including any prespecified hypotheses<br>OK. p5
**Methods**		
Study design	4	Present key elements of study design early in the paper<br>OK. p5
Setting	5	Describe the setting, locations, and relevant dates, including periods of recruitment, exposure, follow-up, and data collection<br>OK. p6
Participants	6	(*a*) Give the eligibility criteria, and the sources and methods of case ascertainment and control selection. Give the rationale for the choice of cases and controls<br>OK. p6
		(*b*) For matched studies, give matching criteria and the number of controls per case<br>OK. p6.
Variables	7	Clearly define all outcomes, exposures, predictors, potential confounders, and effect modifiers. Give diagnostic criteria, if applicable<br>OK. p6-8.
Data sources/ measurement	8*	For each variable of interest, give sources of data and details of methods of assessment (measurement). Describe comparability of assessment methods if there is more than one group<br>OK. p6-8
Bias	9	Describe any efforts to address potential sources of bias<br>OK. p17-18
Study size	10	Explain how the study size was arrived at<br>OK. p8
Quantitative variables	11	Explain how quantitative variables were handled in the analyses. If applicable, describe which groupings were chosen and why<br>OK. p6-8
Statistical methods	12	(*a*) Describe all statistical methods, including those used to control for confounding<br>OK. p8-9
		(*b*) Describe any methods used to examine subgroups and interactions<br>OK. p8-9
		(*c*) Explain how missing data were addressed<br>OK. p8-9
		(*d*) If applicable, explain how matching of cases and controls was addressed<br>Not applicable
		(*e*) Describe any sensitivity analyses

For peer review only

For peer review only - http://bmjopen.bmj.com/site/about/guidelines.xhtml

		Not applicable
**Results**		
Participants	13*	(a) Report numbers of individuals at each stage of study—eg numbers potentially eligible, examined for eligibility, confirmed eligible, included in the study, completing follow-up, and analysed<br>OK. p11
		(b) Give reasons for non-participation at each stage<br>OK. p11
		(c) Consider use of a flow diagram<br>OK. It is not necessary.
Descriptive data	14*	(a) Give characteristics of study participants (eg demographic, clinical, social) and information on exposures and potential confounders<br>OK. Tables 1 and 2.
		(b) Indicate number of participants with missing data for each variable of interest<br>OK. Tables 1-3.
Outcome data	15*	Report numbers in each exposure category, or summary measures of exposure<br>OK. Tables 1-3.
Main results	16	(*a*) Give unadjusted estimates and, if applicable, confounder-adjusted estimates and their precision (eg, 95% confidence interval). Make clear which confounders were adjusted for and why they were included<br>OK. Tables 1-3.
		(*b*) Report category boundaries when continuous variables were categorized<br>OK. Tables 1-3.
		(*c*) If relevant, consider translating estimates of relative risk into absolute risk for a meaningful time period<br>OK. It is not necessary.

For peer review only

Other analyses	17	Report other analyses done—eg analyses of subgroups and interactions, and sensitivity analyses<br>OK. p8-9.
**Discussion**		
Key results	18	Summarise key results with reference to study objectives<br>OK. p14-15.
Limitations	19	Discuss limitations of the study, taking into account sources of potential bias or imprecision. Discuss both direction and magnitude of any potential bias<br>OK. p17-18.
Interpretation	20	Give a cautious overall interpretation of results considering objectives, limitations, multiplicity of analyses, results from similar studies, and other relevant evidence<br>OK. p15-18.
Generalisability	21	Discuss the generalisability (external validity) of the study results<br>OK. p17-18
**Other information**		
Funding	22	Give the source of funding and the role of the funders for the present study and, if applicable, for the original study on which the present article is based<br>OK. p19

*Give information separately for cases and controls.

Note: An Explanation and Elaboration article discusses each checklist item and gives methodological background and published examples of transparent reporting. The STROBE checklist is best used in conjunction with this article (freely available on the Web sites of PLoS Medicine at http://www.plosmedicine.org/, Annals of Internal Medicine at http://www.annals.org/, and Epidemiology at http://www.epidem.com/). Information on the STROBE Initiative is available at http://www.strobe-statement.org.

For peer review only

For peer review only - http://bmjopen.bmj.com/site/about/guidelines.xhtml

BMJ Open

Factors related to the development of health-promoting community activities in Spanish primary health care: Two case-control studies

Journal:	*BMJ Open*
Manuscript ID	bmjopen-2017-015934.R4
Article Type:	Research
Date Submitted by the Author:	17-Jul-2017
Complete List of Authors:	March, Sebastià; Baleares Health services-IbSalut, Primary Care Research Unit of Mallorca; Instituto de investigación en salud de Palma (IDISPA)<br>Ripoll, Joana; Baleares Health services-IbSalut, Primary Care Research Unit of Mallorca; Baleares Health services-IbSalut, Primary Care Research Unit of Mallorca<br>Jordan Martin, Matilde Jordan Martin; Estrecho de Correa Primary health center, Madrid Health Services<br>Zabaleta del Olmo, Nieves; Fundacio Jordi Gol i Gurina, IDIAP<br>Benedé Azagra, Carmen Belén; Aragon Health Services, Oliver Primary health center<br>Elizalde Soto, Lázaro; Gobierno de Navarra Departamento de Salud, public health institute; Public Health and epidemiology CIBER,<br>Vidal, Mª Clara; Baleares Health services-IbSalut, Primary Care Research Unit of Mallorca; Instituto de investigación en salud de Palma (IDISPA)<br>Bauzà Amengual, M. de Lluc; Balearic Islands University,<br>Planas Juan, Trinidad; Baleares Health services-IbSalut, Son Gotleu Primary health care center<br>Maria Pèrez, Damiana Maria Pèrez; Son Gotleu Primary health center, Baleares Health services-IbSalut<br>Llull Sarralde, Micaela; Baleares Health services-IbSalut, San Agustí Primary health care center<br>Ruiz-Giménez, Juan Luís; Madrid Health Services, Madrid, Vicente Soldevilla Primary health care center<br>Bajo Viñas, Rosa; Madrid Health care services, Loeches Primary health care center<br>Solano Villarubia, Carmen Solano Villarubia; madrid Health care services, DA Suroeste Health center<br>Rodriguez Bajo, Maria<br>Cordoba Victoria, Manuela Cordoba Victoria; SOMAMFyC<br>Badia Capdevila, Marta; Institut Catala De La Salut, El carmel Health center<br>Serrano Ferrandez, Elena; Institut Catala De La Salut, El carmel health center<br>Bosom Diumenjo, Maria; Institut Catala De La Salut, Sant Rafel Primary Health Center<br>Montaner-Gomis, Isabel; Institut Catala De La Salut, El Carmel Health center<br>Bolibar-Ribas, Buenaventura; Fundacio Jordi Gol i Gurina, IDIAP<br>Antoñanzas Lombarte, Angel; Aragon Health services, Delicias del Sur health center

	Bregel Cotaina, Samantha; Instituto Aragones de Ciencias de la Salud, Primary health care research unit<br>Calvo Tocado, Ana; Zaragoza Public Health provincial subdirection<br>Olivan Blázquez, Barbara; Instituto Aragones de Ciencias de la Salud, primary health care research unit<br>Magallon Botaya, Rosa; Aragon Health services, Arrabal health center<br>Marín Palacios, Pilar Marín Palacios; Gobierno de Navarra Departamento de Salud, Public health institute<br>Echauri Ozcoidi, Margarita; Gobierno de Navarra Departamento de Salud, public health institute<br>Perez-Jarauta, Mª Jose Perez-Jarauta; Gobierno de Navarra Departamento de Salud, public health institute<br>Llobera, Joan; Baleares Health services-IbSalut, 1. Primary Care Research Unit of Mallorca; Instituto de investigación en salud de Palma (IDISPA)<br>Ramos, Maria; Govern de les Illes Balears Conselleria de Salut Familia i Benestar Social, Direcció General de salut Pública i Participació; Instituto de investigación en salud de Palma (IDISPA)
**Primary Subject Heading**:	General practice / Family practice
Secondary Subject Heading:	Health services research, Public health
Keywords:	Health promotion, Primary health care, Health services research, Community health, Health team, Health professionals

view only

Factors related to the development of health-promoting community activities in Spanish primary health care: Two case-control studies

Sebastià March[1], Joana Ripoll[1], Matilde Jordan Martin[2], Edurne Zabaleta-del-Olmo[3][3], Carmen Belén Benedé Azagra[4], Lázaro Elizalde Soto[5], Mª Clara Vidal[1], María de Lluc Bauzà Amengual[6], Trinidad Planas Juan[7], Damiana Maria Pérez Mariano[7], Micaela Llull Sarralde[8], Juan Luís Ruiz-Giménez[9], Rosa Bajo Viñas[10] , Carmen Solano Villarubia[11], Maria Rodriguez Bajo[12], Manuela Cordoba Victoria[13], Marta Badia Capdevila[3], Elena Serrano Ferrandez[3], Maria Bosom Diumenjo[14], Isabel Montaner Gomis[15], Bonaventura Bolíbar-Ribas [3], Angel Antoñanzas Lombarte[16], Samantha Bregel Cotaina[17], Ana Calvo Tocado[18], Barbara Olivan Blázquez[17], Rosa Magallón Botaya[19], Pilar Marín Palacios[5], Margarita Echauri Ozcoidi[5], María Jose Perez-Jarauta[5], Joan Llobera[1], Maria Ramos[20].

1. Primary Care Research Unit of Mallorca, Baleares Health Services-IbSalut, Palma de Mallorca, Spain.
2. Estrecho de Correa Primary Health Center, Madrid Health Services, Madrid, Spain.
3. Institut Universitari d'Investigació en Atenció Primària Jordi Gol (IDIAP Jordi Gol), Barcelona, Spain..
4. Oliver Primary Health Center, Aragon Health Services, Zaragoza, Spain.
5. Navarra Public Health Institute, and Public Health and Epidemiology CIBER, Pamplona, Spain.
6. Balearic Islands University, Palma de Mallorca, Spain.
7. Son Gotleu Primary Health Center, Baleares Health Services-IbSalut, Palma de Mallorca, Spain.
8. San Agustí Primary Health Center, Baleares Health Services-IbSalut, Palma de Mallorca, Spain.
9. Vicente Soldevilla Primary Health Center, Madrid Health Services, Madrid, Spain.
10. Loeches Primary Health Center, Madrid Health Services, Madrid, Spain.
11. DA Sureste Primary Health Center, Madrid Health Services, Madrid, Spain.
12. Freelance, Madrid, Spain
13. SOMAMFYC, Madrid, Spain
14. Sant Rafael Primary Health Center, Àmbit d'Atenció Primària Barcelona-Ciutat, Institut Català de la Salut, Barcelona, Spain.
15. El Carmel Primary Health Center, Àmbit d'Atenció Primària Barcelona-Ciutat, Institut Català de la Salut, Barcelona, Spain.
16. Delicias del Sur Primary Health Center, Aragon Health Services, Zaragoza, Spain.
17. Primary Care Research Unit, Aragon, Spain
18. Zaragoza Public Health department, Zaragoza, Spain.
19. Arrabal Primary Health Center, Aragon Health Services, Zaragoza, Spain.
20. Public Health Department, Balearic Islands Health Department, Spain.

Corresponding author:
Sebastià March
E-mail: smarch@ibsalut.caib.es,

For peer review only - http://bmjopen.bmj.com/site/about/guidelines.xhtml

Abstract

Objective: Spanish primary health care teams have the responsibility of performing health-promoting community activities, although such activities are not widespread. Our aim was to identify the factors related to participation in those activities.

Design: Two case-control studies. **Setting:** Performed in primary care of 5 Spanish regions. **Subjects:** In the first study, cases were teams that performed health promoting community activities and controls were those that did not. In the second study (on case teams from the first study), cases were professionals who developed these activities and controls were those who did not. **Main outcome measures:** Team, professional, and community characteristics collected through questionnaires (team managers/professionals) and from secondary sources.

Results: The first study examined 203 teams (103 cases, 100 controls). Adjusted factors associated with performing community activities were percentage of nurses (OR=1.07, 95%CI =1.01-1.14), community socio-economic status (higher *vs.* lower OR =2.16, 95%CI=1.18-3.95), and performing undergraduate training (OR=0.44, 95%CI=0.21-0.93). In the second study, 597 professionals responded (254 cases, 343 controls). Adjusted factors were professional classification (physicians do fewer activities than nurses and social workers do more), training in community activities (OR=1.9, 95%CI =1.2-3.1), team support (OR=2.9, 95%CI =1.5-5.7), seniority (OR=1.06, 95%CI=1.03-1.09), nursing tutor (OR =2.0, 95%CI =1.1-3.5), motivation (OR =3.7, 95%CI=1.8-7.5), collaboration with non-governmental organizations (OR=1.9, 95%CI=1.2-3.1), and participation in neighbourhood activities (OR=3.1, 95%CI=1.9-5.1).

Conclusions: Professional personal characteristics, such as social sensitivity, profession, to feel team support, or motivation, have influence in performing health-promoting community

For peer review only - http://bmjopen.bmj.com/site/about/guidelines.xhtml

activities. In contrast to the opinion expressed by many professionals, workload is not related to performance of health-promoting community activities.

Keywords: Health promotion, Primary health care, Health services research, Community health, Health team, health professionals

Strengths and limitations of this study

- This study examined a topic relatively unexplored, community health promotion by primary health care teams.
- A case-control approach was chosen, where cases were subjects (professionals or teams) who were developing health promoting community activities, and controls those who did not.
- Design allows exploration of many distinct factors simultaneously, from different sources, and in two levels: Health team and professional.
- An operative definition of community activities was used, which recognizes the heterogeneity of health promotion interventions developed with the community in Spanish primary health care settings.
- Because of its observational design, there are limitations to establishing causal inferences.

For peer review only

Introduction

The Ottawa Charter for Health Promotion[1] defined Health promotion as the process of enabling people to increase control over, and to improve, their health. This definition implies the importance of empowering people[2] to take control over what determines their health. One tool to work in this direction are health promoting community activities. These can come in a variety of forms, from walking groups in a neighbourhood to holding meetings with local authorities, schools or civil associations to develop specific health programs. Experts at numerous international conferences have agreed on the importance of health-promoting community activities for improving public health[1,3-4], and there is some evidence that these activities are effective[5].

Since the implementation of health care reform in Spain during the 1980's[6-7], primary health care teams have had responsibility for carrying out health promotion activities. These teams, located in health care centres throughout the country, are composed of physicians, community nurses, paediatricians, midwives, social workers, and other health care professionals (gynaecologists, psychologists, and psychiatrists) in a system that offers almost-universal and free health care. These conditions are ideal for an interdisciplinary approach to encourage health promoting community activities. However, as in other countries[8], implementation of these interventions differs greatly among the different health care teams[9-11], and relatively few professionals are involved[11-13].

There has been limited studies of the factors related to the development of health-promoting interventions in the community. These factors may be broadly classified as those related to the community, to the team, or to the individual professionals. With regard to community factors, some studies indicated that the rural/urban environment[14], size of the municipality[15], level of social interaction within the community[15-17], and presence of a focal point for participation in community health initiatives[15-16] influence the practice of health

For peer review only - http://bmjopen.bmj.com/site/about/guidelines.xhtml

promoting community activities. The primary health care team factors include job satisfaction[18], management of time and setting within the center[14], composition and organization of the team[15-16], years of team operation[15], intra-team support[17], and work burden[8,19]. Studies of professionals have highlighted sex[19-21], age[15,20], professional status[14], specific training[22-25], motivation[17-18], and the model applied (biomedical or psychosocial)[18]. The attitudes and beliefs of the professionals, such as trust in the effectiveness of preventive actions[18,22,25-27], self-reported efficacy in carrying out activities[8,22-23], or to support community participation[15,21] are also important. An additional element is professionals' social sensitivity, as evaluated through their personal political leanings[28]. According to professionals themselves, the most important factor is the lack of time available to address the care burden[19,22,24-27]. All these features have been observed separately, mostly in descriptive studies.

This study presents some of the results of the Factors Related to Health-Promoting Community Activity Development in Primary Care (frAC) Project, whose complete methodology and aims were described elsewhere[29]. The purpose of this study is to identify primary health care team and community factors related to implementation of health promoting community activities in primary care and factors that may explain why only some professionals within a team participate in those community activities.

Methods

Two case-control studies were performed. In the first, the cases were the teams that performed health promoting community activities and the controls were those that did not. The second study examined only teams that implemented these community activities to adjust for community/team factors; in this second analysis the cases were professionals who developed these activities and the controls were those who did not. The study was set in

For peer review only - http://bmjopen.bmj.com/site/about/guidelines.xhtml
For peer review only

primary health care in five regions of Spain (Balearic Islands, Catalonia, Aragon, Madrid, and Navarra). Data collection was carried out between 2009-2010.

Selection of cases and controls

We used a conceptual definition of health promoting community activity reached in a previous study [30-31]. This was converted into an algorithm for use in this study[29]: this activity had to be a non-isolated activity carried out in the previous year, in which the professional participated on behalf of the health centre. Furthermore, it had to have involvement and active participation of the community, or the population had to have the capacity to influence the intervention, or it was a cross-sector activity involving collaboration with entities outside of health care (*e.g.* education, social services). Health preventive interventions which does not have a health promotion approach were excluded.

We telephoned all the health centres in the research areas asking if they had participated in health promoting community activities during the last year. If they did, we contacted a participant of the activity and gave him/her a questionnaire to confirm that the activity met our inclusion criteria. For the first study, health teams that participated in at least one community activity in the last year were selected as cases, and controls were those who had not participated. For the second study, we selected all professionals who participated in the implementation of those community activities as cases and professionals who did not participate as controls. In every health team we selected at least one control for every case.

Variables

Information was gathered through three distinct questionnaires (available on request) that were given to the community, the team, and the professionals[29]. Description of health promoting community activities are described elsewhere[30].

The data collected from the community (from questionnaires to centre managers and secondary sources) were: demographic composition; socio-economic level; degree of social

For peer review only - http://bmjopen.bmj.com/site/about/guidelines.xhtml

interaction; health centre setting; geographical dispersion based on an ordinal variable used by the National Health System for resource allocation (from G1 [less dispersed] to G4 [more dispersed]); and existence of health boards (community participation agencies described in health legislation)[16].

The data collected from the team (collected from secondary sources and questionnaires to centre managers) were: percentages of different professionals (nurses, physicians, and paediatricians); year of opening; population of service area; professional/population and doctor/nurse ratios; availability of a space in the centre to carry out group activities and presence of a team member responsible for health education; collaboration with the area health board where applicable; training of resident physicians; nurses and nursing or physiotherapy students; evaluation of professional relationships between distinct professional disciplines (based on a Likert-type scale ranging from 1 [poor] to 5 [excellent]).

The data collected from the professionals (from individual self-administrated questionnaires) were: sex; age; profession; working situation; health care burden; size of quota allocated; average number and duration of consultations per day at the centre or at home; tutoring; participation in research or specific training in health promotion in the previous five years; years since graduation; years worked in primary care and as a member of the current health care team; autonomy to organize scheduling; arrangements with colleagues to cover health care tasks while carrying out community activities; support of fellow team members in conducting these activities; working atmosphere; job satisfaction; and self-assessed efficacy in conducting community work (based on a Likert-type scale ranging from 1 [poor] to 5 [excellent]).

Opinions and attitudes were measured by responses to various statements developed by the research team, using a Likert-type scale with four options ranging from "strongly

For peer review only - http://bmjopen.bmj.com/site/about/guidelines.xhtml

For peer review only

agree" to "strongly disagree". The statements dealt with trust in the effectiveness of the health promotion community activities, motivation to carry out these activities, citizen participation in health care decision-making, need to strengthen agencies involved in citizen participation in health care, responsibility for primary care in the community, definition of professional role, link between health education and community participation, and the effectiveness of the boards in driving community activities. A four-item validated scale was used to evaluate each professional's clinical practice style (biomedical-psychosocial)[32-33] by assessing the attitude towards the demands of psychosocial and clinical care at work. Finally, social sensitivity data were collected through proxy variables: participation during the previous twelve months in activities in the neighbourhood where the professional works; involvement in any non-governmental organization or civil association; and political tendency (assessed through a visual scale adapted from the Centre for Sociological Research[34] Spanish monthly barometer).

Calculation of sample size

For the first study, we calculated that at least 91 teams would be needed in each group based on an OR of 2.5, an alpha risk of 0.05, a beta of 0.2, and an expected proportion of 0.5 in the controls. For the second study, we calculated that at least 222 professionals would be needed in each group based on an OR of 1.75, an alpha risk of 0.05, a beta of 0.2, and an expected proportion of 0.5 in the controls.

Data analysis and management

A descriptive analysis of all variables was carried out to compare their distributions in each group. Categorical variables are expressed as percentages and continuous variables as means with 95% confidence intervals (95% CIs) or as medians and percentiles depending on the distribution. Main variable was, for both studies, to be case or control. The relationship with the main variable was assessed using the chi-squared test for categorical variables and Student's *t*-test and the Mann-Whitney U non-parametric test for continuous

For peer review only - http://bmjopen.bmj.com/site/about/guidelines.xhtml

variables. The strength of an association was expressed through unadjusted OR and its 95% CI.

We also performed a logistic regression analysis to calculate adjusted ORs. We constructed a first model using each variable whose *p*-value was less than 0.2 in the bivariate analysis. From this model, we tested and compared variations using the maximum likelihood method, eliminating variables that had *p*-values less than 0.05 in the Wald test. The collinearity of the variables was examined and interactions tested. We established a different model for the health care teams and for the professionals. All analyses were conducted using SPSS ver. 14 and Epidat ver. 3.1 software.

Ethical aspects

The study was approved by the Research Commissions in all the participant regions and by the Research Ethics Committee in Madrid (Area 1) and the University Research Institute in Primary Care (IDIAP) Jordi Gol in Barcelona. The questionnaire was completed anonymously by the professionals, with the identity of individuals and centres coded to ensure blinding of the researchers.

Results

Factors related to the health care team

We examined 203 health care teams (103 cases [50.7%] and 100 controls) in the first study. Table 1 shows the results of the initial bivariate analysis. The variables significantly related to team involvement in community activities were the socioeconomic level of the community, collaboration on training in physiotherapy or nursing, participation in a health board, percentage of nurses in the team, patient/physician ratio, and patient/nurse ratio.

For peer review only - http://bmjopen.bmj.com/site/about/guidelines.xhtml

Table 1. Relationship of community and primary health care team (PHT) characteristics with performance of health-promoting activities in the community.

Nominal Variables	PHT cases N (%)	PHT controls N (%)	Unadjusted OR (95% CI)
**Health Care setting**			
Urban	73 (71.6)	68 (68.0)	Ref.
Rural	29 (28.4)	32 (32.0)	0.84 (0.46-1.54)
**Socioeconomic level**			
High and medium-high	52 (51)	65 (65)	1
Medium-low and low	50 (49)	35 (35)	**1.79 (1.01-3.14)**
**Degree of social interaction**			
High	22 (23.9)	23 (26.1)	Ref.
Medium	43 (46.7)	35 (39.8)	1.28 (0.61-2.68)
Low	27 (29.3)	30 (34.1)	0.94 (0.43-2.06)
**Population of municipality**			
>1 million	47 (45.6)	48(48)	Ref.
500,000-1 million	7 (12.6)	7 (7)	1.90 (0.70-5.18)
10,000-500,000	22 (21.4)	33 (33)	0.68 (0.35-1.33)
<10,000	21 (20.4)	12 (12)	1.79 (0.79-4.04)
**Degree of geographical dispersion**			
G1 (less dispersed)	62 (66.7)	60 (63.8)	Ref.
G2	11 (11.8)	17 (18.1)	0.63 (0.27-1.45)
G3	9 (9.7)	11 (11.7)	0.79 (0.31-2.05)
G4 (more dispersed)	11 (11.8)	6(6.4)	1.77 (0.67-5.10)
**Active health board**	67 (66.3)	60 (61.9)	1.21 (0.68-2.17)
**Training centre**			
Resident Doctors	35 (34.7)	34 (34.3)	1.01 (0.56-1.82)
Resident Nurses	23 (23.5)	29 (30.2)	0.71 (0.37-1.34)
Nursing/physiotherapy students	73 (72.3)	84 (85.7)	**0.43 (0.21-0.89)**
**Space for group activities**	83 (81.4)	79 (79)	1.16 (0.58-2.32)
**Health education manager**	45 (44.1)	46 (46)	0.93 (0.53-1.61)
**Participation in health board**	56 (83.6)	38 (63.3)	**2.95 (1.28-6.78)**
**Continuous variables**	**Mean (95% CI)**		**p***
PHT operational years	14.3 (13-15.2)	12.9 (11.7-14.1)	.075
Total No. professionals	36.4 (33.1-39.6)	37.5 (34-41.1)	.486
% nurses	35.4 (34.5-36.4)	33.5 (32.4-34.5)	**.010**
% paediatricians	7.0 (6.3-7.7)	7.4 (6.7-8.1)	.450
Population covered by PHT	20031 (18059-22002)	21770.4 (19530-24010)	.247
	**Median (IQR)**		**p****
% doctors	33.3 (29.5-38.3)	33.3 (29.9-36.4)	.532
% non-EU immigrants	11.1 (5.8-16.2)	11.1 (8.6-15.7)	.690
% population <15 years	13.0 (11.6-14.7)	12.9 (11.3-14.4)	.352
% population >65 years	17.8 (15.0-21.8)	18.9 (13.8-21.5)	.976
% population women	51 (49.5-53)	51.2 (50.3-53.1)	.403
Population ratio registered/professional	524 (444-658)	578 (482-669)	.122
Population ratio registered/doctor	1659 (1454-1905)	1734 (1533-2040)	**.034**
Population ratio registered/nurse	1508 (1284-1847)	1723 (1434-2057)	**.008**
Doctor/nurse ratio	.94 (.86-1.09)	1 (.89-1.14)	.085
**Professional relationships**			
Doctor::nurse	4 (3-4)	4 (3-4)	.376
Doctor::social worker	4 (3-5)	4 (3-4)	.978
Nurse::social worker	4 (3-5)	4 (3-4)	.845

Data from Spain 2009. For dichotomous variables, the reference value is always absence of the variable.* Student's t-test P. ** Mann-Whitney U test P. Bold-face: $p \leq 0.05$.

For peer review only

For peer review only - http://bmjopen.bmj.com/site/about/guidelines.xhtml

Eleven teams (5.0%) were excluded from the logistic regression analysis due to missing data. The 3 variables remaining in the final model were percentage of nurses in the team (OR = 1.07 for every 1% increase; 95% CI = 1.01-1.14); socio-economic level of the community (high and medium-high *vs.* low and medium-low OR = 2.16; 95% CI = 1.18-3.95); and having undergraduate training at the health care centre (OR = 0.44; 95% CI = 0.21-0.93). Nagelkerke's R squared was 0.11.

Factors related to professionals

A total of 597 professionals responded to the questionnaire, 254 (42.4%) cases and 343 (57.5%) controls. Refused to participate 96 professionals (62 cases and 34 controls). Participants were 298 nurses (52.4%), 191 doctors (33.6%), 58 paediatricians (10.2%), and 22 social workers (3.8%). Tables 2 and 3 show the results of the bivariate descriptive analysis. Table 2 shows that the variables related to involvement in health promoting community activities were sex, profession, training, self-confidence, and work situation. Also years of experience, age, workload, practice style and political orientation seems to be related. Table 3 summarizes the responses of the professionals' opinions and attitudes to health promotion, community participation, and social sensitivity.

For peer review only

For peer review only - http://bmjopen.bmj.com/site/about/guidelines.xhtml

Table 2. Relationship of professional variables with performance of health-promoting community activities (CA).

Nominal Variables	Cases N (%)	Controls N (%)	Unadjusted OR (95% CI)*
**Sex**			
Male	41 (16.4)	84 (25.3)	Ref.
Female	209 (83.6)	248 (74.7)	**1.72 (1.13-2.61)**
**Profession**			
Nurse	156 (64.7)	142 (43.3)	Ref.
Doctor	40 (16.6)	151 (46)	**0.24 (0.16-0.36)**
Paediatrician	24 (10)	34 (10.4)	0.64 (0.36-1.3)
Social worker	21 (8.7)	1 (0.3)	**19.11 (2.53-143.9)**
**Specialized General practitioner**	22 (55)	91 (60.3)	0.81 (0.39-1.62)
**Tutor of Resident doctors**	10 (25)	37 (24.5)	1.02 (0.45-2.3)
**Nursing tutors**	93 (59.6)	58 (40.8)	**2.13 (1.34-3.39)**
**Work situation**			
Permanent	204 (80.3)	238 (70)	Ref.
Temporary contracts	40 (15.7)	74 (21.8)	**0.63 (0.41-0.96)**
Other	10 (3.9)	28 (8.2)	**0.41 (0.19-0.87)**
**Participation in research projects in last 5 years**	115 (47.7)	147 (43.9)	1.16 (0.83-1.62)
**Specific training in CA**	142 (58.2)	104 (31.7)	**2.99 (2.12-4.23)**
**Job satisfaction**			
None/little	18 (7.1)	34 (10)	Ref.
Average	64 (25.3)	129 (38.1)	0.93 (0.49-1.78)
Quite a lot/a lot	171 (67.5)	176 (51.9)	1.83 (0.99-3.37)
**Working environment satisfaction**			
None/little	14 (5.6)	33 (9.8)	Ref.
Average	82 (32.5)	103 (30.5)	1.87 (0.94-3.73)
Quite a lot/a lot	156 (61.9)	202 (59.8)	1.82 (0.94-3.51)
**Self-confidence to carry out CA**			
None/little	31 (12.3)	72 (21.6)	Ref.
Average	81 (32.1)	154 (46.2)	1.22 (0.74-2.01)
Quite a lot/a lot	140 (55.5)	107 (32.1)	**3.03 (1.86-4.96)**
**Continuous Variables**	**Median (p25-p75)**		**p***
**Age**	49 (42-54)	48 (39-53)	**.048**
**Years qualified**	25 (18-31)	24 (15-29)	**.024**
**Years in primary care**	19 (11-23)	17 (10-23)	.552
**Years in current health centre**	11 (4-18)	7 (2-15)	**.000**
**Size of assigned quota**	1737 (1300-2043)	1600 (1300-1900)	**.026**
**Average daily consultations**	20 (15-30)	30 (20-35)	**.000**
**Average daily home visits**	1 (0.5-3)	1 (1-2)	.932
**Average consultation time**	10 (10-15)	10 (7-12)	**.000**
**Average home-visit time**	25 (20-30)	20 (15-30)	**.026**
**Biomedical practice style (+) Psychosocial (-)**	8 (6-9)	8 (7-9)	**.040**
**Political orientation: Left (-) right (+).**	2 (1-5)	3 (2-5)	**.026**

Data from Spain 2009. For dichotomous variables, the reference value is always absence of the variable. Bold-face: Chi-squared *p* ≤less than 0.05. * Mann-Whitney U test.

For peer review only - http://bmjopen.bmj.com/site/about/guidelines.xhtml

Table 3. Professional attitudes in cases and controls regarding health-promoting community activities (CA).

Variable	Cases N (%)	Controls N (%)	Unadjusted OR (IC 95%)
**Confidence in CA efficacy**			
Disagree/strongly disagree	9 (3.6)	19 (5.7)	Ref.
Agree	110 (43.8)	196 (59.0)	1.18 (0.51-2.71)
Strongly agree	132 (52.6)	117 (35.2)	**2.38 (1.03-5.46)**
**Motivated to carry out CA**			
Disagree- strongly disagree	44 (17.5)	149 (45.4)	Ref.
Agree	113 (45.0)	131 (39.9)	**2.92 (1.91-4.44)**
Strongly agree	94 (37.5)	48 (14.6)	**6.63 (4.08-10.7)**
**Citizen participation in health**			
Disagree/strongly disagree	23 (9.2)	46 (14.0)	Ref.
Agree	122 (49.0)	191 (58.1)	1.27 (0.73-2.21)
Strongly agree	104 (41.8)	92 (28.0)	**2.26 (1.27-4.01)**
**Citizen participation in health services**			
Disagree/strongly disagree	24 (9.6)	48 (14.5)	Ref.
Agree	109 (43.6)	198 (59.8)	1.10 (0.64-1.89)
Strongly agree	117 (46.8)	85 (25.7)	**2.75 (1.56-4.83)**
**Professionals' role in the community is well-defined**			
Disagree/strongly disagree	126 (51)	223 (66.8)	Ref.
Agree	103 (41.7)	97 (29)	**1.87 (1.32-2.67)**
Strongly agree	18 (7.3)	14 (4.2)	**2.27 (1.09-4.73)**
**Health education and community participation are closely linked**			
Disagree/strongly disagree	20 (8.1)	39 (11.7)	Ref.
Agree	128 (51.6)	194 (58.4)	1.28 (0.71-2.31)
Strongly disagree	100 (40.3)	99 (29.8)	**1.97 (1.07-3.61)**
**The health board drives CA**			
Disagree/strongly disagree	80 (33.8)	125 (39.8)	Ref.
Agree	123 (51.9)	163 (51.9)	1.17 (0.81-1.69)
Strongly agree	34 (14.3)	26 (8.3)	**2.04 (1.14-3.65)**
**Participates in activities in the neighbourhood where working**	138 (55.0)	83 (24.6)	**3.75 (2.64-5.33)**
**Collaborates with an NGO or civic entity**	151 (59.9)	133 (39.7)	**2.27 (1.62-3.17)**
**Freedom to organise timetable**	163 (65.7)	157 (47.7)	**2.10 (1.49-2.95)**
**Possibility colleagues will cover while performing CA**	99 (40.6)	79 (23.9)	**2.16 (1.51-3.11)**
**Can depend on colleagues to collaborate on CA**	215 (86.3)	222 (67.7)	**3.02 (1.96-4.63)**

Data from Spain 2009. Bold-face: Chi-squared $p \leq 0.05$

Due to missing data, 100 (16.8%) professionals were excluded from the multivariate analysis, leaving a total of 497. There were no significant differences between the excluded and included individuals. The final multivariate model (Table 4) shows that the following

For peer review only - http://bmjopen.bmj.com/site/about/guidelines.xhtml

factors were significantly associated with health-promoting community activities: profession (social workers do the most, followed by nurses and then physicians); specific training in community activities; motivation; social sensitivity (collaboration with NGOs and participation in neighbourhood activities); perception of support and coverage by colleagues while doing community activities; years working in the health care team; and being a nursing-student tutor.

For peer review only

For peer review only - http://bmjopen.bmj.com/site/about/guidelines.xhtml

Table 4. Multivariate model of performing health-promoting community activities (CA)

Variable	General model (n=497) OR (95% CI)
**Profession**	
Nurse	Ref.
Doctor	0.44 (0.24-0.82)
Paediatrician	1.55 (0.69-3.46)
Social worker	25.26 (2.53-251.9)
**Specific training in CA**	1.91 (1.17-3.11)
**Collaboration with NGO or civic entity**	1.91 (1.17-3.13)
**Possibility of work covered by colleagues**	1.80 (1.08-2.99)
**Support from colleagues in doing CA**	2.92 (1.49-5.73)
**Motivated to perform CA**	
Disagree or strongly disagree	Ref.
Agree	2.29 (1.28-4.08)
Strongly disagree	3.74 (1.85-7.54)
**Participates in activities in the neighbourhood**	3.06 (1.86-5.05)
**Years at health centre**[†]	1.06 (1.03-1.09)
**Nursing-student tutor**	1.96 (1.09-3.53)
**Model Validity**	**Value**
**Chi^2**	221.338
**Nagelkerke R squared**	.483

Discussion

Our study of the participation of health care workers in health-promoting activities in the community suggests that factors of the individual health care professional were more

For peer review only - http://bmjopen.bmj.com/site/about/guidelines.xhtml

important than those of the health care team or the community. In particular, the specific profession, attitudes, and opinions of an individual seemed to be crucial. Professional workload, which was one of the more mentioned factors by the professionals in others studies, does not have any impact when adjusted by other individual factors. These results confirms the frAC Project[29] hypothesis and those of other authors[21,28]

Our assessment of variables related with health care team participation in health promoting community activities suggests that team characteristics had greater impact than community characteristics. One exception was the socio-economic level of the community. This makes sense in the context of the Spanish health care system, which attempts to offer equitable care to people according to their needs and community interventions are important tools to reduce inequalities in health care[35]. It should be noted that we found no relationship between the level of social interaction in the community and the implementation of community activities, in contrast with the findings of other authors[16-17].

Our results showing the important role of nurses in health promoting community activities are consistent with those of other authors[13-14,17,36] who highlighted the role of nurses in the development of community activities. This result is expected because nurses usually receive more training in community care. Another crucial group is social workers. Previous research reported the importance of social workers[13], but their role in the community varies in different regions of Spain. Their presence seems fundamental for development of health-promoting community activities in regions where they have significant roles in the community. In Spain, community work is generally not provided as part of primary health care; instead, it is relegated to professional volunteers or specific programs. Thus, our results recognize the importance of work by nurses and social workers and points to the possibility that their roles could be modified or clarified. We also found that health centres that train undergraduate nurses have a lower level of community activities but that professional nurse-

For peer review only - http://bmjopen.bmj.com/site/about/guidelines.xhtml

trainers have a greater involvement in health promoting community activities. This apparent contradiction may be resolved by considering two factors. First, the variable at the health centre level included training in various disciplines (from nursing to physiotherapy) without distinction, even though there is clearly difference among these in communitarian tradition. Second, the need for increased training would make it difficult for the health team to engage in community activities although, among the cases trainers carried out more activities than others. Health care workers who worked for more years at a centre were also more likely to perform community activities. This may be because medium- and long-term projects require continuity and stability, and it is more difficult for professionals who continually change centres or have temporary contracts to participate in health promoting community activities.

The pressures of working in health care and the heavy work burden were the main arguments offered by professionals when asked about their low participation in health promotion and prevention activities[19,22,24-27]. Our bivariate analysis supported this relationship, although the multivariate analysis did not. This implies that the professionals' perception was incorrect. In fact, the pressure of working in health care and the heavy work burden are not associated with low participation in health promoting community activities.

Our results indicated the importance of the opinions and attitudes of professionals when performing health promoting community activities, in accordance with other studies[15,21-22]. In fact, all variables regarding the opinions and attitudes of health care workers were significantly different between cases and controls. This suggests that individuals who perform community activities have similar attitudes: they employ a biopsychosocial[12,37] practice model, are more oriented towards health promotion, show recognition of the need for citizen participation in health services, and are more left-leaning in ideology. We also found that social sensitivity was an important variable in the model for professionals. This variable measures involvement of the professional with the community beyond consultation work. It is

For peer review only - http://bmjopen.bmj.com/site/about/guidelines.xhtml

not clear how some practices, responsibility for which is stipulated in primary care legislation and which should be similar for all professionals to assure fairness within the system, can be affected by a professional's personal discourse. Most health care professionals that we interviewed – both cases and controls -- said that their community roles were not well defined. This suggests that greater priority should be given to develop health promoting community activities in primary health care, and to clarify the role and responsibility of every professional about this question.

We found that having support within the health care team is associated with performing health promoting community activities. This finding should be highlighted, because all the professionals in this study were selected from the centres that engage in community activities so that, presumably, all cases can count on some support from their centres. It may be that some professionals were unaware of what their colleagues were doing, or that there were barriers to working with particular colleagues. Regardless of the explanation, this result suggests that application of the teamwork model[38], an assumed key feature of primary health care, has certain weaknesses.

Strengths and limitations

This study had an observational design, so we could not definitively establish causal relationships. Similarly, our definition of "health promoting community activities" may be a limitation because this concept can be defined or perceived in different ways; we made an attempt to control for this by use of a definition generated by expert consensus, which we then transformed into an algorithm. The questionnaires we used were *ad hoc* and some of the data regarding team variables, such as degree of social interaction in the community and the relationship between professionals, were provided by team managers. This may have led to a bias in information gathering due to a lack of knowledge or other unknown factors. Some variables that we did not examine could have explained participation in community activities,

For peer review only - http://bmjopen.bmj.com/site/about/guidelines.xhtml

such as support from primary care management, the health care administration and the centres' own coordinators or nursing managers. It would be valuable to assess the effect of these variables in future studies of the effect of team activities and priorities.

The main strengths of this study are that it examined a facet of health care that is relatively unexplored; the study subjects were from 5 different regions of Spain; the sample size was large; and it considered many distinct factors simultaneously. Furthermore, our design of the second study adjusted the effect of team and community variables matching cases and controls by teams, so a multilevel analysis was not necessary. Given the extension of the achieved sample size and the inclusion of regions with different size, our results could be generalized to Spanish primary health care.

Implications for health care practice

Health care administrators who want to develop health-promotion activities for the community must provide visible and formal support for community initiatives, define better the expected communitarian tasks of each health professional, and provide job stability for all participating workers. Specific training is also important for the formation of health-promoting community activities. It may seem that attitudes, social sensitivity, or personal opinions are not modifiable; however, in our opinion, it is possible to modify these factors during undergraduate education. So, community perspective and their associated responsibility should be included in healthcare workers training

Conclusions

A key responsibility of primary health care is provision of health-promoting community activities, although not every team or professional makes them. We describe some factors related with this implication, such as to work in a low socio-economic level community or to have specific training, support, motivation. social sensitivity or more years in the health team. There were also differences among professional categories, highlighting

For peer review only - http://bmjopen.bmj.com/site/about/guidelines.xhtml

For peer review only

nurses and social workers contributions. The pressures perceived by the professionals were not associated with involvement in community activities.

Acknowledgements

We want to express thanks to Magdalena Esteva for her patient reading of the manuscript.

Competing interests

All authors have completed the ICMJE uniform disclosure form at www.icmje.org/coi_disclosure.pdf and declare: no financial relationships with any organisations that might have an interest in the submitted work in the previous three years; no other relationships or activities that could appear to have influenced the submitted work

Funding

This study was supported by a grant from the Carlos III Health Institute (PI07/90383; PI07/90925; PI07/90636). It also received support from the Health Promotion and Preventive Activities in Primary Health Care Research Network (IAPP network RD 06/0018/), the ERDF funds, and the Institut de Salut de Palma (IDISPA). SM was contracted with the aid of a grant to stabilise employment in health research from the Carlos III Health Institute and benefited from a grant for further studies from the same institution. All institutions are public and did not have any role in study design, analysis, or publication.

Contributors

SM was the lead investigator, and was responsible for conception and design of the study, analysing data and writing the manuscript. **MR** has the original idea of the study. **SM, JR, JLRG, IMG, CBBA,** and **LES**, coordinated the field work in everyone of the regions involved. SM, JR, MJM, EZO, CBBA, LES, CV, MLLBA, TPJ, DMPM, MLLS, JLRG, RBV, CSV, MRB, MCV, MBC, ESF, MBD, IMG, BBR, AAL, SBC, ACT, BOB, RMB, PMP, MEO, MJPJ, MR contributed to the design and development of the study, have read the manuscript critically, made contributions and approved the final version.

For peer review only - http://bmjopen.bmj.com/site/about/guidelines.xhtml

Data Sharing Statement: Questionnaires and anonymised data are available on demand.

For peer review only

For peer review only - http://bmjopen.bmj.com/site/about/guidelines.xhtml

References

1. World Health Organisation. **The Ottawa Charter for Health Promotion**, Health Promotion. 1986 1 (4): i-v.

2. World Health Organization. **Health Promotion Glossary.** WHO 1998. [http://apps.who.int/iris/bitstream/10665/64546/1/WHO_HPR_HEP_98.1.pdf]

3. World Health Organisation: **Declaration of Alma Ata.** *WHO* 1978. [http://www.who.int/hpr/NPH/docs/declaration_almaata.pdf]

4. World Health Organisation: **Declarations of Jakarta** WHO 1997. [http://www.who.int/hpr/NPH/docs/jakarta_declaration_sp.pdf]

5. March S, Torres E, Ramos M. **Adult community health-promoting interventions in primary health care: A systematic review.** *Prev Med* 2015; 76 (2015) S94–S104

6. Ley General de Sanidad 14/1986, de 25 de abril. Boletín Oficial del Estado,nº102,(29/4/1986).

7. Real Decreto 1030/2006 del 15 de septiembre por el que se establece la cartera de servicios comunes del Sistema Nacional de Salud y el procedimiento para su actualización. *Boletín Oficial del Estado*,nº222,(16-9-2006).

8. Guldan GS: **Obstacles to community Health promotion**. *Soc sci med* 1996, **5**:689-695.

9. Irigoyen J: **La participación comunitaria en salud: Una reformulación necesaria.** In: *Nuevas perspectivas en Atención Primaria de Salud.* Edited by Aranda. Madrid: Díaz de Santos; 1994:183-221.

10. Gérvas J, Pérez-Fernández M, Palomo-Cobos L, Pastor-Sánchez R: **Veinte años de reforma de la Atención Primaria en España. Valoración para un aprendizaje por acierto/error.** Madrid: Ministerio Sanidad y Consumo 2005. [http://equipocesca.org/new/wp-content/uploads/2010/08/20-anos-de-reforma-de-la-ap-en-espana.pdf]

For peer review only - http://bmjopen.bmj.com/site/about/guidelines.xhtml

11. Regato P, Domínguez J, Sancho M: **Coordinación sanitaria y abordaje comunitario en atención primaria**. In: *La Atención Primaria de Salud en España y en sus comunidades autónomas.* Coordinated by Navarro V, Martín-Zurro A. Barcelona: Semfyc; 2009:149-168.

12. Turabián JL, Pérez B: **Grandes misterios. ¿Puedes ver al monstruo del lago Ness?: El modelo biopsicosocial y las actividades comunitarias.** *Aten Primaria* 2007, **39**:261-4.

13. March S, Soler M, Miller F, et al: **Variabilidad en la implantación de las actividades comunitarias de promoción de la salud en España.** *An Sist Sanit Navar.* In press.

14. Sáez S, Marqués F, Pallarés J, Serrano J, Orrio C, Martínez V: **Perfil de los profesionales de la salud de Lleida respecto a la educación para la salud (EpS).** *Aten Primaria* 1999, **24**:157-161.

15. Pérez-Franco B, Turabián JL, Magaña E, et al: **Factores asociados a la participación comunitaria en la atención primaria de la provincia de Toledo.** *Aten primaria* 1993, **12**: 339-344.

16. MJ Aguilar Idáñez: **La participación comunitaria en salud: ¿Mito o realidad?** Madrid: Díaz de Santos; 2001.

17. López E, Forcada C, Miller Frederick: **Factores que facilitan y dificultan el desarrollo de los proyectos comunitarios. Estudio observacional de la red AUPA de centros de atención primaria de Cataluña.** *Aten primaria* 2010, **42**:218-25.

18. Duro JC: **Actitudes de los profesionales de atención primaria de Madrid sobre los grupos.** *Aten Primaria* 2005, **36**:71-7.

19. Díaz G: **Factores relacionados con la práctica de educación sanitaria por los médicos de Atención primaria de Galicia.** *Rev Esp Salud Pública* 2001, **75**:529-540.

For peer review only - http://bmjopen.bmj.com/site/about/guidelines.xhtml

20. Maheux B Dufort, F Lambert J et al: **The professional attitudes and clinical practices of men and women generalists.** *Can Fam Physician* 1989, **35**:59-63.

21. Maheux B, Pineault R, Lambert J et al: **Factors influencing physicians' preventive practices.** *Am J Prev Med* 1989, **5**:201-206.

22. Myrand A, Beehler G, Kuo C, Mahoney M: **Explaining the de-prioritization of primary prevention: Physicians' perceptions of their role in the delivery of primary care.** *BMC Public Health* 2003, **3**:15. doi:10.1186/1471-2458-3-15.

23. Walter U, Flick U, Neuber A, et al: **Putting prevention into practice: qualitative study of factors that inhibit and promote preventive care by general practitioners, with a focus on elderly patients.** *BMC Family practice* 2010, **11**:68. doi:10.1186/1471-2296-11-68

24. Puig ribera A, McKenna J, Riddoch C: **Attitudes and practices of physicians and nurses regarding physical activity promotion in the Catalan primary health-care system.** *Europ J Public Health* 2005, **15**:569–75.

25. Wilhemsson S, Lindberg L: **Health promotion: Facilitators and barriers perceived by district nurses.** *Int J Nurs Practice* 2009, **15**:156-163.

26. Rubio-Valera M, Pons-Vigues M, Martinez-Andres M et al. **Barriers and Facilitators for the implementation of primary prevention and health promotion activities in primary care: A Syntethesis through meta-etnography.** Plos one 2014;9:e89554. Doi: 10.1371/journal.pone.0089554.

27. Badertscher N, Rossi PO, Rieder A et al: **Attitudes, barriers and facilitators for health promotion in the elderly in primary care.** *Swiss Med Wkly* 2012;**142**:w13606.

28. Maheux B, Pineault L, Béland F et al: **Factors influencing physicians' orientation toward prevention.** *Am J Prev Med.* 1987, **3**:12-8.

For peer review only

For peer review only - http://bmjopen.bmj.com/site/about/guidelines.xhtml

29. March S, Ripoll J,Ruiz-Giménez JL, et al: **Observational study on factors related to health-promoting community activity development in primary care (frAC Project): a study protocol.** *BMJ Open* 2012, **2**. e001287 doi:10.1136/bmjopen-2012-001287.

30. March S, Jordán M, Montaner I et al. **¿Qué hacemos en el barrio? Descripción de las actividades comunitarias de promoción de la salud en atención primaria: Proyecto frAC.** Gac Sanit 2014; **28**:267-73.

31. March S, Ramos M, Soler M, Ruiz-JiménezJL, Miller F, Domínguez J, et al: **Revisión documental de experiencias de actividad comunitaria en atención primaria de salud.** *Aten Primaria* 2011, **43**:289-296.

32. T Martínez-cañavate: **Modelo multinivel explicativo de la utilización de las consultas de atención primaria en Andalucía**. *Phd tesis.* Granada university, faculty of medicine; 2007. [http://hera.ugr.es/tesisugr/16905234.pdf]

33. Mira JJ, Llinás G, Gil V, Orozco D, Palazón I, Vitaller J: **Validación de un instrumento para identificar estilos de práctica profesional del médico de atención primaria.** *Aten Primaria* 1998, **21**:14-22.

34. Rey del Castillo P: **Nota metodológica sobre los indicadores del barómetro.** *Rev Española de Investigaciones Sociológicas* 2004, **108**:151-78.

35. Comisión para reducir las desigualdades en salud en España: **Propuesta de políticas e intervenciones para reducir las desigualdades en salud en Espanya**. *Gac Sanit* 2012. doi:10.1016/j.gaceta.2011.07.024.

36. Hogg W, Dahrouge S. Russell G et al: **Health promotion activity in primary care: performance of models and associated factors.** *Open medicine* 2008, **3**:165-73.

37. Engel GL: **The need for a new medical model: a challenge to biomedicine.** Science 1977, **196**:129–36.

For peer review only

For peer review only - http://bmjopen.bmj.com/site/about/guidelines.xhtml

38. Grumbach K, Bodenheimer T: **Can Health Care teams improve Primary Care practice?** JAMA 2004, **291**:1246-51.

For peer review only

For peer review only - http://bmjopen.bmj.com/site/about/guidelines.xhtml

STROBE Statement—Checklist of items that should be included in reports of ***case-control studies***

	Item No	Recommendation
**Title and abstract**	1	(*a*) Indicate the study's design with a commonly used term in the title or the abstract<br>OK. p1.
		(*b*) Provide in the abstract an informative and balanced summary of what was done and what was found<br>OK. p2
**Introduction**		
Background/rationale	2	Explain the scientific background and rationale for the investigation being reported<br>OK. p4-5
Objectives	3	State specific objectives, including any prespecified hypotheses<br>OK. p5
**Methods**		
Study design	4	Present key elements of study design early in the paper<br>OK. p5
Setting	5	Describe the setting, locations, and relevant dates, including periods of recruitment, exposure, follow-up, and data collection<br>OK. p6
Participants	6	(*a*) Give the eligibility criteria, and the sources and methods of case ascertainment and control selection. Give the rationale for the choice of cases and controls<br>OK. p6
		(*b*) For matched studies, give matching criteria and the number of controls per case<br>OK. p6.
Variables	7	Clearly define all outcomes, exposures, predictors, potential confounders, and effect modifiers. Give diagnostic criteria, if applicable<br>OK. p6-8.
Data sources/ measurement	8*	For each variable of interest, give sources of data and details of methods of assessment (measurement). Describe comparability of assessment methods if there is more than one group<br>OK. p6-8
Bias	9	Describe any efforts to address potential sources of bias<br>OK. p17-18
Study size	10	Explain how the study size was arrived at<br>OK. p8
Quantitative variables	11	Explain how quantitative variables were handled in the analyses. If applicable, describe which groupings were chosen and why<br>OK. p6-8
Statistical methods	12	(*a*) Describe all statistical methods, including those used to control for confounding<br>OK. p8-9
		(*b*) Describe any methods used to examine subgroups and interactions<br>OK. p8-9
		(*c*) Explain how missing data were addressed<br>OK. p8-9
		(*d*) If applicable, explain how matching of cases and controls was addressed<br>Not applicable
		(*e*) Describe any sensitivity analyses

For peer review only

		Not applicable
**Results**		
Participants	13*	(a) Report numbers of individuals at each stage of study—eg numbers potentially eligible, examined for eligibility, confirmed eligible, included in the study, completing follow-up, and analysed<br>OK. p11
		(b) Give reasons for non-participation at each stage<br>OK. p11
		(c) Consider use of a flow diagram<br>OK. It is not necessary.
Descriptive data	14*	(a) Give characteristics of study participants (eg demographic, clinical, social) and information on exposures and potential confounders<br>OK. Tables 1 and 2.
		(b) Indicate number of participants with missing data for each variable of interest<br>OK. Tables 1-3.
Outcome data	15*	Report numbers in each exposure category, or summary measures of exposure<br>OK. Tables 1-3.
Main results	16	(*a*) Give unadjusted estimates and, if applicable, confounder-adjusted estimates and their precision (eg, 95% confidence interval). Make clear which confounders were adjusted for and why they were included<br>OK. Tables 1-3.
		(*b*) Report category boundaries when continuous variables were categorized<br>OK. Tables 1-3.
		(*c*) If relevant, consider translating estimates of relative risk into absolute risk for a meaningful time period<br>OK. It is not necessary.

For peer review only

For peer review only - http://bmjopen.bmj.com/site/about/guidelines.xhtml

Other analyses	17	Report other analyses done—eg analyses of subgroups and interactions, and sensitivity analyses<br>OK. p8-9.
**Discussion**		
Key results	18	Summarise key results with reference to study objectives<br>OK. p14-15.
Limitations	19	Discuss limitations of the study, taking into account sources of potential bias or imprecision. Discuss both direction and magnitude of any potential bias<br>OK. p17-18.
Interpretation	20	Give a cautious overall interpretation of results considering objectives, limitations, multiplicity of analyses, results from similar studies, and other relevant evidence<br>OK. p15-18.
Generalisability	21	Discuss the generalisability (external validity) of the study results<br>OK. p17-18
**Other information**		
Funding	22	Give the source of funding and the role of the funders for the present study and, if applicable, for the original study on which the present article is based<br>OK. p19

*Give information separately for cases and controls.

Note: An Explanation and Elaboration article discusses each checklist item and gives methodological background and published examples of transparent reporting. The STROBE checklist is best used in conjunction with this article (freely available on the Web sites of PLoS Medicine at http://www.plosmedicine.org/, Annals of Internal Medicine at http://www.annals.org/, and Epidemiology at http://www.epidem.com/). Information on the STROBE Initiative is available at http://www.strobe-statement.org.

For peer review only
